# Developmental programmes drive cellular plasticity, disease progression and therapy resistance in lung adenocarcinoma

**DOI:** 10.1002/1878-0261.70263

**Published:** 2026-05-27

**Authors:** Kamila J Bienkowska, Stephany Gallardo, Nur S. Zainal, Leena Arora, Matthew Ellis, Maria‐Antoinette Lopez, Judith Austine, Sai Pittla, Serena J Chee, Aiman Alzetani, Emily C Shaw, Christian H Ottensmeier, Gareth J Thomas, Christopher J Hanley

**Affiliations:** ^1^ School of Cancer Sciences University of Southampton Southampton UK; ^2^ Department of Histopathology University Hospital Southampton NHS Foundation Trust Southampton UK; ^3^ Institute of Systems, Molecular and Integrative Biology (ISMIB) and Liverpool Experimental Cancer Medicines Centre University of Liverpool Liverpool UK; ^4^ Department of Thoracic surgery University Hospital Southampton NHS Foundation Trust Southampton UK; ^5^ Cancer Research UK and NIHR Southampton Experimental Cancer Medicine Centre Southampton UK

**Keywords:** cellular plasticity, developmental programs, lung adenocarcinoma (LUAD), therapy resistance, Type‐I interferon, TP53

## Abstract

Cellular plasticity is central to non‐small cell lung cancer (NSCLC) disease progression and linked to aberrant regulation of developmental programmes. Here, we investigated the contribution of two developmental programmes, alveogenesis (ALV) and branching morphogenesis (BM), to NSCLC disease progression. ALV and BM inversely correlated across multiple transcriptome datasets. In squamous carcinomas (LUSC), ALV suppression and BM activation were consistently observed relative to controls, but these features were not prognostic. In contrast, adenocarcinomas (LUAD) displayed heterogeneous BM activation, associated with poor overall survival in several observational cohorts (*n* = 5) and resistance to tyrosine kinase inhibitors or immune checkpoint blockade. Exome sequencing linked TP53 pathway mutations to BM activation in LUAD, which was validated in conditional Trp53 knock‐out mouse models. Single‐cell RNA‐sequencing combined with multiplexed immunohistochemistry showed LUAD BM activation reflected increased morphological grade with tumour cells transdifferentiating to a basal‐like cell state. Finally, 3D organotypic cultures identified type‐I interferon signalling as a driver of BM activation in TP53‐mutant LUAD. Collectively, our findings reveal a novel TP53‐interferon axis that promotes transcriptomic plasticity in LUAD, with important implications for biomarker and therapeutic target discovery.

## Graphical abstract

This study shows that lung adenocarcinomas exploit developmental branching morphogenesis to acquire a therapy resistant basal‐like tumour cell state. This process was found to be regulated by combined TP53 loss‐of‐function and type‐I interferon signalling, identifying a novel axis for biomarker and therapeutic target discovery.

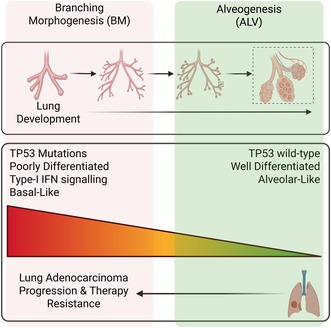

**Abbreviations**
ALV: Alveogenesis
AT1: Alveolar type 1 cell
AT2: Alveolar type 2 cell
BM: Branching morphogenesis
BM_CC: Branching morphogenesis—cell‐cycle‐related genes
BM_nonCC: Branching morphogenesis—non‐cell‐cycle‐related genes
GEMM: Genetically engineered mouse model
ICB: Immune checkpoint blockade
IFN I: Type I Interferon
LCMD: Laser Capture Micro‐Dissected
LUAD: Lung Adenocarcinoma
LUSC: Lung squamous cell carcinoma
NSCLC: Non‐small cell lung cancer
NTa: Non‐tumour alveoli
NTb: Non‐tumour bronchi
PCA: Principal component analysis
ssGSEA: Single‐sample gene set enrichment analysis
TKI: Tyrosine kinase inhibitor
TMB: Tumour mutational burden

## 1 Introduction

Lung cancer is the leading cause of cancer‐related deaths worldwide [1], despite recent advances in basic research and the implementation of oncogene‐ or immune‐targeted therapies [2]. Non‐small cell lung cancers (NSCLC) represent 85% of all lung cancer cases, which are subdivided into two main subtypes: lung adenocarcinoma (LUAD) and lung squamous cell carcinoma (LUSC) [3]. LUAD is the most common of these subtypes and the predominant type of lung cancer found in never‐smokers [3]. LUAD tumours typically develop in peripheral regions of the lung, mainly arising from oncogenic transformation of alveolar type 2 cells (AT2) [4]. LUSC originates from squamous airway epithelial cells predominantly found in more central regions of the lung [4].

Cell turnover in healthy adult lungs is low, but following injury, progenitor populations can become activated to proliferate and differentiate into one or more cell types [5, 6]. This increased epithelial plasticity has also been described in LUAD primary tumours, leading to loss of lineage fidelity and the emergence of mixed‐lineage or regenerative phenotypes [5, 7]. Furthermore, plasticity has also been associated with metastatic progression and histological subtype determination, involving transcription factors that regulate embryonic development (SOX2 and SOX9) [5, 8].

Normal lung development involves two independent and mutually antagonistic programmes: branching morphogenesis (BM) and alveogenesis (ALV) [9]. BM describes the process of coordinated proliferation and collective migration leading to the development of the airways and the proximal‐distal axis [10]. ALV involves the generation and maturation of alveoli, enabling efficient gas exchange [10].

The tumour suppressor gene TP53 is frequently genetically altered in NSCLC, with a particularly high prevalence found in LUSC (~ 81%) and relatively fewer in LUAD (~ 45%) [11]. The role of TP53 mutations in aberrant regulation of the cell cycle and apoptosis or senescence is well described, but it is also now appreciated that TP53 plays a wide‐ranging role in many oncogenic and biological processes, including involvement in regulating cellular plasticity [12]. For example, TP53 suppression enhances reprogramming to an induced pluripotent stem cell state [13] and in lung tissue regeneration, TP53 activity is required for AT2 progenitors to differentiate into AT1 cells [14]. TP53's tumour suppressive properties in NSCLC have also been shown to include restricting cellular plasticity [15].

Type I interferons (IFN‐I) have also been linked to tumour suppression and proposed as putative therapeutic targets [16]. However, IFN‐I signalling agonists have not shown clinical efficacy in the treatment of lung cancer [17, 18]. This is expected to be due to IFN‐I playing a pleiotropic and context‐dependent role in tumour development, progression and therapy resistance. For example, interferon‐related gene signatures have been shown to indicate resistance to chemo/radiotherapy [19, 20]; IFN‐I signalling has been shown to regulate cancer cell stemness [21, 22]; and IFN‐I signalling has been shown to contribute to adaptive resistance to EGFR inhibitors in NSCLC [23].

In this study, we performed a comprehensive analysis of transcriptomic (bulk tissue and single cell) datasets to determine whether aberrant regulation of developmental programmes (ALV and BM) influences human NSCLC disease progression and therapy resistance. Performing further *in silico* and experimental validation to elucidate the signalling pathways involved in this process.

## 2 Methods

### 2.1 Bulk tissue transcriptomic data processing and analysis

#### 2.1.1 Data access and processing

NSCLC TCGA RNA‐seq dataset (Firehose Legacy) was downloaded into RStudio using TCGAbiolinks package [26] and processed using the variance stabilising transformation implemented in DE‐Seq2 R package [27]. Raw RNA‐sequencing data from GSE103584 [28] was downloaded and processed to logarithmically normalised counts per million (lcpm) using recount3 R package [29]. Genes with few or no counts across samples were removed from the dataset using *filterByexprs* function (edgeR package [30]) with default parameters, and argument ‘group’ set to ‘Histopathology’. TMM (trimmed mean of M‐values) method was used to determine normalisation factors, and the data was prepared in the cpm (counts per million) format, then log‐transformed (lcpm) and scaled. Microarray datasets (GSE31552 [31], GSE72094 [32], GSE31210 [33], GSE68465 [34], GSE157009 [35], GSE17010 [35] and GSE4573 [36]) were log‐transformed (if not already performed prior) to obtain normal distribution and quantile‐normalised using NormalizeBetweenArrays function (limma package [37]). mRNA expression‐level z‐scores were downloaded for the IMPACT study cohort, accessed through the OncoSG Cancer Genomics Data portal (https://oncosg.skandlab.org/). Bulk tissue RNA‐sequencing data from ICB datasets were downloaded from GSE207422 [38] and GSE135222 [39] using the pre‐processed transcript per million (TPM) values, which were transformed to log2(TPM + 1) for analysis.

#### 2.1.2 ALV and BM gene signatures

Data from Chang et al. [9] used to generate ALV/BM gene signatures. The top 100 upregulated genes (ranked by log2 Fold Change) on embryonic day 14 (active branching morphogenesis) and embryonic day 19 (active alveogenesis) were used to generate the provisional BM and ALV signatures, respectively (Table S1). Significantly enriched (Bonferroni adjusted p value < 0.05) Gene‐ontology (GO) terms were identified using the *enrichGO* function from the ClusterProfiler R package [24], setting the background gene set to all genes reported in the Chang et al. study [9].

71 ALV genes were present in The Cancer Genome Atlas (TCGA) datasets, and 76 in scRNAseq datasets. 82 BM genes were present in both TCGA and scRNAseq datasets. Single‐sample Gene Set Enrichment Analysis (ssGSEA) [1] implemented in the GSVA (Gene Set Enrichment Variation) R package [25] was used to calculate enrichment scores of ALV and BM gene signatures per sample: Gene signatures (lists) of interest were set as ‘gset.idx.list’ argument, while a gene expression matrix containing all genes in the analysed dataset was set as ‘expr’ argument. The ‘method’ argument was set to ‘ssgsea’.

#### 2.1.3 Principal component analysis (PCA)

PCA was implemented using the *prcomp* function (base R). The results were visualised with the use of the factoextra package. The contribution of ALV/BM genes to each PC was assessed using a Fisher's exact test to measure enrichment among the top quartile of genes with the highest absolute loading coefficients.

#### 2.1.4 Survival analysis

Survival analysis was performed using the survival and survminer R packages. The *surv_categorize* function was used to group samples into BM_high and BM_low groups, based on ssGSEA scores. The cut‐point value used for stratification was calculated using the maximally selected rank statistics from the maxstat R package. 5‐year overall survival (OS) or progression‐free survival was used as the experimental outcome as indicated in the relevant Figures. Kaplan–Meier plots were prepared using *ggsurvplot*, and Log‐rank tests were performed to calculate p values. Cox proportional hazard regression model (*coxph* function) was fitted based on 5‐year overall survival data and with stage, age, and BM expression levels as covariates. Hazard ratios with 95% confidence intervals were visualised as a forest plot.

#### 2.1.5 Differential gene expression analysis

Differential gene expression analysis between BM‐low and BM‐high groups (stratification based on survival cut‐points described above) in LUAD microarray datasets was performed using the limma package [37]. First, a model matrix was constructed to model BM groups. Then, the *lmFit* function was used to fit a linear model to the normalised microarray data, followed by applying the Empirical Bayes statistics (*ebayes* function) for the differential expression of genes. Specific contrast of interest (high vs low) was defined using the *makeContrasts* function and was applied to the fitted model using the *contrast.fit* function. The *ebayes* function was re‐applied to these contrasts, and differentially expressed genes were identified using the *topTable* function. P values for significantly differentially expressed genes were adjusted for multiple testing using the False Discovery Rate (FDR) method.

Differential gene expression in TCGA LUAD BM‐high and BM‐low was performed using standard DESeq2 [27] protocol (while removing genes with counts < 10 and those detected in fewer than 2 samples), after grouping the TCGA samples based on their ssGSEA BM score cut‐point (ssGSEA score = 0.1881233).

#### 2.1.6 REACTOME pathway gene set enrichment analysis (GSEA)

Differentially expressed genes were organised into consensus ranked lists based on the sum of the log2(Fold‐change[FC]) * (1‐adj.*P*‐value) for each dataset analysed. Pathway analysis was performed using the clusterProfiler [24] and fgsea packages [40]. REACTOME pathways were accessed with the *reactomePathways* function.

To group enriched terms, Jaccard indices (JI) were calculated to enumerate the overlap between genes included in the leading edge for each term. 1‐JI was then used as a distance metric for hierarchical clustering, performed using Ward's method and k‐means clustering. Cluster core terms were identified by selecting the pathway with the lowest q‐value, largest set size, and the largest normalised enrichment score.

### 2.2 Human NSCLC single‐cell RNAseq data processing and analysis

#### 2.2.1 Data processing

Four single‐cell RNA‐sequencing datasets of NSCLC were analysed in this study: ‘Kim’ [41], ‘Czbiohub’ [42], ‘Zilionis’ [43] and TLDS (Target Lung Dataset) [44]. Only samples originating from primary lung tumours or normal tumour‐adjacent tissues were used to create the integrated dataset.

We used the Travaglini et al. [45] Human Lung Cell Atlas as a reference dataset to identify specific cell types within human lung tissues (Seurat's Label Transfer [46]). Single cells were assigned to appropriate cell types (meta lineages) if a prediction probability for this cell type was greater than 0.75.

Cells assigned as epithelial cells were extracted from each dataset and integrated using the Seurat R package [46] to implement the method described by Stuart et al. [47]. This was achieved, first by defining anchors connecting the datasets (using the *FindIntegrationAnchors* function) based on 30 principal components (PCs) and 2000 variable features. *The IntegrateData* function was run to integrate the datasets and correct for batch effects.

PCA was then performed on the scaled dataset. *FindNeighbors* and *FindClusters* functions were used to cluster cells, and *FindAllMarkers* function was utilised to calculate marker genes defining these clusters (Wilcoxon Sum Rank test, log2FC ≥ 0.25, min.pct = 0.1). Cell doublets (expressing genes typical to more than one cell lineage) were filtered out.

#### 2.2.2 Module scores

Seurat's *AddModuleScore* function was used to calculate single‐cell expression levels for developmental programme gene lists. Module scores were calculated for the developmental programmes on epithelial cells isolated from the complete (including metastatic sites) Kim [41], Maynard [42] and GSE207422 [38] datasets following log‐normalisation.

#### 2.2.3 Epithelial cluster identification and differential gene expression analysis

Cluster identification was performed on the ‘Integrated’ assay using Seurat's *FindClusters* function (resolution was set to 0.05). Gene markers of cell populations were identified with the use of Seurat's *FindConservedMarkers* function run on the ‘RNA’ assay (Wilcoxon test; log2FoldChange ≥ 0.1, genes expressed in at least 10% of cells in either group, grouping variable set to Dataset). Genes were considered statistically significant where the adjusted *p* < 0.05 in all datasets analysed. For analysis of all cells across epithelial subpopulations, all four datasets were analysed. For analyses comparing LUAD‐basal to LUAD‐Inflamed or LUAD‐AT2 subgroups, three datasets were analysed due to low cell numbers (< 100 cells) for these clusters in the TLDS dataset. GSEA was performed using the resulting differential gene expression statistics as described above for bulk tissue transcriptomics datasets.

#### 2.2.4 AT2 and basal cell prediction score (lineage Fidelity)

Label transfer analysis, as described by Stuart et al. [2], was used to calculate basal and AT2 prediction scores using Travaglini's [45] population of normal basal epithelial cells or AT2 cells as a reference, respectively. First, the *FindTransferAnchors* function was used to calculate anchors between the reference population and the integrated scRNAseq epithelial cells. Then, the *Transferdata* function returned basal/AT2 prediction scores for every queried cell (values ranging from 0 to 1). Due to low numbers of normal basal cells in the NSCLC datasets analysed (~50 cells total), we confirmed the accuracy of these prediction scores when applied to scRNA‐seq data from normal basal cells isolated from healthy human airways [48].

### 2.3 Mutational signatures and TP53/KRAS mutations

TCGA data containing mutation calling (MC3) [49] were downloaded into R and converted into maf objects using TCGAmutations and maftools [50] packages. LUAD samples were stratified into BM_high and BM_low groups on the cut‐point value from the survival analysis (ssGSEA score = 0.1881233). To extract mutational signatures present in the datasets, we first used the *trinucleotideMatrix* function (NFM package [51]) to extract single 5′ and 3′ bases flanking mutated sites, followed by *extractSignatures* that allowed signature identification. To calculate differential enrichment of a signature across LUAD groups, we used the contribution of each signature to the overall mutational profile of a sample (sum of all signatures = 1).

### 2.4 Murine model scRNA‐seq analysis

ScRNA‐seq data generated in the Marjanovic et al. study (GSE154989 [7]) were used to analyse ALV and BM activation in murine models of LUAD. All animal experiments were approved by the relevant institution's animal care committees, as described in the original publication [7]. Briefly, *Kras* ^*LSL‐G12D*^(K) and *Kras* ^*LSL‐G12D*^; *Trp53* ^*flox/flox*^ (KP) strains were used for inducing tumours in *Rosa26* ^*LSL‐tdTomato*^ C57BL/6 × Sv129 mixed background mice. Oncogene and/or tdTomato expression in AT2 cells was initiated using intratracheal administration of adenoviral particles, to induce Cre‐recombinase expression under the control of a 4.8 kb mouse surfactant protein C promoter sequence (AdSPC‐Cre). Tumours were collected 12‐, 18‐, 20‐ and 30‐weeks post AdSPC‐Cre administration; and control lung tissue was collected from *Rosa26* ^*LSL‐tdTomato*^ mice wild‐type for both Kras and Trp53 at 2‐ or 4‐weeks post AdSPC‐Cre administration. Lung tissue and tumours were dissected, disaggregated and tdTomato+ cells were isolated using fluorescence associated cell sorting (FACS). ScRNA‐seq was performed using a modified Smart‐Seq2 plate‐based protocol. Raw counts from the GSE154989 were downloaded and processed using the Seurat R package to perform log‐normalisation (*NormalizeData* function), aggregated single cell gene expression profiles for each individual tumour/sample (*AggregateExpression* function) and calculate module scores for murine ALV and BM signatures (*AddModuleScore* function).

### 2.5 Histology staining

#### 2.5.1 Sample collection and tissue microarray (TMA) construction

Archival formalin‐fixed paraffin‐embedded (FFPE) material was used to construct a TMA from 1‐mm cores (Aphelys Minicore 2, Mitogen). Regions for inclusion in TMA cores were selected by a pathologist (E. C. S.) to include areas of well‐differentiated and poorly differentiated (solid) LUAD (*n* = 30 cores from 10 patients for each), LUSC (*n* = 30 cores from 10 patients) and tumour adjacent non‐neoplastic lung tissue (*n* = 30 cores from 10 patients). Ethical approval was obtained through the UK National Research Ethics Service (NRES; Rec No. 10/H0504/32) and undertaken with the understanding and written consent of each subject. All tissue was collected from January 2017 to December 2022 and storage was handled by the Department of Cellular Pathology at University Hospital Southampton NHS Trust. In addition, this study was reviewed and approved by the University of Southampton Faculty of Medicine Ethics Committee and Research Integrity and Governance team. The study methodologies conformed to the standards set by the Declaration of Helsinki.

#### 2.5.2 Multiplexed immunohistochemistry and Histo‐cytometry analysis

Automated immunostaining (PT Link Autostainer, Dako) was performed in a Clinical Pathology Accreditation (UK) Ltd (CPA)‐accredited clinical cellular pathology department. Cyclical multiplexed immunohistochemistry was performed as described previously [44]. Briefly, serial four‐micron tissue sections from a TMA block were stained using the antibodies detailed in Table S2. CD31 staining was carried out using DAB substrate and used as fiducial points for image registration across imaging iterations. Subsequent stains were performed using AEC substrate to enable clearing between markers. Colour deconvoluted images were generated for each antibody stain representing blue (nuclear; haematoxylin), brown (CD31; DAB) and red (panel markers; AEC) signals. Target specific staining was thresholded by identifying topological maxima above the level of background signal, measured as the moving average over a 151 × 151‐pixel window (Original‐Background < 5 = 0). For the red signal, an additional condition was applied to account for the similarity in the colour profile of red and brown (red signal < (0.43*brown signal) = 0). Quality control (QC) checks for tissue integrity throughout cyclical staining were performed on the nuclear stain to identify tissue loss or registration errors: nuclear signal was identified from the first staining iteration (i [1]), then any nucleus with a signal missing in the final staining iteration (i[n]) was filtered out.

Histo‐cytometry analysis was performed using the filtered nuclear stain to segment individual objects. These regions were then grown up to 5.5 μm radius to represent simulated cell regions. Histo‐cytometry estimates of cellular expression levels were calculated as the fraction of pixels within simulated cell regions positive for staining once background levels had been subtracted. Pan‐CK+ cells were identified where the expression level exceeded 0.2. For all other markers, cells were classified as positive where the expression level exceeded 0.2 or the median + 1*mad (whichever value was higher). Cell phenotypes were identified by positivity for one or more relevant markers and negativity for alternative phenotype markers. Cells exhibiting positivity for markers of two separate phenotypes were classified as undetermined. Subpopulation enrichment was determined using Pearson's residuals from a chi square test assessing the number of each subpopulation as a proportion of the total number of epithelial cells across all three cores from each individual patient sample.

### 2.6 Cell culture and *in vitro* assays

H322 (NCI‐H322; ATCC CRL 5806, RRID:CVCL_1556) and A549 (ATCC CCL 185, RRID:CVCL_0023) cell lines were obtained from ATCC and authentication was performed by short tandem repeat (STR) profiling (≥ 98% matching; Northgene). Cell cultures were periodically confirmed to be mycoplasma free. H322 were grown in a 1 : 1 mix of DMEM (Dulbecco's Modified Eagle Medium) and RPMI (Roswell Park Memorial Institute) 1640, while A549 cells were maintained in DMEM/F12. All media were supplemented with 10% foetal bovine serum (FBS; FBS standard, PAN Biotech), 1% penicillin–streptomycin and 1% glutamine. All cells were maintained in a humidified incubator at 37 °C, 5% CO_2_.

#### 2.6.1 Collagen‐based 3D organotypic culture system

H322 and A549 spheroids were formed 2–3 days after adding 750 000 cells per well of a cell‐repellent 6‐well plate (CELLSTAR^®^, 657 970, Greiner bio‐one). 3 mg·mL^−1^ collagen was prepared by mixing rat tail type I collagen (Corning™ Collagen I, Rat; Corning™, 354 236) with 10X DMEM (D7777, Sigma) and sterile water. To neutralise the low pH of collagen gel, small volumes of concentrated NaOH were added (~ 1/1000^th^ of the volume of the gel until obtaining a salmon pink colour of phenol red included in DMEM). Neutralised gels were incubated for 30 min at 4 °C to allow fibril nucleation before they were mixed with spheroids. Spheroids were resuspended in gels at a concentration of 2 spheroids/μl of gel. 90 μL gels were seeded in wells of a 6‐well plate in triplicate and incubated for 1 h at 37 °C to allow the gels to fully set. Then, 4 mL of culture media supplemented with 1% FBS was added to each well, and the cells were incubated for 72 or 144 h. Type‐1 interferon treatment was performed using Universal Type I IFN (11200–1, R&D systems, Bio‐techne) at 10–1000 U/mL as indicated.

#### 2.6.2 Spheroid collection, RNA preparation, and cDNA synthesis

To collect spheroids, collagen gels were resuspended in 4 mg/mL collagenase IV (Gibco™ 17 104 019) and digested for 15 min, at 200 rpm, 37 °C. After digesting the collagen, spheroids were collected by centrifugation (10s, 100xg). RNA was extracted using ReliaPrep RNA Cell Miniprep System (Promega. Z6011). cDNA was prepared using RevertAid First Strand cDNA Synthesis Kit (Thermo Fisher Scientific, K1621) according to the manufacturer's instructions.

#### 2.6.3 Quantitative polymerase chain reaction ‐ qPCR

Gene expression changes (Fold Change) were investigated by qPCR using the ΔΔCt method [52]. Each reaction consisted of 10 ng cDNA, 1X PowerUp™ SYBR™ Green Master Mix (A25742, Thermo Fisher Scientific), and an appropriate concentration of primers as reported in Table S3 qPCR reactions were run using a QuantStudio 7 (Applied Biosystems). with the programme details set as follows: Hold stage: fast ramp to 95 °C, hold 20 s; PCR stage: 40 cycles: 95 °C hold 1 s, fast ramp to 60 °C, hold 20 s; Melt stage: fast ramp to 95 °C, hold 15 s, fast ramp to 60 °C, hold 1 min, slow ramp (0.05 °C/s) to 95 °C, hold 15 s. Primers were designed using previously described sequences (Origene; [*SOX2, SOX9*]) or using the primer blast tool [53]. *RPS14* was used as a housekeeping control (HC). All primer pairs were confirmed to have amplification efficiencies between 90–110%. Statistical analyses were performed by applying a one‐sample t‐test, to log2 fold‐change values generated from independent experiments, using FDR correction to adjust for multiple comparisons.

### 2.7 Statistical analysis

All statistical tests carried out were two‐sided as specified in the relevant Figure legends. All Boxplots are displayed using the Tukey method (centre line, median; box limits, upper and lower quartiles; whiskers, last point within a 1.5 × interquartile range) as implemented in the *geom_boxplot* function from the ggplot2 R package. Unless otherwise specified, statistical significance based on group comparisons was performed using the *geom_pwc* function from ggpubr package. Where asterisks were used to represent statistical significance, these represent the following *p*‐values: *: *p* ≤ 0.05, **: *p* ≤ 0.01, ***: *p* ≤ 0.001, ****: *p* ≤ 0.0001. Where stated in figure legends non‐significant comparisons (*p* > 0.05) are not shown; exact *p*‐values for all comparisons are reported in Table S4.

## 3 Results

### 3.1 Developmental gene expression programmes are a key source of transcriptomic variance in NSCLC

**Fig. 1:**
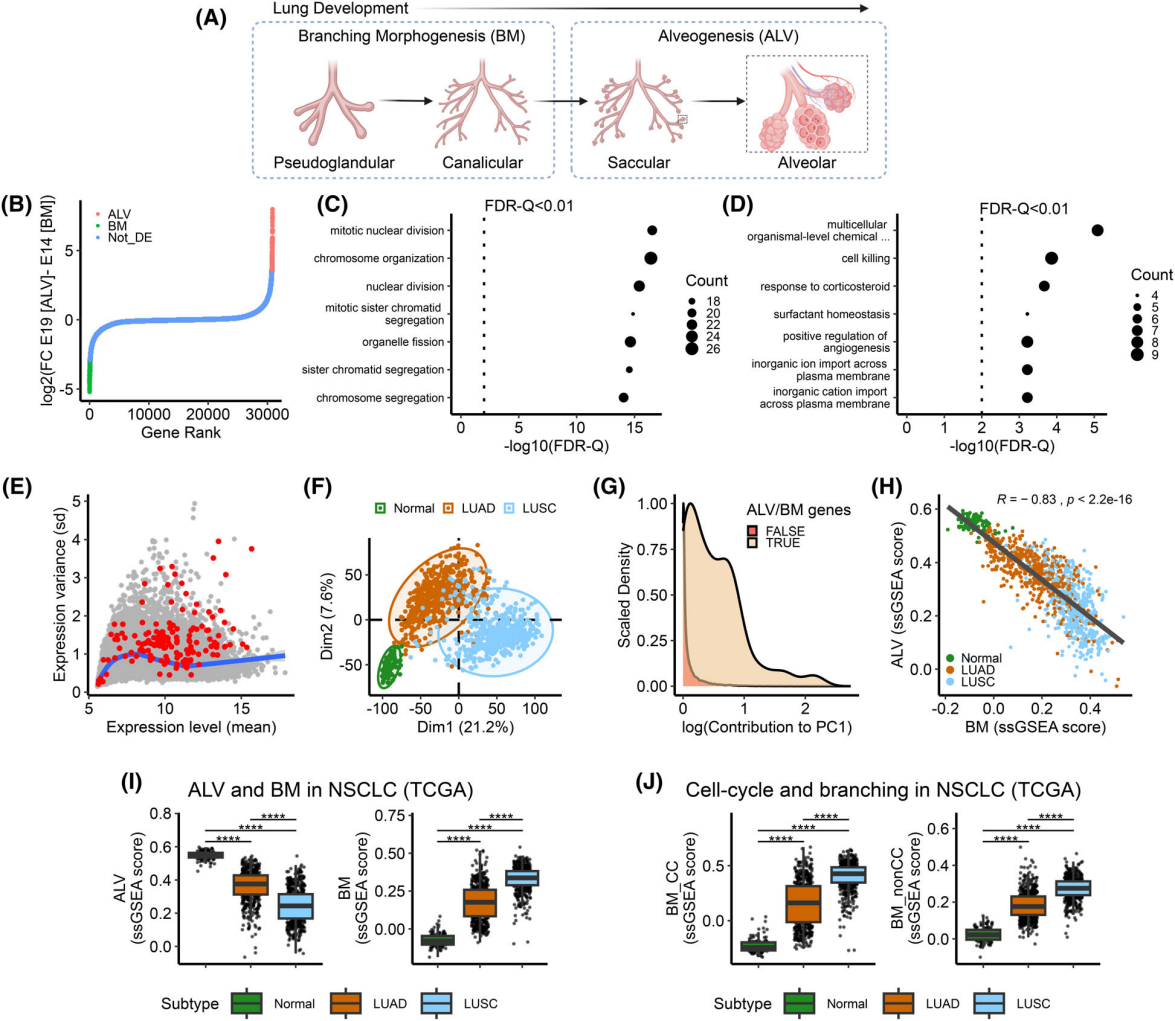
Developmental Alveogenesis (ALV) and Branching Morphogenesis (BM) programmes are associated with transcriptomic variance in NSCLC. (A) Schematic showing the different stages of lung development. (B) Dot plot showing the identification of genes differentially expressed by epithelial cells during murine developmental BM (embryonic day 14) and ALV (embryonic day 19). ALV/BM signatures were derived from genes with the highest Fold Change (coloured red or green respectively). (C, D) Dot plots showing GO terms enriched in ALV/BM genes, determined using a one‐tailed Fisher's exact test followed by FDR adjustment. (E) Scatter plot showing mean expression level and variance for all genes in NSCLC samples from TCGA (RNA‐Seq, *n* = 1128); BM/ALV genes shown in red. (F) PCA of NSCLC samples. (G) Histogram showing the contribution of ALV/BM genes to PC1. (H) Scatter plot showing ALV and BM scores across NSCLC samples; *R* = Spearman's correlation. (I, J) Boxplots showing enrichment scores in TCGA cohort grouped by histological subtype, calculated using ssGSEA: for ALV and BM signatures (I); and BM_cc (cell‐cycle‐related genes) and BM_nonCC (non‐cell‐cycle‐related genes) (J); asterisks represent FDR adjusted p‐values from pairwise Wilcoxon rank‐sum tests. ALV—Alveogenesis, BM—branching morphogenesis, NSCLC—non‐small cell lung cancer, PCA—principal component analysis, PC1—first principal component, TCGA—The Cancer Genome Atlas, FDR—false discovery rate, ****—*p* ≤ 0.0001.

We hypothesised that NSCLC plasticity was driven by the aberrant regulation of developmental programmes (BM and ALV) (Fig. 1A). To test this hypothesis, we identified genes that were differentially expressed in murine epithelial cells engaged in active BM (corresponding to embryonic day 14) vs active ALV (corresponding to embryonic day 19), using a publicly available microarray dataset [9] (Fig. 1B and Table S1). Functional annotation showed that the BM signature was significantly enriched in mitotic processes (Fig. 1C). In contrast, the ALV signature was associated with canonical AT2 cell functions, such as surfactant homeostasis, in addition to regulation of inflammatory responses, body fluid levels, and angiogenesis [54] (Fig. 1D).

We then analysed the expression of ALV and BM signatures in NSCLC, using The Cancer Genome Atlas (TCGA) RNA‐seq dataset, consisting of LUAD (*n* = 517), LUSC (*n* = 501), and control (non‐neoplastic) lung tissue samples (*n* = 110). ALV and BM genes exhibited high expression‐level standardised variance, implying an important role in NSCLC biology (Fig. 1E). PCA (Principal Component Analysis) showed components 1 and 2 separated the NSCLC samples by subtype (Fig. 1F) and genes from the ALV/BM signatures heavily influenced this separation (Fig. 1G and S1A,B). We also observed a negative correlation between enrichment scores for the ALV/BM signatures in NSCLC samples (Fig. 1H, *R* = −0.83, *p* < 2.2e‐16), consistent with their antagonistic roles in lung development [9]. The ALV signature was significantly downregulated in both tumour subtypes compared to control samples, whereas BM was upregulated. Furthermore, LUSC samples had significantly lower enrichment scores for ALV and higher scores for BM than LUAD (Fig. 1I).

Cell proliferation is fundamental to both developmental BM and cancer. To determine whether the association between BM and cancer extended beyond genes involved in this process we split the BM signature into genes associated with cell cycle, cell proliferation, cell division and cell replication gene ontology terms (BM_CC); and non‐proliferation‐associated genes (BM_nonCC), comparing enrichment scores for these new signatures across NSCLC samples. This showed both BM_CC and BM_nonCC signatures varied similarly between NSCLC subtypes (Fig. 1J).

These findings were validated using a Laser Capture Micro‐Dissected (LCMD) microarray dataset [31]: comparing the epithelial compartment from non‐tumour alveoli (NTa), non‐tumour bronchi (NTb), LUAD and LUSC (Fig. S1C–J). Additionally, we confirmed the negative correlation between ALV and BM signatures in multiple independent microarray datasets (LUAD [*n* = 3] and LUSC [*n* = 3]; Fig. S1K) [32, 33, 34, 35].

Given the variation in ALV/BM gene expression observed between NSCLC subtypes and non‐neoplastic alveolar/bronchial samples, we examined whether the tumour's location along the proximal‐distal axis influenced ALV/BM signature expression in NSCLC. This showed an increase in ALV and decrease in BM for peripheral tumours compared to central tumours (Fig. S1L). However, ALV/BM scores were not significantly different when comparing central and peripheral tumours from within LUAD or LUSC subtypes independently (Fig. S1L).

In summary, these analyses demonstrated that ALV and BM gene expression programmes strongly influenced transcriptomic variation in NSCLC. LUSC uniformly suppressed ALV and activated BM; whereas LUAD tumours were heterogeneous for BM activation, with some cases exhibiting ALV programme expression comparable to control tissue.

### 3.2 BM activation is associated with poor outcome in lung adenocarcinomas

**Fig. 2:**
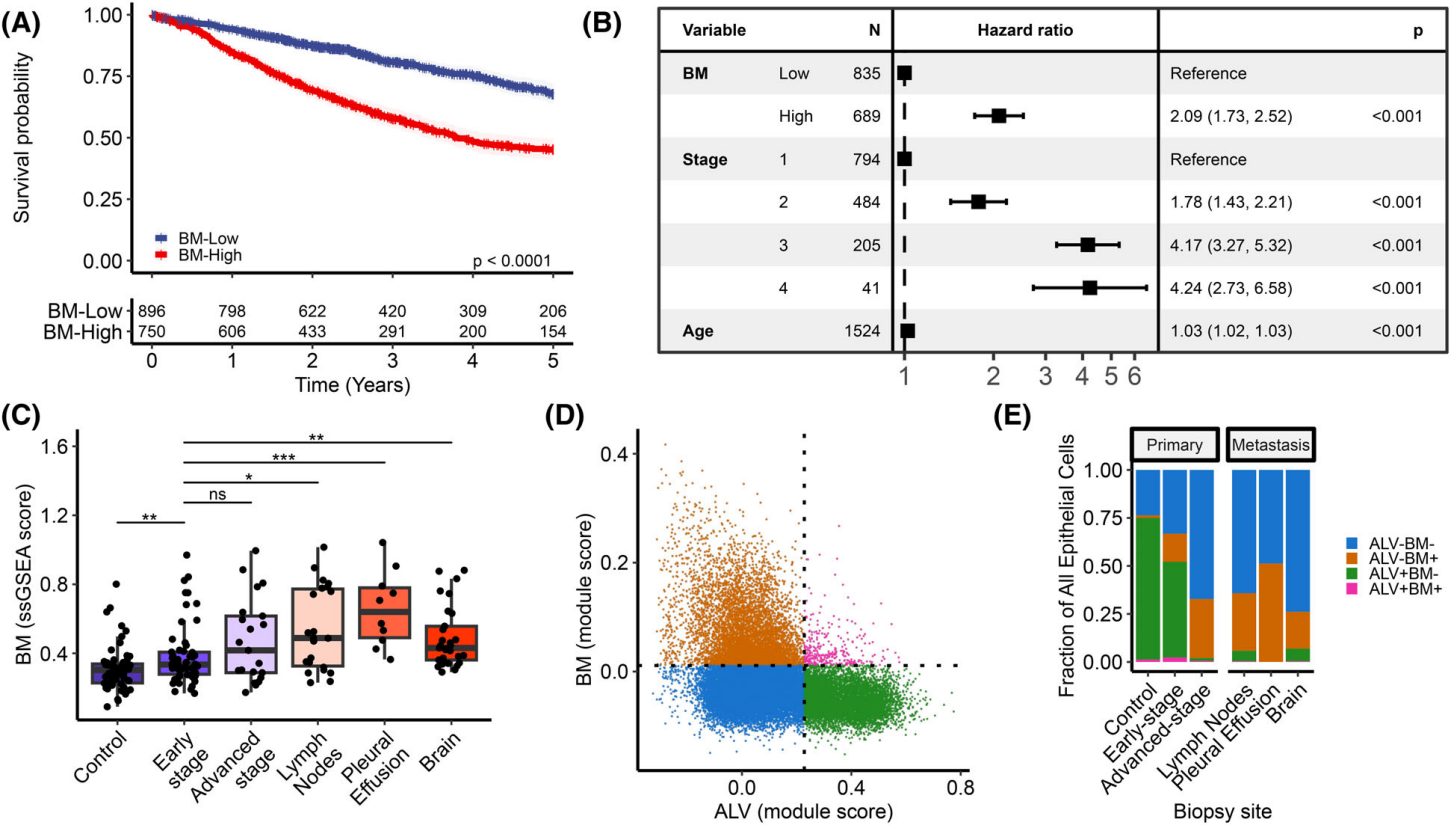
High expression of BM in LUAD predicts poor overall survival and is associated with disease progression. (A, B)—5‐year overall survival (OS) analysis in LUAD (*n* = 1646) stratified by BM expression across 5 combined LUAD datasets (TCGA, GSE72094, GSE68465, GSE31210, GSE103584): (A) Kaplan–Meier (KM) plot; (B) Forest plot showing multivariate Cox proportional hazards regression model coefficients (+/− 95% CIs) and adjusted p‐values. (C–E) BM expression in advanced and metastatic LUAD samples (from Kim et al., *n* = 204 samples and 36 467 cells): (C) Boxplots showing sample‐level epithelial BM ssGSEA scores, asterisks represent the FDR adjusted p‐values from Wilcoxon rank‐sum tests compared to early‐stage samples; (D) Scatter plot showing classification of epithelial cells based on ALV/BM expression, dotted lines represent signature positivity thresholds (median + 1 median absolute deviation); (E) Bar plot showing proportion of epithelial cells defined by ALV/BM classes in primary and metastatic LUAD. BM—branching morphogenesis, LUAD—lung adenocarcinoma, FDR—false discovery rate, ssGSEA—single‐sample gene set enrichment analysis, ns—*p* > 0.05, *—*p* ≤ 0.05, **—*p* ≤ 0.01, ***—*p* ≤ 0.001.

To determine the clinical impact of BM activation we investigated whether this gene expression programme was associated with survival rates in NSCLC patients, using ten independent datasets (*n* = 5 LUAD and 5 LUSC). In LUAD cohorts (TCGA, GSE72094, GSE31210, GSE68465, GSE103584 [28, 32, 33, 34] *n* = 1646 patients) BM activation identified patients with significantly reduced five‐year overall survival rates in both univariate analysis (Fig. 2A and S2A) and multivariate analysis ‐ adjusting for disease stage and age (Fig. 2B). In contrast, there was no consistent association with survival in LUSC cohorts [35, 36] (TCGA, GSE157009, GSE157010, GSE4573, GSE103584; Fig. S2B).

We next investigated whether BM activation increased during disease progression. For this analysis we utilised the Kim et al. dataset [41] where scRNA‐seq was performed on samples taken from different disease stages and anatomical sites. This showed that BM activation was significantly increased in epithelial cells from both local and distant LUAD metastases (Fig. 2C). Analysis at the single‐cell level showed that ALV and BM marker expression was mutually exclusive in LUAD tumour cells with both ALV‐/BM‐ and ALV‐/BM+ LUAD cells increasingly enriched in advanced stage primary tumours and metastases (Fig. 2D,E).

In summary, the BM gene expression programe was found to be a highly significant prognostic marker in bulk tissue LUAD samples, independent of disease stage. Furthermore, BM‐high tumours were shown to consist of a mix of tumour cells, which had uniformly suppressed expression of the ALV signature but only a subset displayed upregulated expression of BM markers.

### 3.3 TP53 loss of function is required for BM activation

**Fig. 3:**
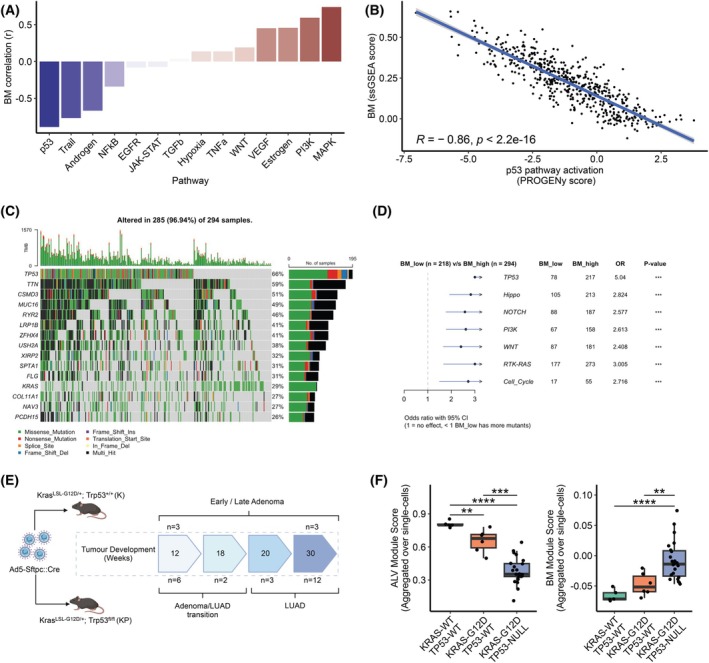
TP53 loss of function is required for BM activation. (A) Barplot showing correlation between BM ssGSEA score and PROGENy pathways signatures in TCGA LUAD. (B) Scatter plot showing the correlation between p53 pathway activation (PROGENy) and BM ssGSEA score in TCGA LUAD samples (*n* = 518). (C) Oncoplot showing top 15 mutated genes in LUAD BM‐high samples (TCGA). (D) Forestplot showing odds ratios for alterations in known oncogenic pathways in LUAD BM‐high samples. *P* values calculated with Fisher's exact test. (E, F) Analysis of ALV and BM expression in genetically engineered murine models (GEMMs – K [Kras^LSL::G12D/+^] and KP [Kras^LSL::G12D/+^;TP53^fl/fl^]) of LUAD, activated using adenoviral mediated Cre‐recombinase expression, driven by the Sftpc (AT2‐specific) promotor and analysed using scRNA‐seq (GSE154989). (E) Schematic showing the GEMMs used and time‐points analysed. Time shown in weeks. (F) Boxplot showing ALV/BM module scores in AT2/tumour cells from control lung or K and KP tumours measured by scRNA‐seq, each point represents the module score aggregated over single cells collected from the same tumour/lung tissue sample (*n* = 34); asterisks represent the FDR adjusted *P*‐values from pairwise Wilcoxon rank‐sum tests, non‐significant (*p* > 0.05) comparisons are not shown. ALV—alveogenesis, BM—branching morphogenesis, LUAD—lung adenocarcinoma, FDR—false discovery rate, ssGSEA—single‐sample gene set enrichment analysis, AT2 – alveolar type 2 cell, GEMM—genetically engineered mouse models, **—*p* ≤ 0.01, ***—*p* ≤ 0.001, ****—*p* ≤ 0.0001.

To investigate molecular mechanisms involved in regulating BM pathway activation we used the ProGENy (Pathway RespOnsive GENes for activity inference) database [55] to analyse the correlation between 11 curated pathway‐response signatures and the BM signature (Fig. 3A). This showed that the TP53 pathway had the most significant correlation with the BM signature, representing an inverse relationship (Fig. 3B, *R* = −0.86 and *p* < 2.2e‐16). Conversely MAPK (*R* = 0.67, *p* < 2.2e‐16) and PI3K (*R* = 0.66, *p* < 2.2e‐16) signalling had the strongest positive correlations with the BM signature.

Given our pathway analysis results, we hypothesised that genetic alterations to TP53 could lead to BM activation. In TCGA LUAD cohort TP53 mutations were enriched in BM‐high cases representing the most frequent mutation identified (66%; Fig. 3C); whereas TP53 mutations were less prevalent in BM‐low cases (30%; Fig. S3A). Furthermore, the odds of the TP53 signalling pathway being altered were significantly higher in the BM‐high LUAD (Odds ratio = 5.04, 95% CI, 3.4–7.53, adj.*p* = 6.63e‐17, Fig. 3D).

BM‐high LUAD tumours were found to have significantly higher tumour mutational burden (TMB) (Fig. S3B). Consistent with this, the tobacco smoking mutational COSMIC signature (SBS4) was also significantly enriched in the BM‐high compared to BM‐low LUAD tumours (Fig. S3C).

KRAS and EGFR mutations typically represent the most frequent oncogenic drivers observed in LUAD [56]. KRAS mutations were consistently observed in both BM‐high (29%) and BM‐low (31%) LUAD samples. Analysis of BM levels in KRAS and EGFR mutant LUAD samples with wild‐type TP53 had consistently low levels of BM activation, which were increased in TP53 mutant cases (Fig. S3D,E).

These observations suggested that irrespective of oncogenic driver mutations, TP53 loss of function was associated with increased BM. To test whether TP53 loss of function was directly responsible for BM activation, we used scRNA‐seq analysis of AT2 cells from adenoma/LUAD tumours isolated from Kras^LSL::G12D/+^; Trp53^+/+^ (K) or Kras^LSL::G12D/+^; Trp53^flox/flox^ (KP) genetically engineered mouse models [7] (GEMMs; Fig. 3E). This analysis demonstrated that simultaneous Kras activation and Trp53 deletion cause significantly decreased ALV expression and increased BM activation compared to tumours with Kras activation and wild‐type Trp53 or control lung tissue (Fig. 3F and S3F,G). Notably, Kras activation in the presence of wild‐type Trp53 was sufficient to induce a significant (*p* = 0.009) decrease in ALV expression compared to control tissue but failed to elicit a significant increase in BM (*p* = 0.052; Fig. 3F).

In summary, TP53 loss of function was found to be a critical event in BM activation irrespective of oncogenic driver mutations. BM‐high LUAD tumours were also commonly found to have higher tumour mutational burden compared to BM‐low LUAD, driven (at least in part) by smoking‐related mutational signatures.

### 3.4 BM activation is associated with targeted‐therapy resistance in lung adenocarcinomas

**Fig. 4:**
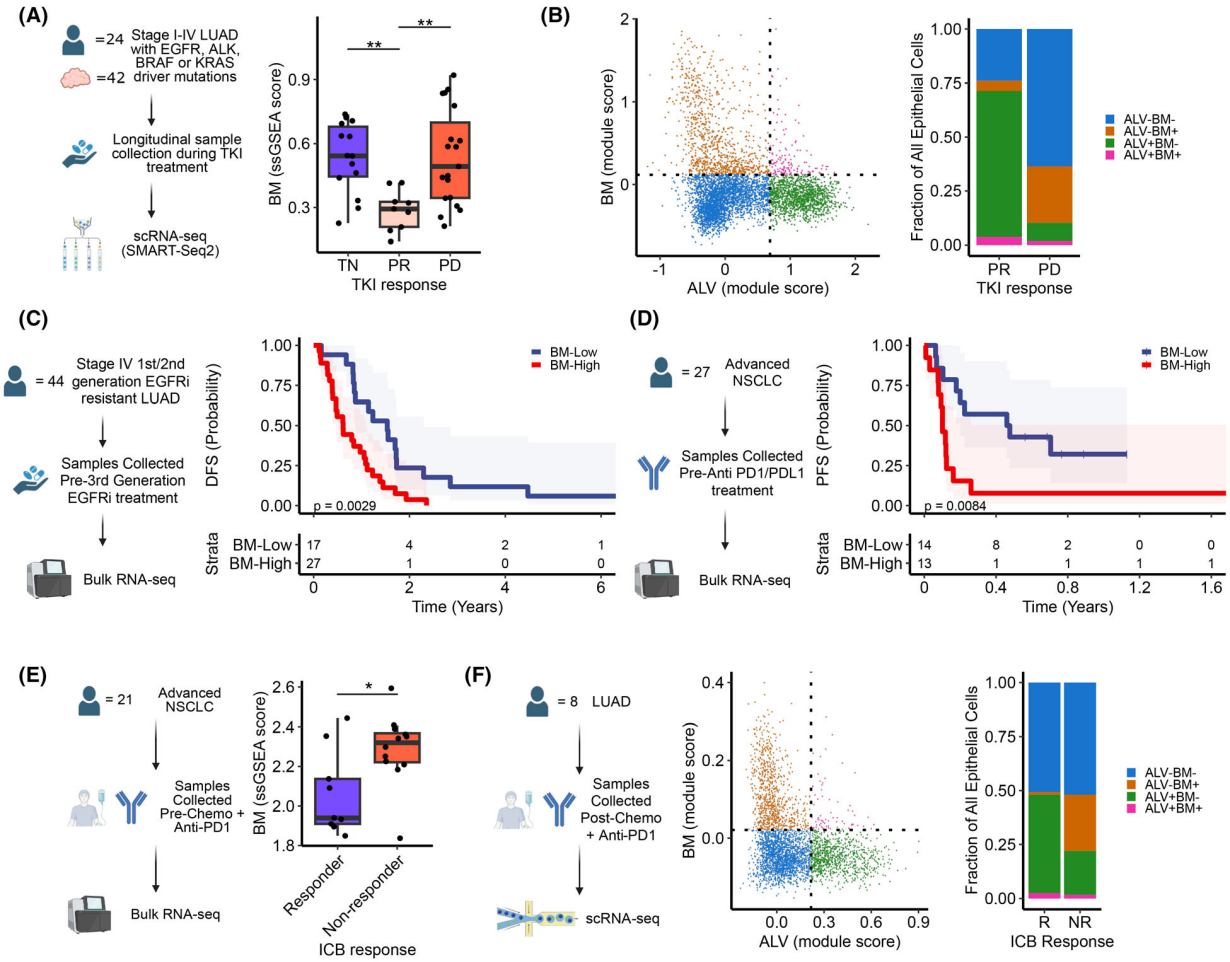
High expression of BM is associated with resistance to therapies. (A) BM expression in TKI‐treated LUAD samples (from Maynard et al., *n* = 42 samples from 24 patients); TN—treatment naïve, PR—partial response, PD—progressive disease), asterisks represent FDR adjusted *p*‐values from pairwise Wilcoxon rank‐sum tests, non‐significant (*p* > 0.05) comparisons are not shown. (B) Scatter plot showing classification of epithelial cells (from Maynard et al., *n* = 3906 cells) based on ALV/BM expression, dotted lines represent signature positivity thresholds (median + 1 median absolute deviation) and Bar plot showing proportion of epithelial cells defined by ALV/BM classes in LUAD samples grouped by response to TKI. (C) KM plot showing disease‐free survival (DFS) of patients receiving EGFR inhibitors (IMPACT study; from Chua et al., *n* = 44) stratified based on pre‐treatment BM expression. (D) KM plot showing progression‐free survival (PFS) of patients with advanced NSCLC receiving anti‐PD‐1/PD‐L1 (from Jung et al., *n* = 27) grouped by BM expression. *P*‐values (in C and D) were calculated using the Log‐Rank test. (E) BM expression in NSCLC patients receiving chemotherapy treatment combined with anti‐PD1, grouped by response to treatment (from Hu et al., *n* = 21); Responders classed as patients with major pathologic response), showing the Wilcoxon rank‐sum test *p*‐value. (F) as per panel B for LUAD samples (from Hu et al., *n* = 8 samples and 3804 cells). ALV—alveogenesis, BM—branching morphogenesis, TKI—Tyrosine kinase inhibitor, LUAD—lung adenocarcinoma, NSCLC—non‐small cell lung cancer, ** – *p* ≤ 0.01.

Loss of alveolar features has been linked to tyrosine kinase inhibitor (TKI) resistance [42, 57]. Given the inverse relationship between ALV and BM we suspected that BM activation may also be involved in TKI resistance. To investigate this, we first analysed the Maynard et al. dataset [42], where longitudinal scRNA‐seq analysis was performed on samples collected from patients undergoing a range of TKI treatments (Fig. S4A,B). This showed low levels of BM activation in tumour cells from partial response (PR) samples, which were significantly increased in samples with recurrent progressive disease (PD) (Fig. 4A). These changes were not simply associated with increased stage in recurrent samples as a similar increase was observed when only stage IV cases were analysed (Fig. S4C). Single‐cell analysis showed that both ALV‐BM‐ and ALV‐BM+ LUAD cells were increased in samples from recurrent progressive disease (Fig. 4B).

To examine the role of ALV and BM programmes in TKI resistance further we focussed on EGFR mutant LUAD, as this is the most common actionable driver. EGFR inhibitor (EGFRi) resistance can occur through on‐ or off‐target mechanisms. T790M alterations are a common on‐target resistance mechanism to 1st or 2nd generation EGFRi, which can now be targeted by 3rd generation inhibitors, and loss of alveolar lineage fidelity has been associated with off‐target resistance [57]. The IMPACT study performed RNA‐seq analysis on a cohort of 1st/2nd generation EGFRi resistant (stage IV) LUAD patients with mixed T790M status, and then examined the efficacy of 3rd generation EGFRi [57]. In this cohort BM activation was increased in samples lacking T790M alterations (Fig. S4D). BM‐high samples also had poorer disease‐free survival rates when treated with 3^rd^ generation EGFRi (*n* = 44, *p* 0.0029; Fig. 4C).

Notably, BM activation was more effective in stratifying patient response rates than T790M status in the IMPACT cohort and remained a significant prognostic indicator even in cases with T790M alterations (Fig. S4E,F). Furthermore, when combining the two variables in a multivariate cox regression model, BM activation was the only independently significant predictor of survival rates (HR [95% CIs] = 2.36 (1.13–4.91), adj.P = 0.02, Fig. S4G).

In summary, these results show that BM activation commonly occurred in LUAD cases that developed off‐target resistance to 1st/2nd generation EGFRi and that this off‐target resistance mechanism was not overcome by improved oncogene targeting with the 3^rd^ generation EGFRi. This suggests that BM activation is a key determinant of TKI resistance in LUAD, representing a frequently activated off‐target mechanism of resistance that supersedes the presence of an actionable oncogenic driver in terms of response rates.

### 3.5 BM activation predicts poor response to immune checkpoint blockade

Previous studies have shown that high TMB and *TP53* alterations can limit the efficacy of chemotherapy and TKI treatments [58], which may in part explain the association between BM and TKI resistance. However, these genetic features have also been identified as favourable prognostic indicators for immune checkpoint blockade (ICB) [59]. Moreover, using RPPA data from TCGA cohort we found that PD‐L1 protein expression was significantly upregulated in BM‐high vs BM‐low LUAD samples (Fig. S4H). Therefore, three key biomarkers for favourable ICB response [60] were upregulated in BM‐high LUAD.

This suggested that while BM‐high LUAD tumours were refractory to treatments directly targeting the tumour cells, they may be more vulnerable to ICB. To test this hypothesis, we analysed BM activation in pre‐treatment biopsies from patients that received ICB monotherapy [39]. Contrary to expectations, we found BM‐high tumours had significantly reduced progression‐free survival rates (Fig. 4D). Further analysis of a second cohort [38] confirmed this finding, showing BM activation was significantly reduced in tumours that showed a major pathological response to combined ICB and chemotherapy treatment (Fig. 4E). We analysed scRNA‐seq data from post‐treatment LUAD samples in the Hu et al. dataset [38] to determine whether ICB treatment resulted in changes in BM activation as we had found following TKI treatment. This showed that only the ALV‐BM+ LUAD population was enriched in post‐treatment samples with no major pathological response to combined ICB and chemotherapy treatment. Notably (and unlike TKI resistant samples), the proportion of ALV‐BM‐ LUAD cells was unchanged (Fig. 4F).

In summary, BM activation was a key factor in promoting LUAD progression and resistance to current treatments identifying patients that, although positive for ICB response biomarkers, will likely fail to respond to this treatment.

### 3.6 BM activation in LUAD is associated with a basal‐like phenotype

**Fig. 5:**
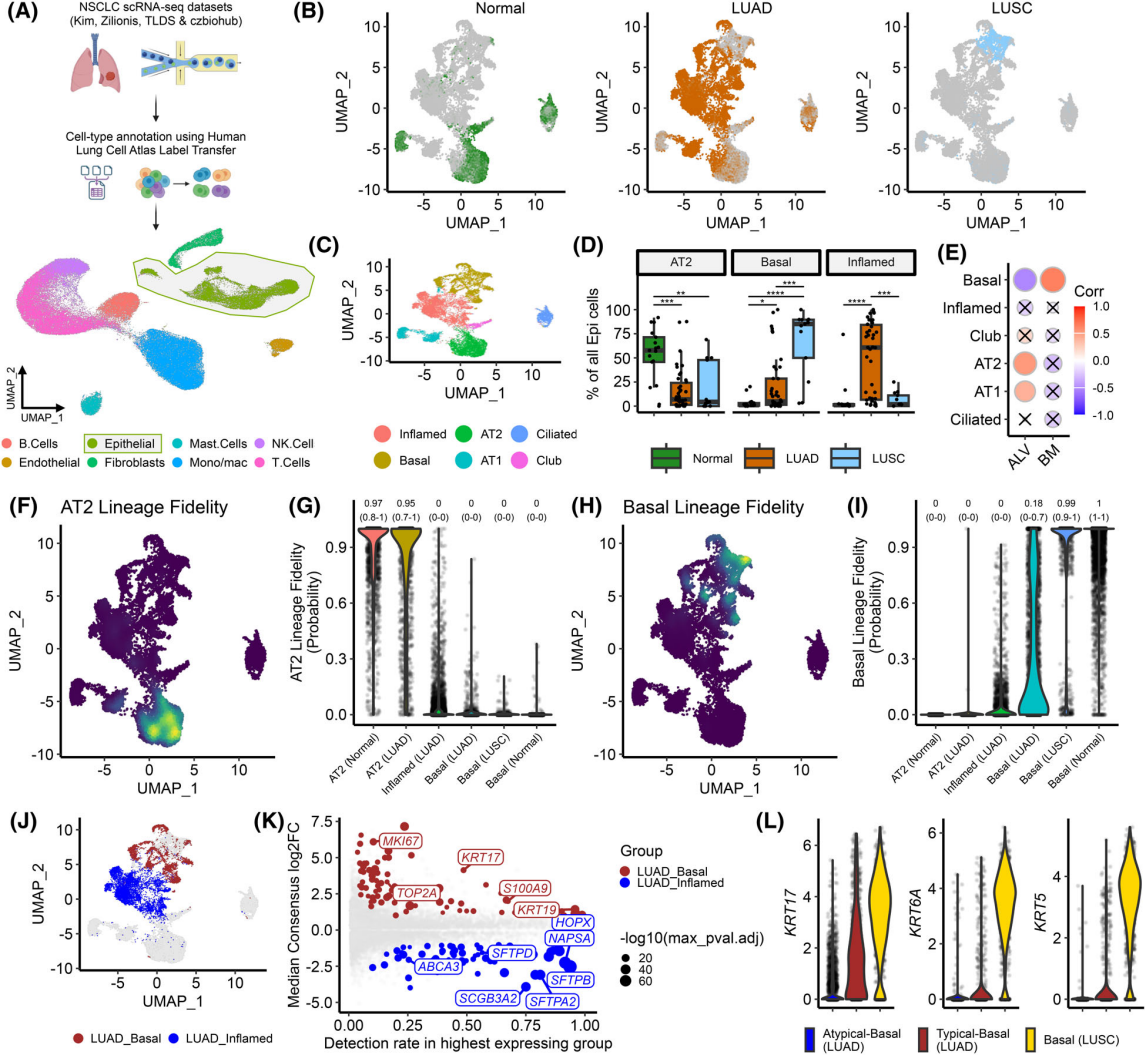
BM upregulation in LUAD is associated with acquisition of basal‐like features. (A) Schematic and UMAP showing cell type annotation from integrated scRNA‐seq analysis of 4 independent NSCLC cohorts ‘Kim’, ‘Czbiohub’, ‘Zilionis’ and ‘TLDS’, implementing label transfer analysis using the Human Lung Cell Atlas (HLCA) described by Travaglini et al. (B–E) NSCLC epithelial subpopulation analysis by scRNA‐seq (*n* = 16 621 epithelial cells from 72 samples): UMAP visualisation of cells isolated from LUAD, LUSC, and normal samples (B); UMAP visualisation of subpopulation clustering (C); and boxplots showing the relative abundance of each subpopulation across LUAD, LUSC and normal samples, asterisks represent FDR adjusted p‐values from pairwise Wilcoxon rank‐sum tests– non‐significant (*p* > 0.05) comparisons are not shown (D); Dot plot showing Pearson's correlation between subpopulation abundance and ssGSEA scores for ALV/BM—*p*‐values > 0.01 are crossed out (E). (F, G)—UMAP (F) and Boxplots (G) showing AT2 lineage fidelity scores. (H, I)—UMAP (H) and Boxplots (I) showing Basal lineage fidelity scores. Labels in G&I indicate median (IQR). (J) UMAP showing epithelial cells classified as LUAD_Inflamed and LUAD_Basal. (K) Scatter plot showing consensus differential expression analysis (across three independent scRNA‐seq datasets) comparing Inflamed and Basal subpopulations from LUAD samples, significance was determined by maximum adjusted *p*‐value < 0.05. (L) Violin plots showing expression levels for basal keratins in LUAD cells from the Basal cluster, grouped based on their Basal Lineage Fidelity (atypical‐basal < 0.5; typical‐basal > 0.5) or LUSC basal cells. BM—branching morphogenesis, NSCLC—non‐small cell lung cancer, LUAD—lung adenocarcinoma, LUSC—lung squamous cell carcinoma, AT2—alveolar type 2 cell, ssGSEA—single‐sample gene set enrichment analysis, UMAP—Uniform Manifold Approximation and Projection, *—*p* ≤ 0.05, **—*p* ≤ 0.01, ***—*p* ≤ 0.001, ****—*p* ≤ 0.0001.

To investigate ALV and BM activation in NSCLC further, we integrated four scRNA‐seq NSCLC datasets [41, 42, 43, 44]. We then performed cell type classification using a machine learning classifier, trained on the Human Lung Cell Atlas (HLCA) described by Travaglini et al. [45] (Fig. 5A). Cells classified as epithelial were extracted from each dataset, integrated to mitigate batch effects [47], and manually filtered to exclude doublets. This generated a dataset containing 16 621 epithelial cells from 72 samples: 69% LUAD (*n* = 42); 7.5% LUSC (*n* = 13) and 23.5% control (non‐neoplastic; *n* = 17). Transcriptomic variation between control, LUAD and LUSC epithelium was clear following UMAP visualisation (Fig. 5B). Consistent with the analyses presented above, pseudo bulk expression profiles for each sample showed that ALV and BM scores were significantly negatively correlated (*r* = −0.68, *p* = 4.1e‐09) (Fig. S5A).

Given that the epithelial compartment of adult lung tissue consists of multiple lineages, we investigated whether specific subpopulations were associated with BM activation. Unsupervised clustering identified 6 epithelial subpopulations, 5 of which represent well‐described lineages: ‘Basal’, ‘AT2’, ‘AT1’, ‘Ciliated’ and ‘Club’ (Fig. 5C and S5B).

The ‘Basal’ subpopulation was characterised by high molecular weight keratins (*KRT5/6B/10/14/15/16/17)* and other canonical markers (*e.g*. *DAPL1* and *TP63*), in addition to proliferation‐associated genes (*e.g*. *MKI67*, *STMN1*, *TUBB, TOP2A*) (Fig. S5B). This subpopulation was significantly enriched in cancer samples, particularly in LUSC (Fig. 5D). Notably, 82% of all LUSC cells were assigned to the ‘Basal’ cluster—consistent with previous studies describing basal cells as the cell‐of‐origin for LUSC tumours.

The ‘AT2’ subpopulation was identified by overexpression of canonical markers (*e.g. SFTPC, SFTPD, SFTPA1/2, ABCA3* and *NAPSA;* Fig. S5B). Despite AT2 cells being well described as the cell‐of‐origin for LUAD, this population was significantly less abundant in LUAD samples compared to control, suggesting a high degree of transcriptomic plasticity within LUAD epithelium (Fig. 5D).

‘AT1’ cells were identified by overexpression of canonical markers such as *AGER, CAV1* and *EMP2* (Fig. S5B). Interestingly, these cells formed two discrete groups in the UMAP projection (Fig. 5C). One subset was only present in LUAD samples (Fig. 5B) and expressed canonical AT1 marker genes at a lower level than the AT1 cells isolated from control tissue samples (Fig. S5B). It also expressed markers of AT2 cells, such as *NAPSA* and *SFTPD*, likely reflecting the mixed‐lineage hybrid state described in previous LUAD studies [5, 61].

Ciliated cells were identified by overexpression of *TPPP3, SNTN, CAPS, TSPAN1, CDHR3* (Fig. S5B); and Club cells by overexpression of *SCGB1A1, SCGB3A1* and *BPIFB1* (Fig. S5B). Both subpopulations were significantly downregulated in LUAD samples compared to control samples (Fig. S5C).

We named the sixth population ‘Inflamed’. It was marked by genes associated with prostaglandin and interferon signalling (*e.g. HPGD, IRF7*, *EREG*, *BST2*; Fig. S5B). This population was the most abundant phenotype in LUAD samples and significantly enriched in LUAD samples compared to control or LUSC samples (Fig. 5D). Notably, this population expressed high levels of *HOPX*, which is commonly used as an AT1 cell marker but has been shown to be expressed by multiple epithelial subpopulations in murine lungs [62] (Fig. S5D).

To determine whether the relative abundance of specific epithelial subpopulations was associated with developmental ALV/BM we performed a correlation analysis between the epithelial clusters and sample‐level ALV/BM scores (Fig. 5E). This identified a significant positive correlation for ‘AT1’ and ‘AT2’ clusters with ALV and showed the basal subpopulation to correlate with BM.

Cell‐of‐origin is a key determinant of NSCLC subtype [63, 64]. However, histological transformation can occur in response to therapy [65]. Given the varied cell‐of‐origin for LUAD and LUSC, we hypothesised that the mechanisms of BM activation would vary between subtypes: LUSC solely requiring the expansion of malignant basal cells; and LUAD requiring proliferative expansion and de‐differentiation from an AT2 to basal phenotype. To investigate this, we first confirmed that BM activation and basal cell abundance correlated after excluding LUSC samples (*r* = 0.64, *p* = 2.01e‐07, Fig. S5E). We then utilised the HLCA dataset as a reference to perform label transfer analysis from normal AT2 cells to the epithelial cells present in our dataset using the probability of these classifications as a measure of AT2 lineage fidelity (Fig. 5F). As expected, cells in our AT2 cluster (from both normal and LUAD tissues) had very high lineage fidelity (median > 0.95); whereas both inflamed and basal cells from LUAD tissues had almost ubiquitously lost AT2 lineage fidelity (Fig. 5F,G). We then performed a similar analysis of basal lineage fidelity (Fig. 5H). This showed that the Basal LUAD cluster had only limited fidelity to the basal lineage (median probability = 0.18), suggesting most of these cells have acquired an atypical basal‐like state rather than undergoing complete transdifferentiation to a basal cell phenotype (Fig. 5H,I). Notably, the Inflamed LUAD population had minimal basal lineage fidelity (median probability = 0; Fig. 5I).

To examine the phenotype of LUAD‐basal cells further, we performed differential gene expression analysis comparing LUAD cells from Inflamed and Basal clusters (Fig. 5J,K). This showed LUAD‐basal cells downregulated expression of alveolar and club cell marker genes (e.g. *SFTPD*, *SFTPB*, *SFTPA2*, *ABCA3*, *NAPSA*, *SCGB3A2*). LUAD‐basal cells upregulated *S100A9*; certain genes also highly expressed by LUSC basal cells (*e.g*. *KRT17*, *KRT19*); and proliferation‐associated genes (*e.g*. *MKI67, TOP2A*). However, canonical basal cell markers (e.g. *KRT14*, *KRT5*, *TP63 and DAPL1*) were only detected in a small proportion of LUAD‐basal cells and were not found to be significantly upregulated (Fig. S5F).

We then stratified LUAD‐basal cells into two groups based on the Basal Lineage Fidelity score (‘typical’ > 0.5 and ‘atypical’ < 0.5): 69% of LUAD‐basal cells were classified as atypical and 31% as typical basal cells. We found that the ‘typical’ LUAD‐basal cells expressed higher levels of canonical basal markers such as *KRT5, KRT6A* and *KRT17* (Fig. 5L), as well as lower levels of alveolar markers, including *SFTPB, NAPSA* and *ABCA3*, compared to the atypical LUAD‐basal cells (Fig. S5G).

To summarise, in LUAD tumours differentiated alveolar cells were lost and replaced predominantly by malignant cells with an inflamed or basal‐like phenotype. In cases with BM activation the malignant LUAD cells were characterised by an increased abundance of basal‐like cells. These cells upregulated genes highly expressed by LUSC, but to a lower level, suggesting that LUAD‐basal cells represented a distinct phenotype rather than a complete histological transformation.

### 3.7 BM activation is found in high grade LUAD tumours

**Fig. 6:**
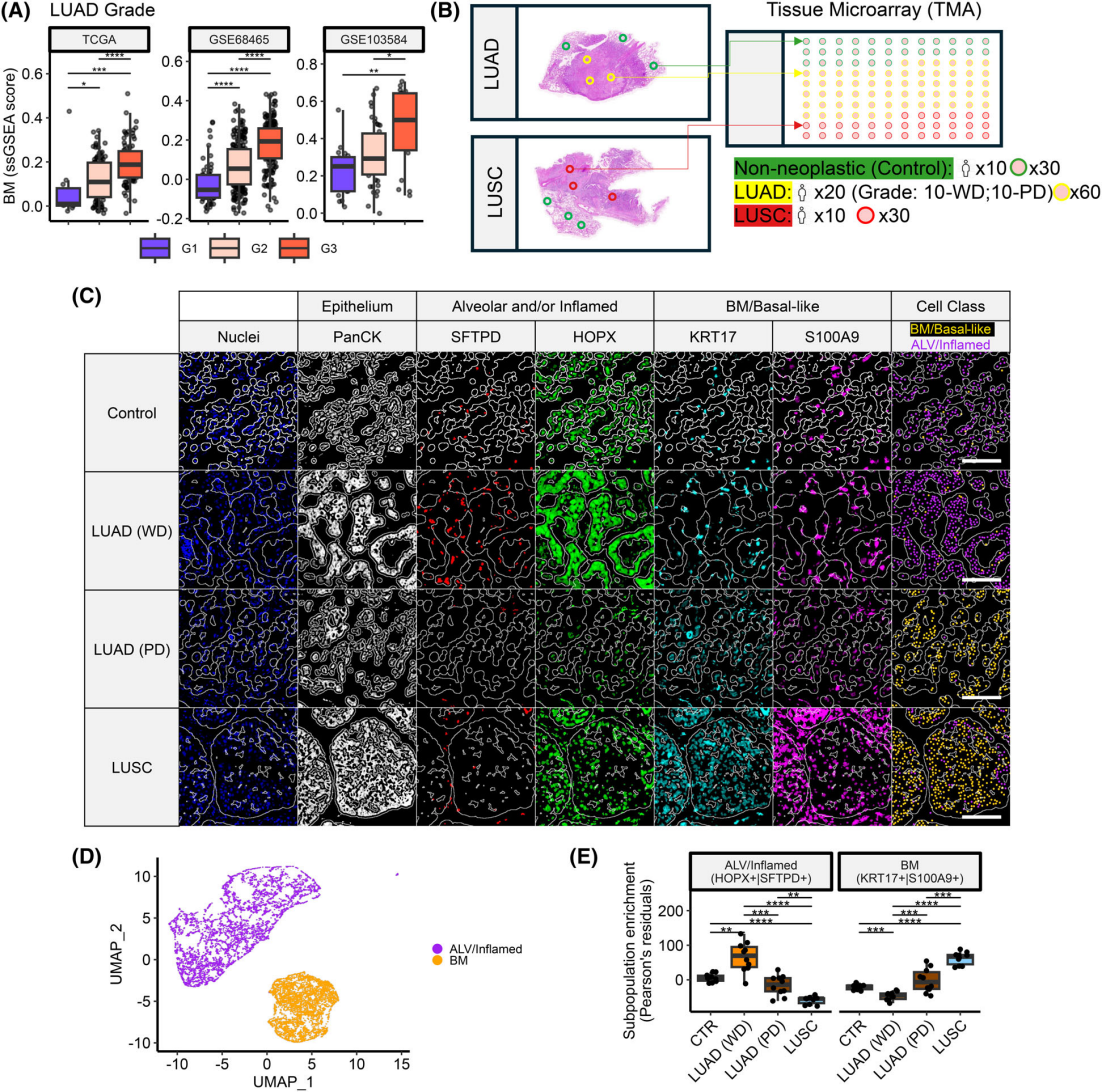
BM activation is found in high grade LUAD tumours. (A) Boxplots comparing BM ssGSEA scores across LUAD grade (G1—well differentiated, G2—moderately differentiated, G3—poorly differentiated) in 3 independent transcriptomics datasets (TCGA *n* = 187; GSE68465 *n* = 436; GSE103584 *n* = 80). (B) Graphical representation of tissue sampling from LUAD and LUSC blocks to form a tissue microarray (TMA) used for multiplexed immunohistochemistry (WD—well differentiated; PD—poorly differentiated [solid morphology]). (C) Representative images from multiplexed immunohistochemistry (mxIHC) analysis of NSCLC TMAs showing pseudo immunofluorescence (pseudoIF; blue staining represents nuclei) and cell classification from histo‐cytometry analysis (Cell class) of epithelial (PanCK+) cell expression of BM markers (KRT17, S100A9) and ALV/Inflamed markers (SFTPD, HOPX). White lines indicate the outline of Pan‐CK+ tissue regions. Scale bar represents 100 microns. (D) UMAP of histo‐cytometry measurements from mxIHC analysis showing HC class assignments; plot displays a randomly sampled 5% of epithelial cells to minimise over‐plotting. (E) Enrichment of Epi ALV/Inflamed and Epi‐BM subpopulations across NSCLC sample subtypes measured by mxIHC (*n* = 40). Asterisks represent P values calculated with pairwise Wilcoxon rank‐sum tests with FDR correction where multiple tests were performed; non‐significant (*p* > 0.05) comparisons are not shown (Fig A&E). ALV—alveogenesis, BM—branching morphogenesis, LUAD—lung adenocarcinoma, LUSC—lung squamous cell carcinoma, TCGA—The Cancer Genome Atlas, NSCLC—non‐small cell lung cancer, HC—histochemistry, FDR—false discovery rate, **—*p* ≤ 0.01, ***—*p* ≤ 0.001, ****—*p* ≤ 0.0001.

The association between BM activation and loss of alveolar differentiation prompted us to test whether ALV/BM signatures were linked to morphological grade. This showed that, across multiple cohorts, high grade (poorly differentiated) LUAD tumours have significantly higher BM scores and lower ALV scores than low grade tumours (Fig. 6A and S6A). In contrast, no significant differences were found in ALV/BM scores for LUSC tumours of different grades (Fig. S6B).

To examine the relationship between tumour morphology and the molecular profile of epithelial cells at the protein level, we utilised multiplexed immunohistochemistry (mxIHC) analysis of tissue microarrays (TMAs). For this analysis we curated a cohort of archival FFPE samples to incorporate control (non‐neoplastic) lung tissue; low grade (well differentiated [WD]) LUAD; high grade (poorly differentiated [PD] ‐ solid morphology) LUAD; and LUSC (10 samples per group, 3 cores per sample; Fig. 6B). MxIHC was performed using an antibody panel designed to identify epithelial cells (Pan‐Cytokeratin [PanCK]+) expressing BM markers (S100A9 and KRT17) or ALV/inflamed markers (SFTPD and HOPX) (Fig. 6C). In addition, MKI67 and SOX9 were examined as phenotypic markers of proliferation and developmental BM, respectively. UMAP analysis showed that the BM+ and ALV/inflamed+ subpopulations were clearly discriminated using this panel (Fig. 6D and Fig. S6C). Consistent with our transcriptomic analysis, BM+ cells were increased in LUSC compared to LUAD; and in poorly differentiated LUAD compared to well‐differentiated LUAD (Fig. 6E). Furthermore, MKI67 expression was significantly increased in BM+ cells compared to ALV/inflamed+ cells across all tumour sample subtypes (Fig. S6D); whereas SOX9 expression was similarly expressed in both ALV/inflamed+ and BM+ cells from well differentiated LUAD but significantly increased in BM+ compared to ALV/inflamed+ cells from poorly differentiated LUAD or LUSC (Fig. S6E).

### 3.8 Type I interferon promotes BM activation in TP53‐mutant LUAD

**Fig. 7:**
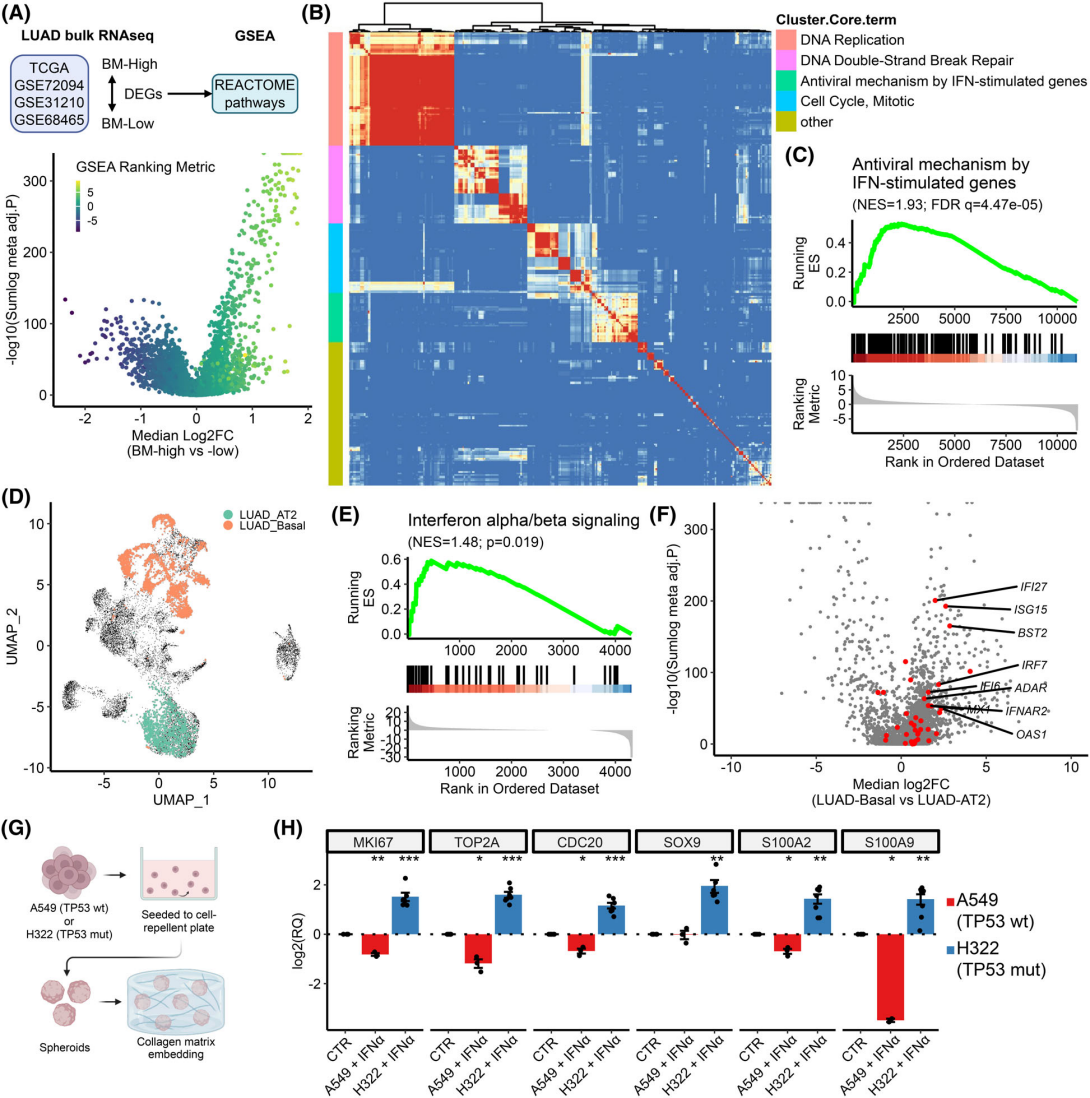
Type I interferon promotes BM activation in TP53‐mutant LUAD. (A–C) – Bulk transcriptome differential expression (DE) and REACTOME pathway gene set enrichment analysis (GSEA), comparing BM‐high and BM‐low samples performed on 4 independent LUAD datasets (TCGA, GSE72094, GSE68465, GSE31210): (A) Volcano plot showing DE analysis results; (B) Heatmap showing significantly (FDR‐*q* < 0.01) enriched pathways, clustered using Ward's method based on the overlap between leading edge genes; (C) GSEA plot for the ‘Antiviral mechanism by IFN‐stimulated genes’ (R‐HSA‐1169410) pathway, ES—Enrichment score, NES—normalised enrichment score. D–F—Single‐cell (sc)RNA‐seq DE and REACTOME pathway GSEA, meta‐analysis across 3 LUAD datasets: (D) UMAP showing epithelial cells from the integrated NSCLC dataset highlighting LUAD‐derived AT2 cells and LUAD‐derived basal cells; (E) GSEA plot for the ‘Interferon alpha/beta signalling’ pathway; (F) Volcano plot showing differentially expressed genes between LUAD‐derived AT2 cells and LUAD‐derived basal cells (from D), labels shown for genes associated with ‘Interferon alpha/beta signalling’ pathway (R‐HSA‐909733). (G, H)—*In vitro* analysis of 3D organotypic cultures using the H322 (TP53‐mutant LUAD cell line) or A549 (TP53 wild‐type [wt] cell line): (G) Schematic showing the culture setup; (H) Barplot showing mean log2(treatment vs. control relative quantity [RQ]) +/‐SEM, from qPCR analysis of BM‐associated genes in H322 spheroids treated with 1000 U/mL of IFNα for 72 h (*n* = 3 [A549] and 7 [H322] independent experiments); asterisks represent FDR adjusted p‐values from one‐sample t‐tests comparing log2(RQ) to 0. BM—branching morphogenesis, IFN—interferon, FDR—false discovery rate, SEM—standard error of mean, LUAD—lung adenocarcinoma, *—*p* ≤ 0.05, **—*p* ≤ 0.01, ***—*p* ≤ 0.001.

To further elucidate the mechanism of BM activation in LUAD we performed REACTOME pathway GSEA, ranking genes based on consensus upregulation in BM‐high compared to BM‐low LUAD across four bulk tissue datasets (Fig. 7A). This identified 320 significantly (FDR‐Q < 0.01) enriched terms, associated with 4 major clusters of related pathways (Fig. 7B). The core process associated with each of these clusters was DNA Replication (78 pathways); DNA Double‐Strand Break Repair (54 pathways); Antiviral mechanism by IFN‐stimulated genes (34 pathways); and Cell Cycle, Mitotic (48 pathways), respectively.

Given that interferon signalling has predominantly been associated with tumour suppression [66], the enrichment of the Antiviral mechanism by IFN‐stimulated genes pathway in these aggressive LUAD tumours was surprising (Fig. 7C; NES = 1.93, FDR *q* = 4.47e‐5). *ISG15* was among the top upregulated genes associated with this pathway, which is well described to be induced by IFN‐I signalling [67]. To investigate these pathways further and specifically in relation to tumour cell phenotypes we examined their expression in our scRNA‐seq dataset (Fig. 7D). This showed that both the Antiviral mechanism by IFN‐stimulated genes (R‐HSA‐1169410; NES = 1.47, *p* = 0.011; Fig. S7A) and Interferon alpha/beta signalling (R‐HSA‐909733; NES = 1.48, *p* = 0.019; Fig. 7E) REACTOME pathways were enriched among genes upregulated in LUAD‐basal cells compared to (well‐differentiated) LUAD‐AT2 cells (Fig. 7D–F); including IFN‐I receptors (*IFNAR1* and *IFNAR2*) and response genes (*e.g*. *ISG15*, *IFI27*, *BST2*, *IFI6*, *IFITM2* and *IFITM1*).

Previous studies have indicated that high levels of IFNα stimulation (> 10 kU/ml) are cytotoxic to NSCLC cells, whereas chronic low‐level signalling can be tumour promoting and cytoprotective [68, 69]. To test whether low‐level interferon signalling regulates BM activation and basal‐like transdifferentiation in LUAD cells, we analysed the response of H322 (a *TP53*‐mutant LUAD cell line) cells to sub‐cytotoxic IFNα stimulation (10, 100 and 1000 U/mL for 72 h) in 3D organotypic cultures (Fig. 7G). These cultures showed upregulation of *ISG15* in H322 spheroids treated with IFNα (Fig. S7B) and a dose dependent upregulation of markers for both the BM and Basal‐like phenotypes (*MKI67*, *TOP2A*, *CDC20, SOX9, S100A2*, *S100A9* and *KRT17*; Fig. S7C). Notably, IFNα induced upregulation of BM‐associated genes was only observed in 3D cultures, not when H322 cells were treated as a 2D monolayer (Fig. S7D).

Given our previous findings linking TP53 loss‐of‐function to BM activation and TP53's role downstream of IFN‐I [70], we hypothesised that these pro‐tumorigenic effects may be limited to TP53 mutant LUAD. To test this, we repeated the 3D organotypic culture experiment comparing the response of H322 to A549 (a TP53 wild‐type LUAD cell line). This showed that A549 cells had the opposite response to IFNα, resulting in downregulation of multiple BM markers (Fig. 7H).

In summary, we have shown that IFN‐I signalling in LUAD tumour cells harbouring TP53 mutations was involved in regulating the activation of BM and the basal‐like phenotype.

## 4 Discussion

We have demonstrated that developmental programmes (ALV and BM) frequently become dysregulated in NSCLC, with BM activation identifying aggressive LUAD that are resistant to multiple therapies. We found that BM activation in LUAD was associated with *TP53* pathway mutations, leading to AT2 cells losing alveolar lineage identity and acquiring a basal‐like state, which can be driven by IFN‐I signalling. These findings highlight an important mechanism of disease progression and therapy resistance in LUAD.

In LUSC, we found near ubiquitous upregulation of the BM programme and limited evidence for epithelial plasticity compared to LUAD. This may explain why these developmental programmes were less consequential in terms of LUSC patient survival rates. However, the scRNA‐seq datasets analysed consisted of relatively few LUSC samples compared to LUAD, which may have limited our ability to comprehensively characterise epithelial plasticity in this subtype. Future studies should consider expanding this analysis to a larger cohort of LUSC samples.

BM activation in LUAD was associated with resistance to and recurrence from multiple therapeutic strategies, including TKIs and ICB. TKI resistance is known to be in part driven by on‐target mutations, such as EGFR T790M mutations, but the mechanisms of resistance in other cases are poorly understood. Our results suggest that BM activation is a critical determinant of TKI resistance and likely underpins the association previously described between TP53 mutations and TKI resistance [71], providing an opportunity for refined biomarker development. Similarly, our results suggest that assessing BM activation could improve upon the current gold standard ICB response biomarkers (TMB and PDL1 expression [60]), which are known to be sub‐optimal. Furthermore, the consistent association of BM activation with poor survival and therapy resistance highlights this process as a fundamental mechanism of disease progression, presenting opportunities to identify novel therapeutic targets.

Loss of AT2 lineage fidelity has been described as an early event in KRAS driven LUAD [72]; representing a key factor in disease progression and TKI therapy resistance [42, 57]. We showed that BM activation involved concomitant loss of AT2 lineage fidelity and transdifferentiation to a basal‐like phenotype. Basal cell phenotypes have been linked to aggressive subtypes of breast, pancreatic and bladder cancers: associated with stemness [73], invasion [74], metastatic dissemination [75] and therapy resistance [76]. Notably, our analysis showed BM activation was strongly linked to TP53 pathway mutations; but reduced in cases with *KRAS* mutations and wild‐type *TP53*. During murine lung development, hyperactive Kras was shown to be sufficient to induce Sox9 expression, leading to extensive branching and suppressed alveolar differentiation [9]. In murine models of Kras‐driven LUAD, p53's tumour suppressive properties include restricting cellular plasticity and promoting AT1‐like differentiation [15]. Similarly, in bleomycin induced fibrosis, p53 activity is required for regeneration of AT1 cells from AT2 progenitors, with loss of p53 function causing AT2‐cells to stall in a transient state marked by *Krt19* expression [14]. This transient state shared many features with KRT5^−^KRT17^+^ basaloid cell state found in human IPF, consistent with the basal‐like phenotype we identified in human LUAD. Notably, p53 can act as a direct transcriptional repressor of Krt17 in rats, suggesting a link between the elevated expression of *KRT17* and loss of p53 in BM‐high LUAD tumours [77]. Based on this evidence, it is likely that LUAD tumours achieve loss of AT2 lineage fidelity via multiple means, with *KRAS* or *EGFR* mutations representing common examples of genetic drivers. However, acquisition of basal‐like features requires loss of p53 function. Furthermore, as p53 activity is known to limit stem cell renewal and promote differentiation, these basal‐like features are likely linked to cancer stemness [78]. Given our finding that ALV‐BM+ cells were the only epithelial population increased in ICB resistant LUAD cases, the acquisition of basal‐like features in addition to loss of lineage fidelity may be highly relevant in the context of immune evasion.

Finally, we have shown that IFN‐I signalling promotes BM activation in p53‐mutant LUAD. Although many studies have demonstrated IFN‐I has anti‐tumour properties, our results contribute to a growing body of literature describing a nuanced and context dependent role in both tumour progression and therapy resistance. For example, IFN‐I signalling has been shown to regulate adaptive resistance to EGFR inhibitors in LUAD [23]; initiate epigenetic reprogramming of breast cancer cells towards a more aggressive, stem‐like phenotype [22]; and sustained IFN‐I signalling has been shown to suppress anti‐tumour immunity in the context of immune checkpoint blockade and radiotherapy [79, 80].

Further evidence to support an interconnected role for IFN‐I signalling and TP53 loss‐of‐function can be found from studies investigating mechanisms of lung infection, where IFN‐I signalling has been shown to protect AT2 cells from inflammation‐induced cell death [81]. In this context, *TP53* is upregulated in response to IFN‐I stimulation, playing a role in antiviral defence by causing a reduction in proliferation of airway epithelial cells [70, 82]. The anti‐tumour properties of IFN‐I have also been shown to be p53 dependent [70], consistent with our observation that only H322 spheroids (*TP53* mutant cell line), but not A549 spheroids (*TP53* wild‐type cell line), upregulated BM‐associated genes in response to IFNα treatment. Our findings could provide a possible explanation as to why clinical trials of STING and TLR agonists (inducing type I interferon signalling) in the treatment of lung cancer have not met their primary endpoints, whilst showing promise in certain patient subpopulations [17, 18, 83].

## 5 Conclusions

We have shown that BM activation in LUAD tumours is a key mechanism of disease progression and therapy resistance with potential utility as a prognostic and predictive biomarker. We have demonstrated that BM activation is driven by malignant cells undergoing concomitant loss of AT2 lineage fidelity and acquisition of a basal‐like phenotype, which was enriched in both TKI and ICB recurrence. Importantly, we have also shown that acquisition of the basal‐like phenotype is dependent on p53 pathway mutations and can be driven by IFN‐I signalling. Therefore, careful patient selection is likely to be required for interferon‐stimulating therapies to yield clinical efficacy and therapeutic interventions that manipulate these developmental programmes or prevent transdifferentiation to a basal‐like state could lead to better clinical management of TP53 mutant LUAD patients.

## Conflict of interest

The authors declare no conflict of interest.

## Authors contribution

K. J. B. and C. J. H. contributed to conceptualisation, data curation, formal analysis, validation, visualisation and writing (original draft); K. J. B., S. G., N. Z., L.A., M. E., M. A. L., J. A. and S. P., S. J. C., A. A., E. C. S., C. H. O., G. J. T and C.J.H contributed to investigation and writing (review); S. J. C., A. A., E. C. S., C. H. O. and G. J. T. contributed to resources; C. H. O., G. J. T. and C. J. H. contributed to funding acquisition; G. J. T. and C. J. H. contributed to supervision and writing (editing).

## Supporting information


**File 1.** R Markdown HTML reports.

**Fig. S1.** Developmental Alveogenesis (ALV) and Branching Morphogenesis (BM) programmes are associated with transcriptomic variance in NSCLC.
**Fig. S2**. High expression of BM in LUAD predicts poor survival and is associated with frequent TP53 mutations.
**Fig. S3**. TP53 loss of function is required for BM activation.
**Fig. S4**. High expression of BM is associated with resistance to therapies.
**Fig. S5**. BM upregulation in LUAD is associated with acquisition of basal‐like features.
**Fig. S6**. BM in LUAD is associated with high grade tumours.
**Fig. S7**. Type I interferon promotes BM activation in TP53‐mutant LUAD.

**Table S1.** Alveogenesis and Branching morphogenesis gene signatures.

**Table S2.** IHC antibodies.

**Table S3.** qPCR primers.

**Table S4.** Source data.

**Table S5.** Public Dataset download information.

## Data Availability

All publicly available datasets analysed in this manuscript are detailed in Table S5. Source Data for all figures are provided in Table S4. All coding was performed using the publicly available packages cited, in R (v4.4.0). For further details, the R scripts used are available on Github (https://github.com/cjh‐lab/NSCLC_DevProgs) and R Markdown HTML reports are compiled in File 1. All datasets required to reproduce the analysis and figures have been published on Zenodo (10.5281/zenodo.16964653).
