# Supplementary material for: Developmental programmes drive cellular plasticity, disease progression and therapy resistance in lung adenocarcinoma
Source: Mol Oncol. 2026 May 27:10.1002/1878-0261.70263. Online ahead of print. doi: 10.1002/1878-0261.70263 (PMC13398952; doi:10.1002/1878-0261.70263)
Supplement: Supplementary file 1 — File 1. R Markdown HTML reports. [file MOL2-9999-0-s006.zip › Bienkowska_etal_MolOnc_Fig1.html]

Developmental programmes drive cellular plasticity, disease progression and therapy resistance in lung adenocarcinoma


# Developmental programmes drive cellular plasticity, disease progression and therapy resistance in lung adenocarcinoma

### Figure 1 - Developmental Alveogenesis (ALV) and Branching Morphogenesis (BM) programmes are associated with transcriptomic variance in NSCLC

#### Kamila J Bienkowska, Stephany Gallardo Y, Nur S Zainal, Leena Arora, Matthew Ellis, Maria-Antoinette Lopez, Judith Austine, Sai Pittla, Serena J Chee, Aiman Alzetani, Emily C Shaw, Christian H Ottensmeier, Gareth J Thomas, Christopher J Hanley

#### 2025-11-27

## Load packages

```
library(GSVA)
library(ggsci)
library(factoextra)
library(biomaRt)
library(clusterProfiler)
library(ggplot2)
library(ggpubr)
library(dplyr)
library(nichenetr)
library(AnnotationDbi)
library(org.Mm.eg.db)
library(mgcv)
library(gam)
library(stringr)
library(tidyr)
```

## Load Data

```
setwd(input_files)
load(file="NSCLC_traits_all.Rdata")
load(file="NSCLC_vsd_all.Rdata")
load(file="Alveogenesis_signature_modified.Rdata")
load(file="Morphogenesis_signature_modified.Rdata")
Dev.sigs_df <- readxl::read_xlsx("pnas.1311760110_sd01.xlsx")
load(file = "MicroarrayData.RData")
```

## Main Figures

Figure 1B

```
Dev.sigs_df$Developmental_DEGs <-
  ifelse(Dev.sigs_df$TargetID %in% Alveogenesis_sig , "ALV",
         ifelse(Dev.sigs_df$TargetID %in% Morphogenesis_sig, "BM", "Not_DE"))

Figure_1B <- Dev.sigs_df[order(Dev.sigs_df$`E19-E14(Log2)`), ] %>%
  ggplot(aes(y = `E19-E14(Log2)`, x = 1:nrow(Dev.sigs_df), colour = Developmental_DEGs)) +
  theme_pubr(base_size = 7) +
  theme(legend.position = c(0,1), legend.justification = c(-0.1,1)) +
  ggrastr::geom_point_rast(size = 0.1) +
  xlab("Gene Rank") +
  ylab("log2(FC E19 [ALV]- E14 [BM])")  +
  theme(legend.key.size = unit(2,"pt"), legend.background = element_blank(), legend.title = element_blank())

ggsave(Figure_1B, path = Plots_out, file = "Figure_1B.svg",
       width = 4, height = 4, units = "cm")
Figure_1B
```

Figure 1C&D

```
# changing gene symbols to mouse gene symbols and to ENSEMBLE 
Morphogenesis_mouse <- Morphogenesis_sig %>% convert_human_to_mouse_symbols()
Morphogenesis_mouse <- na.omit(Morphogenesis_mouse)
Morphogenesis_m_ENSEMBL <- AnnotationDbi::select(org.Mm.eg.db, keys=Morphogenesis_mouse, columns='ENSEMBL', keytype='SYMBOL')
Dev.sigs_df$mouse_symbol <- Dev.sigs_df$TargetID %>% convert_human_to_mouse_symbols()
dev_sig_mouse_gene_ENSEMBL <- AnnotationDbi::select(org.Mm.eg.db, keys=Dev.sigs_df$mouse_symbol, columns='ENSEMBL', keytype='SYMBOL')
Dev.sigs_df$mouse_ENSEMBL <- dev_sig_mouse_gene_ENSEMBL$ENSEMBL[match(Dev.sigs_df$mouse_symbol, dev_sig_mouse_gene_ENSEMBL$SYMBOL)]

# BM
ego <- enrichGO(gene          = Morphogenesis_m_ENSEMBL$ENSEMBL,
                universe      = Dev.sigs_df$mouse_ENSEMBL,
                OrgDb         = org.Mm.eg.db,
                ont           = "BP",
                pAdjustMethod = "fdr",
                readable      = TRUE,
                keyType = "ENSEMBL")

Figure_1C <- 
  ego@result %>%
  mutate(nlog10_FDRQ = -log10(qvalue),
         split.names = WGCNA::formatLabels(Description, maxCharPerLine = 30, maxLines = 2)) %>%
  arrange(Count) %>%
  slice_max(nlog10_FDRQ, n = 7, with_ties = F) %>%
  ggplot(aes(x = nlog10_FDRQ, y = reorder(split.names, nlog10_FDRQ), size = Count)) +
  theme_pubr(base_size = 7) +
  geom_point() +
  xlab("-log10(FDR-Q)") +
  theme(axis.title.y = element_blank(),legend.position = "right", legend.key.size = unit(2, "pt"), axis.text.y = element_text(size = 5)) +
  scale_size(range = c(0.1,2)) + 
  geom_vline(xintercept = -log10(0.01), linetype = "dotted") +
  expand_limits(x = 0)

ggsave(Figure_1C, path = Plots_out, file = "Figure_1C.svg",
       width = 7, height = 4, units = "cm")
Figure_1C
```

```
# same for ALV
Alveogenesis_mouse <- Alveogenesis_sig %>% convert_human_to_mouse_symbols()
Alveogenesis_mouse <- na.omit(Alveogenesis_mouse)
Alveogenesis_m_ENSEMBL <- AnnotationDbi::select(org.Mm.eg.db, keys=Alveogenesis_mouse, columns='ENSEMBL', keytype='SYMBOL')

ego2 <- enrichGO(gene          = Alveogenesis_m_ENSEMBL$ENSEMBL,
                 universe      = Dev.sigs_df$mouse_ENSEMBL,
                 OrgDb         = org.Mm.eg.db,
                 ont           = "BP",
                 pAdjustMethod = "fdr",
                 readable      = TRUE,
                 keyType = "ENSEMBL")

Figure_1D <- 
  ego2@result %>%
  mutate(nlog10_FDRQ = -log10(qvalue),
         split.names = WGCNA::formatLabels(Description, maxCharPerLine = 30, maxLines = 2)) %>%
  arrange(Count) %>%
  slice_max(nlog10_FDRQ, n = 7, with_ties = F) %>%
  ggplot(aes(x = nlog10_FDRQ, y = reorder(split.names, nlog10_FDRQ), size = Count)) +
  theme_pubr(base_size = 7) +
  geom_point() +
  xlab("-log10(FDR-Q)") +
  theme(axis.title.y = element_blank(),legend.position = "right", legend.key.size = unit(2, "pt"), axis.text.y = element_text(size = 5)) +
  scale_size(range = c(0.1,2)) + 
  geom_vline(xintercept = -log10(0.01), linetype = "dotted") +
  expand_limits(x = 0)

ggsave(Figure_1D, path = Plots_out, file = "Figure_1D.svg",
       width = 7, height = 4, units = "cm")
Figure_1D
```

Figure 1E

```
#Variable gene analysis####
Dev.sig_list <- list(
  ALV = Alveogenesis_sig,
  BM = Morphogenesis_sig
)

ensembl <- useEnsembl(biomart = "ensembl", 
                      dataset = "hsapiens_gene_ensembl", 
                      mirror = "useast")
proliferation.go <- getBM(attributes=c('hgnc_symbol', 'ensembl_gene_id', 'go_id'),
                          filters = 'go', values = c('GO:0008283', "GO:0007049", "GO:0051301", "GO:0006260"), mart = ensembl)

Dev.sig_list$BM_CC = Dev.sig_list$BM[Dev.sig_list$BM %in% proliferation.go$hgnc_symbol]
Dev.sig_list$BM_nonCC = Dev.sig_list$BM[!Dev.sig_list$BM %in% proliferation.go$hgnc_symbol]

NSCLC_vsd_geneStats <- data.frame(
  row.names = rownames(NSCLC_vsd_all), 
  mean = rowMeans(NSCLC_vsd_all),
  var = apply(NSCLC_vsd_all, 1, sd),
  ALV = factor(rownames(NSCLC_vsd_all) %in% Dev.sig_list$ALV),
  BM = factor(rownames(NSCLC_vsd_all) %in% Dev.sig_list$BM),
  BM_CC = factor(rownames(NSCLC_vsd_all) %in% Dev.sig_list$BM_CC),
  BM_nonCC = factor(rownames(NSCLC_vsd_all) %in% Dev.sig_list$BM_nonCC)
)


model <- gam(var ~ lo(mean), data = NSCLC_vsd_geneStats)
NSCLC_vsd_geneStats$Mean.var_resid <- model$residuals


Figure_1E <- NSCLC_vsd_geneStats %>%
  ggplot(aes(x = mean, y = var)) +
  theme_pubr(base_size = 7) +
  ggrastr::rasterise(geom_point(size = 0.1, colour = "grey70"), dpi = 300)  +
  stat_smooth() +
  geom_point(data = NSCLC_vsd_geneStats[NSCLC_vsd_geneStats$ALV == T |
                                          NSCLC_vsd_geneStats$BM == T, ],
             size = 0.5, colour = "red") +
  ylab("Expression variance (sd)") + xlab("Expression level (mean)")

ggsave(Figure_1E, path = Plots_out, file = "Figure_1E.svg",
       width = 4, height = 4, units = "cm")
Figure_1E
```

Figure 1F

```
PCA_allgenes <-  prcomp(t(NSCLC_vsd_all), scale. = F)
PCA_allgenes.varStats <- get_pca_var(PCA_allgenes)

#rownames(NSCLC_traits_all) <- NSCLC_traits_all$Row.names
Figure_1F <- fviz_pca_ind(PCA_allgenes, geom = "point", pointshape = 19, pointsize = 0.1,
                          habillage = NSCLC_traits_all[rownames(PCA_allgenes$x), "Subtype"],
                          label = "none", addEllipses = T, invisible = "quali", palette = c("forestgreen", "darkorange3", "skyblue1")) +
  theme_pubr(base_size = 7) +
  ggrastr::rasterise(geom_point(aes(colour = NSCLC_traits_all[rownames(PCA_allgenes$x), "Subtype"]), size = 0.1), dpi = 512) +
  theme(plot.title = element_blank(), legend.title = element_blank(), legend.key.size = unit(5, "pt")) 

ggsave(Figure_1F, path = Plots_out, file = "Figure_1F.svg",
       width = 4, height = 4, units = "cm")
Figure_1F
```

Figure 1G

```
NSCLC_vsd_geneStats$PC1.contribution <- PCA_allgenes.varStats$cos2[,1]

Figure_1G <- NSCLC_vsd_geneStats %>%
  ggplot(aes(log(PC1.contribution+1), after_stat(scaled), fill = NSCLC_vsd_geneStats$ALV == T |
               NSCLC_vsd_geneStats$BM == T)) +
  theme_pubr(base_size = 7) +
  geom_density(alpha = 0.6) +
  scale_fill_manual(name = "ALV/BM genes",
                values = c("TRUE" = "burlywood2", "FALSE" = "red")) +
  ylab("Scaled Density") + xlab("log(Contribution to PC1)") +
  theme(legend.position = c(1,1), legend.justification = c(1,1), legend.key.size = unit(5,"pt")) 

ggsave(Figure_1G, path = Plots_out, file = "Figure_1G.svg",
       width = 4, height = 4, units = "cm")
Figure_1G
```

Figure 1H

```
ssGSEA_Dev_TCGA <- ssgseaParam(NSCLC_vsd_all, Dev.sig_list)
gsva.es <- gsva(ssGSEA_Dev_TCGA, verbose = T)

NSCLC_traits_all <- merge(NSCLC_traits_all, t(gsva.es), by = 0)
rownames(NSCLC_traits_all) <- NSCLC_traits_all$Row.names
NSCLC_traits_all <- NSCLC_traits_all[,-1]

Figure_1H <- NSCLC_traits_all %>%
  ggplot(aes(y = ALV, x = BM,
             colour = Subtype)) +
  theme_pubr(base_size = 7) +
  theme(legend.title = element_blank(), legend.key.size = unit(2,"pt"),
        legend.position = c(0,0), legend.justification = c(0,0),
        legend.background = element_blank())+
  geom_point(aes(colour = Subtype), size = 0.1) +
  stat_smooth(method = "lm", colour = "grey30") +
  scale_color_manual(values=c("forestgreen", "darkorange3","skyblue1")) +
  guides(colour = guide_legend(override.aes = list(size = 1))) + 
  stat_cor(
    aes(label = paste(..r.label..,..p.label.., sep = "~`,`~")), colour = "black", size = 2, label.x = Inf, label.y = Inf, hjust = 1, vjust = 1,
    method = "spearman") +
  xlab("BM (ssGSEA score)") + ylab("ALV (ssGSEA score)")

ggsave(Figure_1H, path = Plots_out, file = "Figure_1H.svg",
       width = 4, height = 4, units = "cm")
Figure_1H
```

Figure 1 I&J

```
NSCLC_traits_all$Subtype <- factor(NSCLC_traits_all$Subtype,
                                   levels = c("Normal", "LUAD", "LUSC"))


Figure_1Ii <- NSCLC_traits_all %>%
  ggplot(aes(x = Subtype, y = ALV, fill = Subtype)) +
  theme_pubr(base_size = 7) +
  geom_jitter(alpha = 0.5, size = 0.1, width = 0.2) +
  geom_boxplot(outlier.shape = NA) +  ggpubr::geom_pwc(method = "wilcox_test", p.adjust.method = "fdr", label = "p.adj.signif", step.increase = 0.1, 
                   label.size = 2, tip.length = 0, vjust = 0.5) + ylab("ALV\n(ssGSEA score)") +
  scale_fill_manual(values = c("forestgreen", "darkorange3", "skyblue1")) +
  theme(axis.title.x = element_blank(), axis.text.x = element_blank())

Figure_1Iii <- NSCLC_traits_all %>%
  ggplot(aes(x = Subtype, y = BM, fill = Subtype)) +
  theme_pubr(base_size = 7) +
  geom_jitter(alpha = 0.5, size = 0.1, width = 0.2) +
  geom_boxplot(outlier.shape = NA) +  ggpubr::geom_pwc(method = "wilcox_test", p.adjust.method = "fdr", label = "p.adj.signif", step.increase = 0.1, 
                   label.size = 2, tip.length = 0, vjust = 0.5) + ylab("BM\n(ssGSEA score)") +
  scale_fill_manual(values = c("forestgreen", "darkorange3", "skyblue1")) +
  theme(axis.title.x = element_blank(), axis.text.x = element_blank())

Figure_1Ji <- NSCLC_traits_all %>%
  ggplot(aes(x = Subtype, y = BM_CC, fill = Subtype)) +
  theme_pubr(base_size = 7) +
  geom_jitter(alpha = 0.5, size = 0.1, width = 0.2) +
  geom_boxplot(outlier.shape = NA) +  ggpubr::geom_pwc(method = "wilcox_test", p.adjust.method = "fdr", label = "p.adj.signif", step.increase = 0.1, 
                   label.size = 2, tip.length = 0, vjust = 0.5) + ylab("BM_CC\n(ssGSEA score)") +
  scale_fill_manual(values = c("forestgreen", "darkorange3", "skyblue1")) +
  theme(axis.title.x = element_blank(), axis.text.x = element_blank())

Figure_1Jii <- NSCLC_traits_all %>%
  ggplot(aes(x = Subtype, y = BM_nonCC, fill = Subtype)) +
  theme_pubr(base_size = 7) +
  geom_jitter(alpha = 0.5, size = 0.1, width = 0.2) +
  geom_boxplot(outlier.shape = NA) +
  ggpubr::geom_pwc(method = "wilcox_test", p.adjust.method = "fdr", label = "p.adj.signif", step.increase = 0.1, 
                   label.size = 2, tip.length = 0, vjust = 0.5) + ylab("BM_nonCC\n(ssGSEA score)") +
  scale_fill_manual(values = c("forestgreen", "darkorange3", "skyblue1")) +
  theme(axis.title.x = element_blank(), axis.text.x = element_blank())


Figure_1I <- ggarrange(Figure_1Ii,Figure_1Iii, ncol = 2, common.legend = TRUE, legend = "bottom")
Figure_1I <- annotate_figure(Figure_1I, top = text_grob("ALV and BM in NSCLC (TCGA)", size = 9))
ggsave(Figure_1I, path = Plots_out, file = "Figure_1I.svg",
       width = 7, height = 4, units = "cm")
Figure_1I
```

```
Figure_1J <- ggarrange(Figure_1Ji,Figure_1Jii, ncol = 2, common.legend = TRUE, legend = "bottom")
Figure_1J <- annotate_figure(Figure_1J, top = text_grob("Cell-cycle and branching in NSCLC (TCGA)", size = 9))
ggsave(Figure_1J, path = Plots_out, file = "Figure_1J.svg",
       width = 7, height = 4, units = "cm")
Figure_1J
```

## Supplementary figures

Figure S1A and S1B

```
Figure_S1A <- fviz_pca_var(PCA_allgenes, select.var = list(name = Dev.sig_list$ALV), label = "none") +
  theme_pubr(base_size = 7) + 
  ggtitle("ALV genes") + theme(plot.title = element_text(size = 14)) 
Figure_S1B <- fviz_pca_var(PCA_allgenes, select.var = list(name = Dev.sig_list$BM), label = "none") +
  theme_pubr(base_size = 7) +
  ggtitle("BM genes") + theme(plot.title = element_text(size = 14)) 

Figure_S1A+Figure_S1B
```

Figure S1C

```
# LCMD dataset
GSE31552 <- GEOquery::getGEO('GSE31552',GSEMatrix=TRUE)
GSE31552_traits <- pData(GSE31552$GSE31552_series_matrix.txt.gz)

GSE31552_LCM.samples <- GSE31552$GSE31552_series_matrix.txt.gz[, pData(GSE31552$GSE31552_series_matrix.txt.gz)$source_name_ch1 == "LCM Samples"]

GSE31552_collapseRows <- WGCNA::collapseRows(exprs(GSE31552_LCM.samples),
                                                     rowGroup = fData(GSE31552_LCM.samples)$gene_assignment,
                                                     rowID = fData(GSE31552_LCM.samples)$ID)
GSE31552_LCM.samples_filtered <- GSE31552_LCM.samples[GSE31552_collapseRows$selectedRow, ]
fData(GSE31552_LCM.samples_filtered)$HGNC_symbol <- str_split_fixed(fData(GSE31552_LCM.samples_filtered)$gene_assignment,
                                                                    " // ",
                                                                    3)[,2]

GSE31552_ssGSEA.input <- exprs(GSE31552_LCM.samples_filtered)[
  fData(GSE31552_LCM.samples_filtered)$category == "main" &
    !duplicated(fData(GSE31552_LCM.samples_filtered)$HGNC_symbol),
]
rownames(GSE31552_ssGSEA.input) <- fData(GSE31552_LCM.samples_filtered)$HGNC_symbol[
  match(rownames(GSE31552_ssGSEA.input), fData(GSE31552_LCM.samples_filtered)$ID)
]

# ssGSEA scores
ssGSEA_Epi.LCM_samples <- ssgseaParam(GSE31552_ssGSEA.input, Dev.sig_list)
ssGSEA_Epi.LCM_samples <- gsva(ssGSEA_Epi.LCM_samples, verbose = T) 


GSE31552_LCM.samples_traits_all <- merge(pData(GSE31552_LCM.samples), t(ssGSEA_Epi.LCM_samples), by = 0)

GSE31552_LCM.samples_traits_all$Subtype <-
  ifelse(GSE31552_LCM.samples_traits_all$`disease state:ch1` == "Non-Tumor", "NTa",
         ifelse(GSE31552_LCM.samples_traits_all$`disease state:ch1` == "Non-Tumor Bronchy", "NTb",
                ifelse(GSE31552_LCM.samples_traits_all$`disease state:ch1` == "Tumor" &
                         GSE31552_LCM.samples_traits_all$`cell type:ch1` == "Adeno carcinoma", "LUAD", "LUSC")))
# table(GSE31552_LCM.samples_traits_all$Subtype,GSE31552_LCM.samples_traits_all$`disease state:ch1`)
# table(GSE31552_LCM.samples_traits_all$Subtype)
rownames(GSE31552_LCM.samples_traits_all) <- GSE31552_LCM.samples_traits_all$Row.names

GSE31552_LCM.samples_traits_all$Subtype <- factor(
  GSE31552_LCM.samples_traits_all$Subtype,
  levels = c("NTa", "LUAD", "NTb",  "LUSC"))
  
GSE31552_filtered_geneStats <- data.frame(
  row.names = make.unique(rownames(GSE31552_ssGSEA.input)), 
  mean = rowMeans(GSE31552_ssGSEA.input),
  var = apply(GSE31552_ssGSEA.input, 1, sd),
  ALV = factor(rownames(GSE31552_ssGSEA.input) %in% Dev.sig_list$ALV),
  BM = factor(rownames(GSE31552_ssGSEA.input) %in% Dev.sig_list$BM),
  BM_CC = factor(rownames(GSE31552_ssGSEA.input) %in% Dev.sig_list$BM_CC),
  BM_nonCC = factor(rownames(GSE31552_ssGSEA.input) %in% Dev.sig_list$BM_nonCC)
)

model2 <- gam(var ~ lo(mean), data = GSE31552_filtered_geneStats)
GSE31552_filtered_geneStats$Mean.var_resid <- model2$residuals

Figure_S1C <- GSE31552_filtered_geneStats %>%
  ggplot(aes(x = mean, y = var)) +
  theme_pubr(base_size = 7) +
  geom_point(size = 0.1, colour = "grey70")  +
  stat_smooth() +
  geom_point(data = GSE31552_filtered_geneStats[GSE31552_filtered_geneStats$ALV == T |
                                                  GSE31552_filtered_geneStats$BM == T, ],
             size = 1, colour = "red") + ggtitle("Variable expression of developmental \n genes in LCMD") +
  theme(plot.title = element_text(size = 14)) + ylab("variance")

Figure_S1C
```

Figure S1D

```
PCA_allgenes <-  prcomp(t(GSE31552_ssGSEA.input), scale. = F)
PCA_allgenes.varStats <- get_pca_var(PCA_allgenes)

Figure_S1D <- fviz_pca_ind(PCA_allgenes, habillage = GSE31552_LCM.samples_traits_all[rownames(PCA_allgenes$x), "Subtype"],
                     label = "none", addEllipses = T, invisible = "quali", palette = c("forestgreen", "darkorange3", "gold", "skyblue1")) +
  theme_pubr(base_size = 7) + ggtitle("LCMD - PCA analysis") + theme(plot.title = element_text(size = 14)) 

Figure_S1D
```

Figure S1E

```
GSE31552_filtered_geneStats$PCA.1dim.contribution <- PCA_allgenes.varStats$cos2[,1]
Figure_S1E <- GSE31552_filtered_geneStats %>%
  ggplot(aes(log(PCA.1dim.contribution+1), after_stat(scaled), fill = GSE31552_filtered_geneStats$ALV == T |
               GSE31552_filtered_geneStats$BM == T)) +
  theme_pubr(base_size = 7) +
  geom_density(alpha = 0.6) +
  scale_fill_manual(name = "ALV/BM genes",
                    values = c("TRUE" = "burlywood2", "FALSE" = "red")) +
  ylab("Scaled Density") + xlab("log(Contribution to PC 1)") +
  theme(legend.position = c(1,1), legend.justification = c(1,1)) +
  theme(legend.title = element_text(size=14),
        legend.text = element_text(size=12))

Figure_S1E
```

Figure S1F and S1G

```
Figure_S1F <- fviz_pca_var(PCA_allgenes, select.var = list(name = Dev.sig_list$ALV), label = "none") +
  theme_pubr(base_size = 7) + 
  ggtitle("ALV genes - LCMD") + theme(plot.title = element_text(size = 14)) 
Figure_S1G <- fviz_pca_var(PCA_allgenes, select.var = list(name = Dev.sig_list$BM), label = "none") +
  theme_pubr(base_size = 7) +
  ggtitle("BM genes - LCMD") + theme(plot.title = element_text(size = 14)) 

Figure_S1F+Figure_S1G
```

Figure S1H

```
Figure_S1H <- as.data.frame(GSE31552_LCM.samples_traits_all) %>%
  ggplot(aes(y = ALV, x = BM), colour = Subbtype) +
  theme_pubr(base_size = 7) +
  theme(legend.title = element_blank(), legend.key.height = unit(1,"pt"),
        legend.key.width = unit(1,"pt"),
        legend.position = c(0,0), legend.justification = c(0,0),
        legend.background = element_blank())+
  geom_point(aes(colour = Subtype))  +
  stat_smooth(method = "lm", colour = "grey30") +
  scale_color_manual(values=c("forestgreen", "darkorange3","gold", "skyblue1")) +
  guides(colour = guide_legend(override.aes = list(size = 1))) + 
  ggtitle("LCMD - ALV/BM correlation") +
  stat_cor(
    aes(label = paste(..r.label..,..p.label.., sep = "~`,`~")), colour = "black", size = 4, label.x = Inf, label.y = Inf, hjust = 1, vjust = 1,
    method = "spearman") +
  theme(axis.title.x = element_text(size=12),
        axis.title.y = element_text(size = 12),
        legend.text = element_text(size=12),
        plot.title = element_text(size = 14)) + scale_x_continuous(breaks=c(0, 0.2, 0.4)) +xlab("BM (ssGSEA score)") +ylab("ALV (ssGSEA score)")

Figure_S1H
```

Figure S1I and S1J

```
Figure_S1Ii <- GSE31552_LCM.samples_traits_all %>%
  ggplot(aes(x = Subtype, y = ALV, fill = Subtype)) +
  theme_pubr(base_size = 7) +
  geom_jitter(alpha = 0.5, size = 0.1, width = 0.2) +
  geom_boxplot(outlier.shape = NA) +
  ggpubr::geom_pwc(method = "wilcox_test", p.adjust.method = "fdr", label = "p.adj.signif", step.increase = 0.1, label.size = 2, tip.length = 0, vjust = 0.5, hide.ns = T) +
  ylab("ALV\n(ssGSEA score)") +
  scale_fill_manual(values = c("forestgreen", "darkorange3",  "gold", "skyblue1")) +
  theme(axis.title.x = element_blank(), axis.text.x = element_blank())#, legend.key.height = unit(4,"pt"), legend.key.width = unit(4, "pt"))

Figure_S1Iii <- GSE31552_LCM.samples_traits_all %>%
  ggplot(aes(x = Subtype, y = BM, fill = Subtype)) +
  theme_pubr(base_size = 7) +
  geom_jitter(alpha = 0.5, size = 0.1, width = 0.2) +
  geom_boxplot(outlier.shape = NA) +
  ggpubr::geom_pwc(method = "wilcox_test", p.adjust.method = "fdr", label = "p.adj.signif", step.increase = 0.1, label.size = 2, tip.length = 0, vjust = 0.5, hide.ns = T) +
  ylab("BM\n(ssGSEA score)") +
  scale_fill_manual(values = c("forestgreen", "darkorange3",  "gold", "skyblue1")) +
  theme(axis.title.x = element_blank(), axis.text.x = element_blank())#, legend.key.height = unit(4,"pt"), legend.key.width = unit(4, "pt"))

Figure_S1Ji <- GSE31552_LCM.samples_traits_all %>%
  ggplot(aes(x = Subtype, y = BM_CC, fill = Subtype)) +
  theme_pubr(base_size = 7) +
  geom_jitter(alpha = 0.5, size = 0.1, width = 0.2) +
  geom_boxplot(outlier.shape = NA) +
  ggpubr::geom_pwc(method = "wilcox_test", p.adjust.method = "fdr", label = "p.adj.signif", step.increase = 0.1, label.size = 2, tip.length = 0, vjust = 0.5, hide.ns = T) +
  ylab("BM_CC\n(ssGSEA score)") +
  scale_fill_manual(values = c("forestgreen", "darkorange3",  "gold", "skyblue1")) +
  theme(axis.title.x = element_blank(), axis.text.x = element_blank())#, legend.key.height = unit(4,"pt"), legend.key.width = unit(4, "pt"))

Figure_S1Jii <- GSE31552_LCM.samples_traits_all %>%
  ggplot(aes(x = Subtype, y = BM_nonCC, fill = Subtype)) +
  theme_pubr(base_size = 7) +
  geom_jitter(alpha = 0.5, size = 0.1, width = 0.2) +
  geom_boxplot(outlier.shape = NA) +
  ggpubr::geom_pwc(method = "wilcox_test", p.adjust.method = "fdr", label = "p.adj.signif", step.increase = 0.1, label.size = 2, tip.length = 0, vjust = 0.5, hide.ns = T) +
  ylab("BM_nonCC\n(ssGSEA score)") +
  scale_fill_manual(values = c("forestgreen", "darkorange3",  "gold", "skyblue1")) +
  theme(axis.title.x = element_blank(), axis.text.x = element_blank())#, legend.key.height = unit(4,"pt"), legend.key.width = unit(4, "pt"))

Figure_S1I <- ggarrange(Figure_S1Ii,Figure_S1Iii, ncol = 2, common.legend = TRUE, legend = "bottom")
Figure_S1I <- annotate_figure(Figure_S1I, top = text_grob("ALV and BM (GSE31552 - LCMD)", size = 8))
ggsave(Figure_S1I, path = Plots_out, file = "Figure_S1I.svg",
       width = 7, height = 4, units = "cm")
Figure_S1I
```

```
Figure_S1J <- ggarrange(Figure_S1Ji, Figure_S1Jii, ncol = 2, common.legend = TRUE, legend = "bottom")
Figure_S1J <- annotate_figure(Figure_S1J, top = text_grob("Cell-cycle and branching (GSE31552 - LCMD)", size = 8))
ggsave(Figure_S1J, path = Plots_out, file = "Figure_S1J.svg",
       width = 7, height = 4, units = "cm")
Figure_S1J
```

Microarray data Figure S1k

```
#GSE72094 - LUAD
ssGSEA_Dev_GSE72094 <- GSVA::ssgseaParam(expr=GSE72094_matrix_filtered[,],
                                  Dev.sig_list)

ssGSEA_Dev_GSE72094 <- gsva(ssGSEA_Dev_GSE72094, verbose = T)

GSE72094_GEO_traits <- merge(GSE72094_GEO_traits, t(ssGSEA_Dev_GSE72094), by = 0)
rownames(GSE72094_GEO_traits) <- GSE72094_GEO_traits$Row.names
GSE72094_GEO_traits <- GSE72094_GEO_traits[,-1]
GSE72094_GEO_traits$Subtype <- "LUAD"

GSE72094_neg_cor <- GSE72094_GEO_traits %>%
  ggplot(aes(y = ALV, x = BM, colour = Subtype)) +
  theme_pubr(base_size = 7) +
  theme(legend.title = element_blank(), legend.key.height = unit(1,"pt"),
        legend.key.width = unit(1,"pt"),
        legend.position = c(0,0), legend.justification = c(0,0),
        legend.background = element_blank())+
  geom_point(size = 0.5) +
  stat_smooth(method = "lm", colour = "grey30") +
  scale_color_manual(values = c("LUAD" = "darkorange3")) +
  guides(colour = guide_legend(override.aes = list(size = 1))) +
  ggtitle("GSE72094") + stat_cor(
    aes(label = paste(..r.label..,..p.label.., sep = "~`,`~")), colour = "black", size = 4, label.x = Inf, label.y = Inf, hjust = 1, vjust = 1,
    method = "spearman") +
  theme(axis.title.x = element_text(size=12),
        axis.title.y = element_text(size = 12),
        legend.text = element_text(size=12),
        plot.title = element_text(size = 14)) +xlab("BM (ssGSEA score)") +ylab("ALV (ssGSEA score)")


#Okayama - LUAD

ssGSEA_Dev_Okayama <- GSVA::ssgseaParam(expr=Okayama_matrix_filtered[,],
                                 Dev.sig_list)
ssGSEA_Dev_Okayama <- gsva(ssGSEA_Dev_Okayama, verbose = T)

Okayama_GEO_traits2 <- merge(Okayama_GEO_traits2, t(ssGSEA_Dev_Okayama), by = 0)
rownames(Okayama_GEO_traits2) <- Okayama_GEO_traits2$Row.names
Okayama_GEO_traits2 <- Okayama_GEO_traits2[,-1]
Okayama_GEO_traits2$Subtype <- "LUAD"

Okayama_neg_cor <- Okayama_GEO_traits2 %>%
  ggplot(aes(y = ALV, x = BM, colour = Subtype)) +
 theme_pubr(base_size = 7) +
  theme(legend.title = element_blank(), legend.key.height = unit(1,"pt"),
        legend.key.width = unit(1,"pt"),
        legend.position = c(0,0), legend.justification = c(0,0),
        legend.background = element_blank())+
  geom_point(size = 0.5) +
  stat_smooth(method = "lm", colour = "grey30") +
  scale_color_manual(values = c("LUAD" = "darkorange3")) +
  guides(colour = guide_legend(override.aes = list(size = 1))) +
  ggtitle("GSE31210") + stat_cor(
    aes(label = paste(..r.label..,..p.label.., sep = "~`,`~")), colour = "black", size = 4, label.x = Inf, label.y = Inf, hjust = 1, vjust = 1,
    method = "spearman") +
  theme(axis.title.x = element_text(size=12),
        axis.title.y = element_text(size = 12),
        legend.text = element_text(size=12),
        plot.title = element_text(size = 14)) +xlab("BM (ssGSEA score)") +ylab("ALV (ssGSEA score)")

#Shedden - LUAD
ssGSEA_Dev_Shedden <- GSVA::ssgseaParam(expr=Shedden_matrix_filtered[,],
                                 Dev.sig_list)
ssGSEA_Dev_Shedden <- GSVA::gsva(ssGSEA_Dev_Shedden, verbose = T)

Shedden_GEO_traits <- merge(Shedden_GEO_traits, t(ssGSEA_Dev_Shedden), by = 0)
rownames(Shedden_GEO_traits) <- Shedden_GEO_traits$Row.names
Shedden_GEO_traits <- Shedden_GEO_traits[,-1]
Shedden_GEO_traits$Subtype <- "LUAD"


Shedden_neg_cor <- Shedden_GEO_traits %>%
  ggplot(aes(y = ALV, x = BM, colour = Subtype)) +
  theme_pubr(base_size = 7) +
  theme(legend.title = element_blank(), legend.key.height = unit(1,"pt"),
        legend.key.width = unit(1,"pt"),
        legend.position = c(0,0), legend.justification = c(0,0),
        legend.background = element_blank())+
  geom_point(size = 0.5) +
  stat_smooth(method = "lm", colour = "grey30") +
  scale_color_manual(values = c("LUAD" = "darkorange3")) +
  guides(colour = guide_legend(override.aes = list(size = 1))) +
  ggtitle("GSE68465") + stat_cor(
    aes(label = paste(..r.label..,..p.label.., sep = "~`,`~")), colour = "black", size = 4, label.x = Inf, label.y = Inf, hjust = 1, vjust = 1,
    method = "spearman") +
  theme(axis.title.x = element_text(size=12),
        axis.title.y = element_text(size = 12),
        legend.text = element_text(size=12),
        plot.title = element_text(size = 14)) +xlab("BM (ssGSEA score)") +ylab("ALV (ssGSEA score)") 

#
#LUSC
#GSE157009
ssGSEA_Dev_GSE157009 <- GSVA::ssgseaParam(expr=GSE157009_matrix_filtered[,],
                                   Dev.sig_list)

ssGSEA_Dev_GSE157009 <- gsva(ssGSEA_Dev_GSE157009, verbose = T)

GSE157009_GEO_traits <- merge(GSE157009_GEO_traits, t(ssGSEA_Dev_GSE157009), by = 0)
rownames(GSE157009_GEO_traits) <- GSE157009_GEO_traits$Row.names
GSE157009_GEO_traits <- GSE157009_GEO_traits[,-1]
GSE157009_GEO_traits$Subtype <- "LUSC"


GSE157009_neg_cor <- GSE157009_GEO_traits %>%
  ggplot(aes(y = ALV, x = BM, colour = Subtype)) +
  theme_pubr(base_size = 7) +
  theme(legend.title = element_blank(), legend.key.height = unit(1,"pt"),
        legend.key.width = unit(1,"pt"),
        legend.position = c(0,0), legend.justification = c(0,0),
        legend.background = element_blank())+
  geom_point(size = 0.5) +
  stat_smooth(method = "lm", colour = "grey30") +
  scale_color_manual(values = c("LUSC" = "skyblue1")) +
  guides(colour = guide_legend(override.aes = list(size = 1))) +
  ggtitle("GSE157009") + stat_cor(
    aes(label = paste(..r.label..,..p.label.., sep = "~`,`~")), colour = "black", size = 4, label.x = Inf, label.y = Inf, hjust = 1, vjust = 1,
    method = "spearman") +
  theme(axis.title.x = element_text(size=12),
        axis.title.y = element_text(size = 12),
        legend.text = element_text(size=12),
        plot.title = element_text(size = 14)) +xlab("BM (ssGSEA score)") +ylab("ALV (ssGSEA score)")


#GSE157010

ssGSEA_Dev_GSE157010 <- GSVA::ssgseaParam(expr=GSE157010_matrix_filtered[,],
                                   Dev.sig_list)
ssGSEA_Dev_GSE157010 <- gsva(ssGSEA_Dev_GSE157010, verbose = T)

GSE157010_GEO_traits <- merge(GSE157010_GEO_traits, t(ssGSEA_Dev_GSE157010), by = 0)
rownames(GSE157010_GEO_traits) <- GSE157010_GEO_traits$Row.names
GSE157010_GEO_traits <- GSE157010_GEO_traits[,-1]
GSE157010_GEO_traits$Subtype <- "LUSC"

GSE157010_neg_cor <- GSE157010_GEO_traits %>%
  ggplot(aes(y = ALV, x = BM, colour = Subtype)) +
  theme_pubr(base_size = 7) +
  theme(legend.title = element_blank(), legend.key.height = unit(1,"pt"),
        legend.key.width = unit(1,"pt"),
        legend.position = c(0,0), legend.justification = c(0,0),
        legend.background = element_blank())+
  geom_point(size = 0.5) +
  stat_smooth(method = "lm", colour = "grey30") +
  scale_color_manual(values = c("LUSC" = "skyblue1")) +
  guides(colour = guide_legend(override.aes = list(size = 1))) +
  ggtitle("GSE157010") + stat_cor(
    aes(label = paste(..r.label..,..p.label.., sep = "~`,`~")), colour = "black", size = 4, label.x = Inf, label.y = Inf, hjust = 1, vjust = 1,
    method = "spearman") +
  theme(axis.title.x = element_text(size=12),
        axis.title.y = element_text(size = 12),
        legend.text = element_text(size=12),
        plot.title = element_text(size = 14)) +xlab("BM (ssGSEA score)") +ylab("ALV (ssGSEA score)")

#
ssGSEA_Dev_GSE4573 <- GSVA::ssgseaParam(expr=GSE4573_matrix_filtered[,],
                                 Dev.sig_list)
ssGSEA_Dev_GSE4573 <- gsva(ssGSEA_Dev_GSE4573, verbose = T)

GSE4573_traits <- merge(GSE4573_traits, t(ssGSEA_Dev_GSE4573), by = 0)
rownames(GSE4573_traits) <- GSE4573_traits$Row.names
GSE4573_traits <- GSE4573_traits[,-1]
GSE4573_traits$Subtype <- "LUSC"


GSE4573_neg_cor <- GSE4573_traits %>%
  ggplot(aes(y = ALV, x = BM, colour = Subtype)) +
  theme_pubr(base_size = 7) +
  theme(legend.title = element_blank(), legend.key.height = unit(1,"pt"),
        legend.key.width = unit(1,"pt"),
        legend.position = c(0,0), legend.justification = c(0,0),
        legend.background = element_blank())+
  geom_point(size = 0.5) +
  stat_smooth(method = "lm", colour = "grey30") +
  scale_color_manual(values = c("LUSC" = "skyblue1")) +
  guides(colour = guide_legend(override.aes = list(size = 1))) +
  ggtitle("GSE4573") + stat_cor(
    aes(label = paste(..r.label..,..p.label.., sep = "~`,`~")), colour = "black", size = 4, label.x = Inf, label.y = Inf, hjust = 1, vjust = 1,
    method = "spearman") +
  theme(axis.title.x = element_text(size=12),
        axis.title.y = element_text(size = 12),
        legend.text = element_text(size=12),
        plot.title = element_text(size = 14)) +xlab("BM (ssGSEA score)") +ylab("ALV (ssGSEA score)")

microarrays <-GSE72094_neg_cor+Okayama_neg_cor+Shedden_neg_cor+GSE157009_neg_cor+GSE157010_neg_cor+GSE4573_neg_cor
microarrays
```

```
ggsave(microarrays, path = Plots_out, file = "Figure_S1K.svg",
       width = 7, height = 5, units = "cm")
```

Figure S1L

```
# Tumour location - TCGA

combined <- NSCLC_traits_all
combined$Subtype <- as.character(combined$Subtype)
combined$Subtype[combined$Subtype == "LUSC"] <- "NSCLC"
combined$Subtype[combined$Subtype == "LUAD"] <- "NSCLC"

combined2 <- rbind(NSCLC_traits_all, combined)

Figure_S1Li <- combined2 %>% drop_na(Location) %>%
  ggplot(aes(x = Location, y = ALV, fill = Location)) +
  theme_pubr(base_size = 7) +
  geom_jitter(alpha = 0.5, size = 0.1, width = 0.2) +
  geom_boxplot(outlier.shape = NA) +
  ggpubr::geom_pwc(method = "wilcox_test", p.adjust.method = "fdr", label = "p.adj.format",
                   label.size = 2, tip.length = 0, vjust = 0.5, hide.ns = F) +
  scale_y_continuous(expand = expansion(mult = c(0, 0.10))) +
  facet_wrap(~Subtype) + ylab("ALV\n(ssGSEA score)") +
  scale_fill_manual(values = c("#794DFF", "#FF6341")) + 
  theme(axis.title.x = element_blank(), axis.text.x = element_blank())


Figure_S1Lii <- combined2 %>% drop_na(Location) %>%
  ggplot(aes(x = Location, y = BM, fill = Location)) +
  theme_pubr(base_size = 7) +
  geom_jitter(alpha = 0.5, size = 0.1, width = 0.2) +
  geom_boxplot(outlier.shape = NA) +
  ggpubr::geom_pwc(method = "wilcox_test", p.adjust.method = "fdr", label = "p.adj.signif", 
                   label.size = 2, tip.length = 0, vjust = 0.5, hide.ns = "p.adj") +
  scale_y_continuous(expand = expansion(mult = c(0, 0.10))) +
  facet_wrap(~Subtype) +
  ylab("BM\n(ssGSEA score)") +
  scale_fill_manual(values = c("#794DFF", "#FF6341")) + 
  theme(axis.title.x = element_blank(), axis.text.x = element_blank())

Figure_S1L <- ggarrange(Figure_S1Li, Figure_S1Lii, ncol = 1, common.legend = T, legend = "right")

ggsave(Figure_S1L, path = Plots_out, file = "Figure_S1L.svg",
       width = 7, height = 5, units = "cm")
Figure_S1L
```

Add dataset info

```
GSE72094_GEO_traits$Dataset <- "GSE72094"
Okayama_GEO_traits2$Dataset <- "Okayama"
Shedden_GEO_traits$Dataset <- "Shedden"
NSCLC_traits_all$Dataset <- "TCGA"
GSE157009_GEO_traits$Dataset <- "GSE157009"
GSE157010_GEO_traits$Dataset <- "GSE157010"
GSE4573_traits$Dataset <- "GSE4573"
```

Separate TCGA into LUAD and LUSC

```
LUAD_traits_TCGA <- subset(NSCLC_traits_all, Subtype == "LUAD")
LUAD_traits_TCGA$Dataset <- "TCGA"
LUSC_traits_TCGA <- subset(NSCLC_traits_all, Subtype == "LUSC")
LUSC_traits_TCGA$Dataset <- "TCGA"
```

Merge LUAD traits

```
merged_LUAD_traits <- rbind(Shedden_GEO_traits[, c("BM", "ALV", "OS_MONTHS", "OS", "Subtype", "Dataset")],
                Okayama_GEO_traits2[, c("BM", "ALV", "OS_MONTHS", "OS", "Subtype", "Dataset")])

merged_LUAD_traits <- rbind(merged_LUAD_traits[, c("BM", "ALV", "OS_MONTHS", "OS", "Subtype", "Dataset")],
               GSE72094_GEO_traits[, c("BM", "ALV", "OS_MONTHS", "OS", "Subtype", "Dataset")])

merged_LUAD_traits <- rbind(merged_LUAD_traits[, c("BM", "ALV", "OS_MONTHS", "OS", "Subtype", "Dataset")],
               LUAD_traits_TCGA[, c("BM", "ALV", "OS_MONTHS", "OS", "Subtype", "Dataset")])
```

Merge LUSC traits

```
merged_LUSC_traits <- rbind(GSE157009_GEO_traits[, c("BM", "ALV", "OS_MONTHS", "OS", "Subtype", "Dataset")],
                GSE157010_GEO_traits[, c("BM", "ALV", "OS_MONTHS", "OS", "Subtype", "Dataset")])

merged_LUSC_traits <- rbind(merged_LUSC_traits[, c("BM", "ALV", "OS_MONTHS", "OS", "Subtype", "Dataset")],
               GSE4573_traits[, c("BM", "ALV", "OS_MONTHS", "OS", "Subtype", "Dataset")])

merged_LUSC_traits <- rbind(merged_LUSC_traits[, c("BM", "ALV", "OS_MONTHS", "OS", "Subtype", "Dataset")],
               LUSC_traits_TCGA[, c("BM", "ALV", "OS_MONTHS", "OS", "Subtype", "Dataset")])
```

```
setwd(input_files)
save(merged_LUAD_traits, file = "Merged_LUAD_traits.Rdata")
save(merged_LUSC_traits, file = "Merged_LUSC_traits.Rdata")
```

## Session Info

```
print(sessionInfo(), RNG = TRUE, locale = FALSE)
```

```
## R version 4.4.0 (2024-04-24 ucrt)
## Platform: x86_64-w64-mingw32/x64
## Running under: Windows 10 x64 (build 19045)
## 
## Matrix products: default
## 
## 
## Random number generation:
##  RNG:     Mersenne-Twister 
##  Normal:  Inversion 
##  Sample:  Rejection 
##  
## attached base packages:
## [1] splines   stats4    stats     graphics  grDevices utils     datasets 
## [8] methods   base     
## 
## other attached packages:
##  [1] tidyr_1.3.1            stringr_1.5.1          gam_1.22-5            
##  [4] foreach_1.5.2          mgcv_1.9-1             nlme_3.1-167          
##  [7] org.Mm.eg.db_3.20.0    AnnotationDbi_1.68.0   IRanges_2.40.1        
## [10] S4Vectors_0.44.0       Biobase_2.66.0         BiocGenerics_0.52.0   
## [13] nichenetr_2.2.1.1      dplyr_1.1.4            ggpubr_0.6.0          
## [16] clusterProfiler_4.14.4 biomaRt_2.62.1         factoextra_1.0.7      
## [19] ggplot2_3.5.1          ggsci_3.2.0            GSVA_2.0.5            
## 
## loaded via a namespace (and not attached):
##   [1] SpatialExperiment_1.16.0    R.methodsS3_1.8.2          
##   [3] GSEABase_1.68.0             progress_1.2.3             
##   [5] nnet_7.3-20                 goftest_1.2-3              
##   [7] Biostrings_2.74.1           HDF5Array_1.34.0           
##   [9] vctrs_0.6.5                 ggtangle_0.0.6             
##  [11] spatstat.random_3.3-2       shape_1.4.6.1              
##  [13] digest_0.6.35               png_0.1-8                  
##  [15] proxy_0.4-27                ggrepel_0.9.6              
##  [17] deldir_2.0-4                parallelly_1.42.0          
##  [19] magick_2.8.5                MASS_7.3-64                
##  [21] reshape2_1.4.4              httpuv_1.6.15              
##  [23] qvalue_2.38.0               withr_3.0.2                
##  [25] ggrastr_1.0.2               xfun_0.50                  
##  [27] ggfun_0.1.8                 survival_3.8-3             
##  [29] memoise_2.0.1               ggbeeswarm_0.7.2           
##  [31] gson_0.1.0                  systemfonts_1.3.1          
##  [33] ragg_1.5.0                  GlobalOptions_0.1.2        
##  [35] tidytree_0.4.6              zoo_1.8-12                 
##  [37] pbapply_1.7-2               R.oo_1.27.0                
##  [39] Formula_1.2-5               prettyunits_1.2.0          
##  [41] KEGGREST_1.46.0             promises_1.3.2             
##  [43] httr_1.4.7                  rstatix_0.7.2              
##  [45] globals_0.16.3              fitdistrplus_1.2-2         
##  [47] rhdf5filters_1.18.0         rhdf5_2.50.2               
##  [49] rstudioapi_0.17.1           UCSC.utils_1.2.0           
##  [51] miniUI_0.1.1.1              generics_0.1.3             
##  [53] DOSE_4.0.0                  base64enc_0.1-3            
##  [55] curl_6.2.0                  zlibbioc_1.52.0            
##  [57] ScaledMatrix_1.14.0         randomForest_4.7-1.2       
##  [59] polyclip_1.10-7             GenomeInfoDbData_1.2.13    
##  [61] SparseArray_1.6.1           doParallel_1.0.17          
##  [63] xtable_1.8-4                evaluate_1.0.3             
##  [65] S4Arrays_1.6.0              preprocessCore_1.68.0      
##  [67] BiocFileCache_2.14.0        hms_1.1.3                  
##  [69] GenomicRanges_1.58.0        irlba_2.3.5.1              
##  [71] visNetwork_2.1.4            colorspace_2.1-1           
##  [73] filelock_1.0.3              ROCR_1.0-11                
##  [75] readxl_1.4.3                reticulate_1.40.0          
##  [77] spatstat.data_3.1-4         magrittr_2.0.3             
##  [79] lmtest_0.9-40               readr_2.1.5                
##  [81] later_1.4.1                 ggtree_3.14.0              
##  [83] lattice_0.22-6              spatstat.geom_3.3-5        
##  [85] future.apply_1.11.3         scattermore_1.2            
##  [87] XML_3.99-0.18               shadowtext_0.1.4           
##  [89] cowplot_1.1.3               matrixStats_1.5.0          
##  [91] RcppAnnoy_0.0.22            Hmisc_5.2-2                
##  [93] class_7.3-23                pillar_1.10.1              
##  [95] iterators_1.0.14            caTools_1.18.3             
##  [97] compiler_4.4.0              beachmat_2.22.0            
##  [99] RSpectra_0.16-2             stringi_1.8.4              
## [101] gower_1.0.2                 tensor_1.5                 
## [103] SummarizedExperiment_1.36.0 lubridate_1.9.4            
## [105] plyr_1.8.9                  crayon_1.5.3               
## [107] abind_1.4-8                 gridGraphics_0.5-1         
## [109] sp_2.2-0                    bit_4.5.0.1                
## [111] fastmatch_1.1-6             fastcluster_1.2.6          
## [113] textshaping_1.0.0           codetools_0.2-20           
## [115] recipes_1.1.0               BiocSingular_1.22.0        
## [117] bslib_0.9.0                 e1071_1.7-16               
## [119] GetoptLong_1.0.5            plotly_4.10.4              
## [121] mime_0.12                   circlize_0.4.16            
## [123] Rcpp_1.0.14                 fastDummies_1.7.5          
## [125] dbplyr_2.5.0                sparseMatrixStats_1.18.0   
## [127] cellranger_1.1.0            knitr_1.49                 
## [129] blob_1.2.4                  clue_0.3-66                
## [131] fs_1.6.5                    checkmate_2.3.2            
## [133] listenv_0.9.1               ggsignif_0.6.4             
## [135] ggplotify_0.1.2             tibble_3.2.1               
## [137] Matrix_1.7-2                statmod_1.5.0              
## [139] svglite_2.2.2               tzdb_0.4.0                 
## [141] tweenr_2.0.3                pkgconfig_2.0.3            
## [143] tools_4.4.0                 cachem_1.1.0               
## [145] RSQLite_2.3.9               viridisLite_0.4.2          
## [147] DBI_1.2.3                   impute_1.80.0              
## [149] fastmap_1.2.0               rmarkdown_2.29             
## [151] scales_1.3.0                grid_4.4.0                 
## [153] ica_1.0-3                   Seurat_5.2.1               
## [155] broom_1.0.7                 sass_0.4.9                 
## [157] patchwork_1.3.0             dotCall64_1.2              
## [159] graph_1.84.1                carData_3.0-5              
## [161] RANN_2.6.2                  rpart_4.1.24               
## [163] farver_2.1.2                yaml_2.3.10                
## [165] foreign_0.8-88              DiagrammeR_1.0.11          
## [167] MatrixGenerics_1.18.1       cli_3.6.2                  
## [169] purrr_1.0.4                 GEOquery_2.74.0            
## [171] lifecycle_1.0.4             caret_7.0-1                
## [173] uwot_0.2.2                  lava_1.8.1                 
## [175] backports_1.5.0             BiocParallel_1.40.0        
## [177] annotate_1.84.0             timechange_0.3.0           
## [179] gtable_0.3.6                rjson_0.2.23               
## [181] ggridges_0.5.6              progressr_0.15.1           
## [183] limma_3.62.2                parallel_4.4.0             
## [185] pROC_1.18.5                 ape_5.8-1                  
## [187] jsonlite_1.8.9              bitops_1.0-9               
## [189] RcppHNSW_0.6.0              bit64_4.6.0-1              
## [191] Rtsne_0.17                  yulab.utils_0.2.0          
## [193] spatstat.utils_3.1-2        SeuratObject_5.0.2         
## [195] jquerylib_0.1.4             GOSemSim_2.32.0            
## [197] spatstat.univar_3.1-1       R.utils_2.12.3             
## [199] timeDate_4041.110           lazyeval_0.2.2             
## [201] dynamicTreeCut_1.63-1       shiny_1.10.0               
## [203] htmltools_0.5.8.1           enrichplot_1.26.6          
## [205] GO.db_3.20.0                sctransform_0.4.1          
## [207] rappdirs_0.3.3              glue_1.7.0                 
## [209] spam_2.11-1                 httr2_1.1.0                
## [211] XVector_0.46.0              treeio_1.30.0              
## [213] gridExtra_2.3               igraph_2.1.4               
## [215] R6_2.5.1                    fdrtool_1.2.18             
## [217] SingleCellExperiment_1.28.1 labeling_0.4.3             
## [219] cluster_2.1.8               Rhdf5lib_1.28.0            
## [221] aplot_0.2.4                 GenomeInfoDb_1.42.3        
## [223] ipred_0.9-15                WGCNA_1.73                 
## [225] vipor_0.4.7                 DelayedArray_0.32.0        
## [227] tidyselect_1.2.1            htmlTable_2.4.3            
## [229] ggforce_0.4.2               xml2_1.3.6                 
## [231] car_3.1-3                   future_1.34.0              
## [233] ModelMetrics_1.2.2.2        rsvd_1.0.5                 
## [235] munsell_0.5.1               KernSmooth_2.23-26         
## [237] data.table_1.15.4           ComplexHeatmap_2.22.0      
## [239] htmlwidgets_1.6.4           fgsea_1.32.2               
## [241] RColorBrewer_1.1-3          rlang_1.1.4                
## [243] spatstat.sparse_3.1-0       spatstat.explore_3.3-4     
## [245] rentrez_1.2.3               Cairo_1.7-0                
## [247] ggnewscale_0.5.0            hardhat_1.4.1              
## [249] beeswarm_0.4.0              prodlim_2024.06.25
```
