# Supplementary material for: Developmental programmes drive cellular plasticity, disease progression and therapy resistance in lung adenocarcinoma
Source: Mol Oncol. 2026 May 27:10.1002/1878-0261.70263. Online ahead of print. doi: 10.1002/1878-0261.70263 (PMC13398952; doi:10.1002/1878-0261.70263)
Supplement: Supplementary file 1 — File 1. R Markdown HTML reports. [file MOL2-9999-0-s006.zip › Bienkowska_etal_MolOnc_Fig2.html]

Developmental programmes drive cellular plasticity, disease progression and therapy resistance in lung adenocarcinoma


# Developmental programmes drive cellular plasticity, disease progression and therapy resistance in lung adenocarcinoma

### Figure 2 - High expression of BM in LUAD predicts poor overall survival and is associated with disease progression

#### Kamila J Bienkowska, Stephany Gallardo Y, Nur S Zainal, Leena Arora, Matthew Ellis, Maria-Antoinette Lopez, Judith Austine, Sai Pittla, Serena J Chee, Aiman Alzetani, Emily C Shaw, Christian H Ottensmeier, Gareth J Thomas, Christopher J Hanley

#### 2025-08-27

## Load packages

```
library(tidyverse)
library(ggpubr)
library(ggsci)
library(survival)
library(survminer)
library(forestmodel)
library(readxl)
library(GSVA)
library(edgeR)
library(Seurat)
library(maftools)
library(TCGAmutations)
library(tidyr)
library(BSgenome.Hsapiens.UCSC.hg19)
library(NMF)
```

## Load objects

```
setwd(input_files)
load(file = "Merged_LUSC_traits.Rdata")
load(file = "Merged_LUAD_traits.Rdata")
load(file = "GSE103584_recount.Rdata")
load(file="MergedLUADTraits.Rdata")
load(file="MergedLUSCTraits.Rdata")
load(file="Alveogenesis_signature_modified.Rdata")
load(file="Morphogenesis_signature_modified.Rdata")
load(file="NSCLC_traits_all.Rdata")
load(file="NSCLC_vsd_all.Rdata")
load("Kim_Epi_seurat.Rdata")
load("Kim_Epi_pseudobulk.Rdata")
```

```
# #Adding GSE103584 dataset ALV/BM scores
rownames(lcpm.filtered.norm) <- gene.meta.data$gene_name[match(rownames(lcpm.filtered.norm), rownames(gene.meta.data))]

Dev.sig_list <- list(
  ALV = Alveogenesis_sig,
  BM = Morphogenesis_sig
)

ssGSEA_RG <- GSVA::ssgseaParam(expr=lcpm.filtered.norm, 
                               Dev.sig_list)

ssGSEA_RG <- gsva(ssGSEA_RG, verbose = T)

sample.meta.data <- merge(sample.meta.data, t(ssGSEA_RG), by = 0)
rownames(sample.meta.data) <- sample.meta.data$Row.names
sample.meta.data <- sample.meta.data[,-1]
sample.meta.data$Sample.ID <- sample.meta.data$external_id
sample.meta.data$Dataset <- "GSE103584"

merged_LUAD_traits$Sample.ID <- rownames(merged_LUAD_traits)
merged_LUSC_traits$Sample.ID <- rownames(merged_LUSC_traits)

merged_ALV.BM <- rbind(merged_LUAD_traits[, c("BM", "ALV", "Sample.ID")],
                       sample.meta.data[, c("BM", "ALV", "Sample.ID")],
                       merged_LUSC_traits[, c("BM", "ALV", "Sample.ID")])
save(merged_ALV.BM, file = "Bulk_ALV.BM_scores.Rdata")

merged_LUAD_traits <- merge(merged_ALV.BM, Merged.LUADtraits, by = "Sample.ID", all.x = F, all.y = T)
```

## Figures

### Figure S2A - LUAD survival analysis split by dataset

```
rownames(merged_LUAD_traits) <- merged_LUAD_traits$Sample.ID
merged_LUAD_traits$OS_to5Y <- merged_LUAD_traits$OS_YEARS
merged_LUAD_traits$OS_to5Y[merged_LUAD_traits$OS_YEARS > 5] <- 5
merged_LUAD_traits$OS_to5Y_event <- merged_LUAD_traits$OS
merged_LUAD_traits$OS_to5Y_event[merged_LUAD_traits$OS_YEARS > 5] <- 0

merged_LUAD_traits$Dataset <- as.factor(merged_LUAD_traits$Dataset)
Opt_cat_list <- list()
for(i in levels(merged_LUAD_traits$Dataset)){
  Opt_cut <- surv_cutpoint(merged_LUAD_traits[merged_LUAD_traits$Dataset == i, ],
                           time = "OS_to5Y", event = "OS_to5Y_event",
                           "BM",
                           minprop = 0.1, progressbar = TRUE)
  
  Opt_Cat <- surv_categorize(Opt_cut, variables = NULL,
                             labels = c("Low", "High"))
  Opt_Cat$BM <- factor(
    Opt_Cat$BM,
    levels = c("Low", "High")
  )
  Opt_Cat$Sample.ID <- rownames(merged_LUAD_traits)[merged_LUAD_traits$Dataset == i]
  Opt_cat_list[[i]] <- Opt_Cat
  Opt_cat_list[[i]]$cutpoint <- Opt_cut$cutpoint$cutpoint
}
Opt_Cat <- do.call(rbind, Opt_cat_list)
merged_LUAD_5d <- merge(merged_LUAD_traits, Opt_Cat, by = "Sample.ID")

merged_LUAD_5d$Dataset <- factor(merged_LUAD_5d$Dataset, levels = c("TCGA", "GSE72094", "Shedden", "Okayama", "GSE103584"),
                                 labels = c("TCGA", "GSE72094", "GSE68465", "GSE31210", "GSE103584"))


Figure_S2A_surv <- ggsurvplot(survfit(Surv(OS_to5Y.x, OS_to5Y_event.x) ~  BM.y, data = merged_LUAD_5d),
                      data = merged_LUAD_5d,
                      facet.by = c("Dataset"), ncol = 5,
                      short.panel.labs = T,
                      pval = TRUE, pval.coord = c(0,0.1), pval.size = 2,
                      legend.labs = c("BM-Low", "BM-High"),
                      conf.int = T, conf.int.alpha = 0.05,
                      theme = "theme_pubr",
                      palette = "aaas",
                      censor.size = 1, censor.shape = 124,
                      risk.table = T
                      
) 
Figure_S2A <- Figure_S2A_surv + xlab("Time (years)") + ylab ("OS probability") + 
  theme_pubr(base_size = 7) + ggtitle("LUAD Cohorts") + theme(legend.position = "right")
Figure_S2A
```

```
ggsave(Figure_S2A, path = Plots_out, file = "Figure_S2A.svg",
       width = 18, height = 5, units = "cm")
```

### Figure S2B - LUSC datasets

```
# LUSC ####
merged_LUSC_traits <- merge(merged_ALV.BM, Merged.LUSCtraits, by = "Sample.ID", all.x = F, all.y = T)
rownames(merged_LUSC_traits) <- merged_LUSC_traits$Sample.ID
merged_LUSC_traits$OS_to5Y <- merged_LUSC_traits$OS_YEARS
merged_LUSC_traits$OS_to5Y[merged_LUSC_traits$OS_YEARS > 5] <- 5
merged_LUSC_traits$OS_to5Y_event <- merged_LUSC_traits$OS
merged_LUSC_traits$OS_to5Y_event[merged_LUSC_traits$OS_YEARS > 5] <- 0

merged_LUSC_traits$Dataset <- as.factor(merged_LUSC_traits$Dataset)
Opt_cat_list <- list()
for(i in levels(merged_LUSC_traits$Dataset)){
  Opt_cut <- surv_cutpoint(merged_LUSC_traits[merged_LUSC_traits$Dataset == i, ],
                           time = "OS_to5Y", event = "OS_to5Y_event",
                           "BM",
                           minprop = 0.1, progressbar = TRUE)
  
  Opt_Cat <- surv_categorize(Opt_cut, variables = NULL,
                             labels = c("Low", "High"))
  Opt_Cat$BM <- factor(
    Opt_Cat$BM,
    levels = c("Low", "High")
  )
  Opt_Cat$Sample.ID <- rownames(merged_LUSC_traits)[merged_LUSC_traits$Dataset == i]
  Opt_cat_list[[i]] <- Opt_Cat
  Opt_cat_list[[i]]$cutpoint <- Opt_cut$cutpoint$cutpoint
}
Opt_Cat <- do.call(rbind, Opt_cat_list)
merged_LUSC_5d <- merge(merged_LUSC_traits, Opt_Cat, by = "Sample.ID")

merged_LUSC_5d$Dataset <- factor(merged_LUSC_5d$Dataset, levels = c("TCGA", "GSE157009", "GSE157010", "GSE4573", "GSE103584"))

Figure_S2B_surv <- ggsurvplot(survfit(Surv(OS_to5Y.x, OS_to5Y_event.x) ~  BM.y, data = merged_LUSC_5d),
                      data = merged_LUSC_5d,
                      facet.by = c("Dataset"), ncol = 5,
                      short.panel.labs = T,
                      pval = TRUE, pval.coord = c(0,0.1), pval.size = 2,
                      legend.labs = c("BM-Low", "BM-High"),
                      conf.int = T, conf.int.alpha = 0.05,
                      theme = "theme_pubr",
                      palette = "aaas",
                      censor.size = 1, censor.shape = 124,
                      risk.table = T
                      
) 
Figure_S2B <- Figure_S2B_surv + xlab("Time (years)") + ylab ("OS probability") + 
  theme_pubr(base_size = 7) + ggtitle("LUSC Cohorts") + theme(legend.position = "right")
Figure_S2B
```

```
ggsave(Figure_S2B, path = Plots_out, file = "Figure_S2B.svg",
       width = 18, height = 5, units = "cm")
```

### Figure 2A

```
Figure_2A_surv <- ggsurvplot(survfit(Surv(OS_to5Y.x, OS_to5Y_event.x) ~  BM.y , data = merged_LUAD_5d),
                          data = merged_LUAD_5d,
                          pval = TRUE, pval.coord = c(4,0), pval.size = 2,
                          conf.int = T, conf.int.alpha =0.05,
                          theme = "theme_pubr",
                          palette = "aaas",
                          legend.labs = c("BM-Low", "BM-High"),
                          censor.size = 1, censor.shape = 124,
                          risk.table = T, fontsize = 2, legend.title="") +
  theme_survminer(base_size = 7,
                  font.legend = c(7, "plain", "black"),
                  font.y = c(7, "bold"), 
                  font.submain = c(7, "plain", "black"))

Figure_2A <- 
  ggarrange(Figure_2A_surv$plot + theme_pubr(base_size = 7) + theme(legend.position = c(0,0), legend.justification = c(-0.1,0), legend.key.size = unit(5,"pt"), axis.title.x = element_blank(), axis.text.x = element_blank(), legend.background = element_rect(fill='transparent')),
            Figure_2A_surv$table + theme_pubr(base_size = 7) + theme(plot.title = element_blank()) + xlab("Time (Years)"),
            heights = c(2.5,1), nrow = 2, align = "v")
Figure_2A
```

```
ggsave(Figure_2A, path = Plots_out, file = "Figure_2A.svg",
       width = 7, height = 5, units = "cm")
```

### Figure 2B

```
merged_LUAD_5d$Age <- as.numeric(merged_LUAD_5d$Age)
cox_model_data <- merged_LUAD_5d[, c("OS_to5Y.x", "OS_to5Y_event.x", "BM.y", "Stage_4cat", "Age")]
names(cox_model_data) <- c("OS_to5Y.x", "OS_to5Y_event.x", "BM", "Stage_4cat", "Age")
summary(coxph(Surv(OS_to5Y.x, OS_to5Y_event.x) ~  BM.y + Stage_4cat + Age, data = merged_LUAD_5d))
BM_cox.res <- coxph(Surv(OS_to5Y.x, OS_to5Y_event.x) ~  BM + Stage_4cat + Age, data = cox_model_data)
Figure_2B <- forest_model(BM_cox.res, format_options = forest_model_format_options(text_size = 2, point_size = 2),
                            recalculate_width = T, return_data = T)
ggsave(Figure_2B$plot,
       path = Plots_out, file = "Figure_2B.svg", width = 10, height = 5, units = "cm")

Figure_2B$plot
```

### Figure 2C

```
Dev.sig_list <- list(
  ALV = Alveogenesis_sig,
  BM = Morphogenesis_sig
)

Kim_Epi_pseudobulk$Sample_origin2 <- factor(
  Kim_Epi_pseudobulk$Sample_origin,
  levels = c("nLung",  "tLung",  "tL/B", "mLN", "PE", "mBrain"),
  labels = c("Control", "Early-stage", "Advanced-stage", "Lymph Nodes", "Pleural Effusion", "Brain")
)
Kim_Epi_pseudobulk$Sample_origin3 <- factor(
  Kim_Epi_pseudobulk$Sample_origin,
  levels = c("nLung",  "tLung",  "tL/B", "mLN", "PE", "mBrain"),
  labels = c("Primary", "Primary", "Primary", "Metastasis", "Metastasis", "Metastasis")
)

ssGSEA_Kim.Epi_pseudobulk <- GSVA::ssgseaParam(expr=as.matrix(Kim_Epi_pseudobulk@assays$RNA@data),
                                      Dev.sig_list)

ssGSEA_Kim.Epi_pseudobulk  <- gsva(ssGSEA_Kim.Epi_pseudobulk , verbose = T) 
rownames(Kim_Epi_pseudobulk@meta.data) <- Kim_Epi_pseudobulk@meta.data$Row.names
Kim_Epi_pseudobulk@meta.data <- merge(Kim_Epi_pseudobulk@meta.data, t(ssGSEA_Kim.Epi_pseudobulk), by = 0)
Kim_Epi_pseudobulk@meta.data <- Kim_Epi_pseudobulk@meta.data[,-c(1,2)]

Figure_2C <- Kim_Epi_pseudobulk@meta.data %>%
  mutate(Sample_origin2.1 = factor(Sample_origin2, labels = gsub("-| ", "\n", levels(Sample_origin2)))) %>%
  ggplot(aes(x = Sample_origin2.1, y = BM, fill = Sample_origin2)) +
  theme_pubr(base_size = 7) +
  geom_boxplot(outlier.shape = NA) +
  geom_jitter(width = 0.2, size = 0.5) +
  rotate_x_text(angle = 45) +
  ggpubr::geom_pwc(method = "wilcox_test", ref.group = "Early\nstage", label = "p.adj.signif", p.adjust.method = "fdr", label.size = 2, tip.length = 0, step.increase = 0.1, y.position = 1.1) +
  expand_limits(y = 1.25) +
  theme(axis.title.x = element_blank()) +
  ylab("BM (ssGSEA score)") + scale_fill_manual(values = c("#5435B2", "#794DFF", "#E0CBFF", "#FFD3C4", "#FF6341", "#FF3306")) + NoLegend()
Figure_2C
```

```
ggsave(Figure_2C, path = Plots_out, file = "Figure_2C.svg", width = 6, height = 5, units = "cm")
```

### Figure 2D&E

```
Kim_Epi_seurat <- AddModuleScore(Kim_Epi_seurat, features = Dev.sig_list)

thresh_ALV = median(Kim_Epi_seurat$Cluster1)+mad(Kim_Epi_seurat$Cluster1)
thresh_BM = median(Kim_Epi_seurat$Cluster2)+mad(Kim_Epi_seurat$Cluster2)

Kim_Epi_seurat$Alv_BM.class <- 
  ifelse(Kim_Epi_seurat$Cluster1 > thresh_ALV & Kim_Epi_seurat$Cluster2 < thresh_BM, "ALV+BM-", 
         ifelse(Kim_Epi_seurat$Cluster1 < thresh_ALV & Kim_Epi_seurat$Cluster2 < thresh_BM, "ALV-BM-", 
                ifelse(Kim_Epi_seurat$Cluster1 < thresh_ALV & Kim_Epi_seurat$Cluster2 > thresh_BM, "ALV-BM+", "ALV+BM+")))

Figure_2D <- Kim_Epi_seurat@meta.data %>%
  ggplot(aes(x = Cluster1, y = Cluster2, colour = Alv_BM.class)) +
  theme_pubr(base_size = 7) +
  scattermore::geom_scattermore(pointsize = 2, dpi = 521) +
  theme(legend.position = "none") +
  geom_hline(yintercept = thresh_BM, linetype = "dotted") + geom_vline(xintercept = thresh_ALV, linetype = "dotted") +
  xlab("ALV (module score)") + ylab("BM (module score)") + scale_color_manual(values = c("dodgerblue3", "darkorange3", "forestgreen", "maroon2"))

ggsave(Figure_2D, path = Plots_out, file = "Figure_2D.svg", width = 5, height = 5, units = "cm")

Kim_Epi_seurat$Sample_origin2 <- factor(
  Kim_Epi_seurat$Sample_Origin,
  levels = c("nLung",  "tLung",  "tL/B", "mLN", "PE", "mBrain"),
  labels = c("Control", "Early-stage", "Advanced-stage", "Lymph Nodes", "Pleural Effusion", "Brain")
)

Kim_Epi_seurat$Sample_origin3 <- factor(
  Kim_Epi_seurat$Sample_Origin,
  levels = c("nLung",  "tLung",  "tL/B", "mLN", "PE", "mBrain"),
  labels = c("Primary", "Primary", "Primary", "Metastasis", "Metastasis", "Metastasis")
)


Figure_2E <- 
 Kim_Epi_seurat@meta.data %>%
  ggplot(aes(x = Sample_origin2, fill = Alv_BM.class)) +
  theme_pubr(base_size = 7) +
  geom_bar(position = "fill") +
  facet_wrap(~Sample_origin3, scales = "free_x") +
  xlab("Biopsy site") + 
  ylab("Fraction of All Epithelial Cells") +
  rotate_x_text(angle = 45) + 
  theme(legend.position = "right", legend.title = element_blank(),
        legend.key.size = unit(5, "pt"))+
  scale_fill_manual(values = c("dodgerblue3", "darkorange3", "forestgreen", "maroon2"))

ggsave(Figure_2E, path = Plots_out, file = "Figure_2E.svg", width = 5, height = 5, units = "cm")

Figure_2D + Figure_2E
```

## Session Info

```
print(sessionInfo(), RNG = TRUE, locale = FALSE)
```

```
## R version 4.4.0 (2024-04-24 ucrt)
## Platform: x86_64-w64-mingw32/x64
## Running under: Windows 11 x64 (build 26100)
## 
## Matrix products: default
## 
## 
## Random number generation:
##  RNG:     Mersenne-Twister 
##  Normal:  Inversion 
##  Sample:  Rejection 
##  
## attached base packages:
## [1] stats4    stats     graphics  grDevices utils     datasets  methods  
## [8] base     
## 
## other attached packages:
##  [1] NMF_0.28                          Biobase_2.64.0                   
##  [3] cluster_2.1.6                     rngtools_1.5.2                   
##  [5] registry_0.5-1                    BSgenome.Hsapiens.UCSC.hg19_1.4.3
##  [7] BSgenome_1.72.0                   rtracklayer_1.64.0               
##  [9] BiocIO_1.14.0                     Biostrings_2.72.1                
## [11] XVector_0.44.0                    GenomicRanges_1.56.2             
## [13] GenomeInfoDb_1.40.1               IRanges_2.38.1                   
## [15] S4Vectors_0.42.1                  BiocGenerics_0.50.0              
## [17] TCGAmutations_0.4.0               data.table_1.16.0                
## [19] maftools_2.20.0                   Seurat_5.2.0                     
## [21] SeuratObject_5.0.2                sp_2.1-4                         
## [23] edgeR_4.2.1                       limma_3.60.5                     
## [25] GSVA_1.52.3                       readxl_1.4.3                     
## [27] forestmodel_0.6.2                 survminer_0.5.0                  
## [29] survival_3.8-3                    ggsci_3.2.0                      
## [31] ggpubr_0.6.0                      lubridate_1.9.3                  
## [33] forcats_1.0.0                     stringr_1.5.1                    
## [35] dplyr_1.1.4                       purrr_1.0.2                      
## [37] readr_2.1.5                       tidyr_1.3.1                      
## [39] tibble_3.2.1                      ggplot2_3.5.1                    
## [41] tidyverse_2.0.0                  
## 
## loaded via a namespace (and not attached):
##   [1] ggtext_0.1.2                bitops_1.0-9               
##   [3] matrixStats_1.4.1           spatstat.sparse_3.1-0      
##   [5] doParallel_1.0.17           httr_1.4.7                 
##   [7] RColorBrewer_1.1-3          tools_4.4.0                
##   [9] sctransform_0.4.1           backports_1.5.0            
##  [11] R6_2.5.1                    HDF5Array_1.32.1           
##  [13] lazyeval_0.2.2              uwot_0.2.2                 
##  [15] rhdf5filters_1.16.0         withr_3.0.2                
##  [17] gridExtra_2.3               progressr_0.15.1           
##  [19] textshaping_0.4.1           cli_3.6.3                  
##  [21] exactRankTests_0.8-35       spatstat.explore_3.3-4     
##  [23] fastDummies_1.7.4           labeling_0.4.3             
##  [25] sass_0.4.9                  mvtnorm_1.3-3              
##  [27] survMisc_0.5.6              spatstat.data_3.1-4        
##  [29] ggridges_0.5.6              pbapply_1.7-2              
##  [31] systemfonts_1.1.0           Rsamtools_2.20.0           
##  [33] svglite_2.1.3               parallelly_1.41.0          
##  [35] rstudioapi_0.17.1           RSQLite_2.3.7              
##  [37] generics_0.1.3              ica_1.0-3                  
##  [39] spatstat.random_3.3-2       car_3.1-3                  
##  [41] Matrix_1.7-0                abind_1.4-8                
##  [43] lifecycle_1.0.4             yaml_2.3.10                
##  [45] carData_3.0-5               SummarizedExperiment_1.34.0
##  [47] rhdf5_2.48.0                SparseArray_1.4.8          
##  [49] Rtsne_0.17                  grid_4.4.0                 
##  [51] blob_1.2.4                  promises_1.3.2             
##  [53] crayon_1.5.3                miniUI_0.1.1.1             
##  [55] lattice_0.22-6              beachmat_2.20.0            
##  [57] cowplot_1.1.3               annotate_1.82.0            
##  [59] KEGGREST_1.44.1             magick_2.8.5               
##  [61] pillar_1.10.1               knitr_1.49                 
##  [63] rjson_0.2.23                future.apply_1.11.3        
##  [65] codetools_0.2-20            glue_1.8.0                 
##  [67] spatstat.univar_3.1-1       vctrs_0.6.5                
##  [69] png_0.1-8                   spam_2.11-0                
##  [71] cellranger_1.1.0            gtable_0.3.6               
##  [73] cachem_1.1.0                xfun_0.50                  
##  [75] S4Arrays_1.4.1              mime_0.12                  
##  [77] SingleCellExperiment_1.26.0 iterators_1.0.14           
##  [79] KMsurv_0.1-5                statmod_1.5.0              
##  [81] fitdistrplus_1.2-2          ROCR_1.0-11                
##  [83] nlme_3.1-166                bit64_4.5.2                
##  [85] RcppAnnoy_0.0.22            maxstat_0.7-25             
##  [87] bslib_0.8.0                 irlba_2.3.5.1              
##  [89] KernSmooth_2.23-26          colorspace_2.1-1           
##  [91] DBI_1.2.3                   DNAcopy_1.78.0             
##  [93] tidyselect_1.2.1            curl_6.1.0                 
##  [95] bit_4.5.0                   compiler_4.4.0             
##  [97] graph_1.82.0                xml2_1.3.6                 
##  [99] DelayedArray_0.30.1         plotly_4.10.4              
## [101] scales_1.3.0                lmtest_0.9-40              
## [103] SpatialExperiment_1.14.0    digest_0.6.37              
## [105] goftest_1.2-3               spatstat.utils_3.1-2       
## [107] rmarkdown_2.29              htmltools_0.5.8.1          
## [109] pkgconfig_2.0.3             sparseMatrixStats_1.16.0   
## [111] MatrixGenerics_1.16.0       fastmap_1.2.0              
## [113] rlang_1.1.4                 htmlwidgets_1.6.4          
## [115] UCSC.utils_1.0.0            shiny_1.10.0               
## [117] farver_2.1.2                jquerylib_0.1.4            
## [119] zoo_1.8-12                  jsonlite_1.8.9             
## [121] BiocParallel_1.38.0         RCurl_1.98-1.16            
## [123] BiocSingular_1.20.0         magrittr_2.0.3             
## [125] Formula_1.2-5               GenomeInfoDbData_1.2.12    
## [127] dotCall64_1.2               patchwork_1.3.0            
## [129] Rhdf5lib_1.26.0             munsell_0.5.1              
## [131] Rcpp_1.0.13                 reticulate_1.40.0          
## [133] stringi_1.8.4               zlibbioc_1.50.0            
## [135] MASS_7.3-61                 plyr_1.8.9                 
## [137] parallel_4.4.0              listenv_0.9.1              
## [139] ggrepel_0.9.6               deldir_2.0-4               
## [141] splines_4.4.0               gridtext_0.1.5             
## [143] tensor_1.5                  hms_1.1.3                  
## [145] locfit_1.5-9.10             igraph_2.0.3               
## [147] spatstat.geom_3.3-4         ggsignif_0.6.4             
## [149] RcppHNSW_0.6.0              reshape2_1.4.4             
## [151] ScaledMatrix_1.12.0         XML_3.99-0.18              
## [153] evaluate_1.0.3              BiocManager_1.30.25        
## [155] foreach_1.5.2               tzdb_0.4.0                 
## [157] httpuv_1.6.15               RANN_2.6.2                 
## [159] polyclip_1.10-7             future_1.34.0              
## [161] km.ci_0.5-6                 scattermore_1.2            
## [163] gridBase_0.4-7              rsvd_1.0.5                 
## [165] broom_1.0.7                 xtable_1.8-4               
## [167] restfulr_0.0.15             RSpectra_0.16-2            
## [169] rstatix_0.7.2               later_1.4.1                
## [171] ragg_1.3.3                  viridisLite_0.4.2          
## [173] GenomicAlignments_1.40.0    memoise_2.0.1              
## [175] AnnotationDbi_1.66.0        timechange_0.3.0           
## [177] globals_0.16.3              GSEABase_1.66.0
```
