# Supplementary material for: Developmental programmes drive cellular plasticity, disease progression and therapy resistance in lung adenocarcinoma
Source: Mol Oncol. 2026 May 27:10.1002/1878-0261.70263. Online ahead of print. doi: 10.1002/1878-0261.70263 (PMC13398952; doi:10.1002/1878-0261.70263)
Supplement: Supplementary file 1 — File 1. R Markdown HTML reports. [file MOL2-9999-0-s006.zip › Bienkowska_etal_MolOnc_Fig3.html]

Developmental programmes drive cellular plasticity, disease progression and therapy resistance in lung adenocarcinoma


# Developmental programmes drive cellular plasticity, disease progression and therapy resistance in lung adenocarcinoma

### FIGURE 3 - TP53 loss of function is required for BM activation

#### Kamila J Bienkowska, Stephany Gallardo Y, Nur S Zainal, Leena Arora, Matthew Ellis, Maria-Antoinette Lopez, Judith Austine, Sai Pittla, Serena J Chee, Aiman Alzetani, Emily C Shaw, Christian H Ottensmeier, Gareth J Thomas, Christopher J Hanley

#### 2025-08-27

```
library(decoupleR)
library(tidyverse)
library(GSVA)
library(ggpubr)
library(maftools)
library(TCGAmutations)
library(Seurat)
library(biomaRt)
library(ggplotify)
library(BSgenome.Hsapiens.UCSC.hg19)
library(NMF)
```

## Overview

Aim: To investigate the mechanisms that regulate ALV and BM
developmental programs in the progression of lung adenocarcinoma (LUAD)
using transcriptomic data and genetically engineered murine models
(GEMMs).

### 1. TCGA RNA-seq PROGENy analysis

In this script we will first utilise human RNA-seq data from TCGA to
identify pathways associated with BM, using decoupleR and the Pathway
RespOnsive GENes for activity inference (PROGENy) database.

```
setwd(input_files)
load(file="NSCLC_traits_all.Rdata")
load(file="NSCLC_vsd_all.Rdata")
load(file="Alveogenesis_signature_modified.Rdata")
load(file="Morphogenesis_signature_modified.Rdata")
TMB <- read.table(file = 'luad_tcga_clinical_data, TMB.tsv', sep = '\t', header = TRUE)
```

Calculate Dev signature ssGSEA scores

```
Dev.sig_list <- list(
  ALV = Alveogenesis_sig,
  BM = Morphogenesis_sig
)

ssGSEA_Dev_TCGA <- ssgseaParam(NSCLC_vsd_all, Dev.sig_list)
gsva.es <- gsva(ssGSEA_Dev_TCGA, verbose = T)

NSCLC_traits_all <- merge(NSCLC_traits_all, t(gsva.es), by = 0)
rownames(NSCLC_traits_all) <- NSCLC_traits_all$Row.names
NSCLC_traits_all <- NSCLC_traits_all[,-1]

#Subset data to LUAD samples only
LUAD_traits <- subset(NSCLC_traits_all, subset = Subtype == "LUAD")
```

Pathway RespOnsive GENes for activity inference (PROGENy)

```
net <- get_progeny(organism = 'human', top = 500)

sample_acts <- run_mlm(mat=NSCLC_vsd_all, net=net, .source='source', .target='target',
                       .mor='weight', minsize = 5)

sample_acts_mat <- sample_acts %>%
  pivot_wider(id_cols = 'condition', names_from = 'source',
              values_from = 'score') %>%
  column_to_rownames('condition') %>%
  as.matrix()

#Subset results to exclude LUSC and order by sample's BM score
sample_order <- NSCLC_traits_all %>% filter(!Subtype == "LUSC") %>% arrange(BM) %>% rownames()
sample_acts_mat.LUAD <- sample_acts_mat[sample_order, ]
```

Figure 3A - Plot Sample inference results

```
#Barplot showing pathway activity ranked by correlation to BM score
Figure_3A <- 
  data.frame(
  cor = cor(sample_acts_mat.LUAD, NSCLC_traits_all[sample_order, "BM"]),
  Pathway = rownames(cor(sample_acts_mat.LUAD, NSCLC_traits_all[sample_order, "BM"]))
) %>%
  ggplot(aes(x = reorder(Pathway, cor), y = cor, fill = cor)) +
  geom_bar(stat = "identity") +
  theme_pubr(base_size = 7) +
  rotate_x_text(angle = 45) +
  xlab("Pathway") + ylab("BM correlation (r)") +
  scale_fill_gradient2(high = scales::muted("red"), low = scales::muted("blue")) +
  theme(legend.position = "none")
Figure_3A
```

```
ggsave(Figure_3A, path = Plots_out, filename = "Figure_3A.svg",
       width = 9, height = 5, units = "cm")
```

Figure 3B - BM score vs. p53 pathway activation scatter plot

```
LUAD_sample.acts_df <-   data.frame(
  sample_acts_mat[sample_order, ],
  BM = LUAD_traits[sample_order, "BM"],
  ID = sample_order
)

Figure_3B <- 
  LUAD_sample.acts_df %>%
  ggplot(aes(y = BM, x = p53)) +
  theme_pubr(base_size = 7) +
  geom_point(size = 0.1) +
  stat_smooth(method = "lm") +
  stat_cor(label.x.npc = 0, label.y.npc = 0, size = 3) + xlab("p53 pathway activation\n (PROGENy score)") +
  ylab("BM (ssGSEA score)")
Figure_3B
```

```
ggsave(Figure_3B, path = Plots_out, filename = "Figure_3B.svg",
       width = 9, height = 5, units = "cm")
```

### 2. TCGA WGS analysis

Given the findings that p53 signalling is inversely correlated to BM,
we suspected that TP53 loss-of-function mutations may contribute to BM
activation. To test this we utilised WGS data from TCGA.

```
#based on LUAD TCGA surv cutpoint for 5year OS
LUAD_traits$Dev_prog <- NA
LUAD_traits$Dev_prog[LUAD_traits$BM > 0.1881233] <- "BM_high"
LUAD_traits$Dev_prog[LUAD_traits$BM < 0.1881233] <- "BM_low"

# create maf object by automatic download
LUAD_maf <- tcga_load(study = "LUAD", source = "MC3")
NGS.tsb.df <- merge(LUAD_traits, LUAD_maf@clinical.data[, 1:2], by.x = "Patient", by.y = "Tumor_Sample_Barcode_min")

BM.high_IDs <- NGS.tsb.df$Tumor_Sample_Barcode[NGS.tsb.df$Dev_prog == "BM_high"]
BM.low_IDs <- NGS.tsb.df$Tumor_Sample_Barcode[NGS.tsb.df$Dev_prog == "BM_low"]

BM.high_maf <- subsetMaf(LUAD_maf, tsb = BM.high_IDs)
BM.low_maf <- subsetMaf(LUAD_maf, tsb = BM.low_IDs)

# Compare 
BM.high.v.low_maf.res <- mafCompare(
  m1 = BM.high_maf, m2 = BM.low_maf, m1Name = "BM_high", m2Name = "BM_low", minMut = 5
)
oncoplot(maf = BM.high_maf, top = 15)
```

```
#oncoplot for BM high tumours (top mutated genes)
setwd(Plots_out)
png(file = "Figure_3C.png", units = "cm", res = 300, height = 15, width = 20)
oncoplot(maf = BM.high_maf, top = 15)
dev.off()
```

Figure 3D - Pathway WGS analysis in TCGA BM-high BM-Low

```
## odds ratio for pathways, looking at known oncogenic pathways from Sanchez/Vega et al
BM.high.v.low_maf.PW.res <- mafCompare(m1 = BM.high_maf, m2 = BM.low_maf, m1Name = "BM_high", m2Name = "BM_low", minMut = 5, pathways = "sigpw")
forestPlot(BM.high.v.low_maf.PW.res, fdr = 0.01)
```

```
setwd(Plots_out)
png(file = "Figure_3D.png", units = "cm", res = 300, height = 10, width = 15)
forestPlot(BM.high.v.low_maf.PW.res, fdr = 0.01)
dev.off()
```

Figure S3A - BM-low maf plot

```
#Supplementary plots
oncoplot(maf = BM.low_maf, top = 10)
```

```
# Oncoplot BM-low
setwd(Plots_out)
png(file = "Figure_S3A.png", units = "cm", res = 300, height = 10, width = 15)
oncoplot(maf = BM.low_maf, top = 10)
dev.off()
```

Figure S3B - TMB analysis by BM cat

```
colnames(TMB)[colnames(TMB) == "TMB..nonsynonymous."] = "TMB"
LUAD_traits$TMB <- TMB$TMB[match(LUAD_traits$Patient_Type, TMB$Sample.ID)]
LUAD_traits$Dev_prog <- factor(LUAD_traits$Dev_prog, levels = c("BM_low", "BM_high"))

Figure_S3B <- LUAD_traits %>% drop_na(TMB) %>%
  ggplot(aes(x = Dev_prog, y = TMB, fill = Dev_prog)) +
  theme_pubr(base_size = 7) +
  geom_jitter(alpha = 0.5, size = 0.1, width = 0.2) +
  geom_boxplot(outlier.shape = NA) +
  stat_pwc(label = "p.adj.signif", p.adjust.method = "fdr", fontsize = 2, hide.ns = T, vjust = 0.5, tip.length = 0)+
  scale_fill_manual("LUAD", values = c("#794DFF", "#FF6341")) +
  theme(axis.title.x = element_blank(), legend.position = "none") +
  ylab("Tumour mutational burden\n(mut/Mb)")
Figure_S3B
```

```
ggsave(Figure_S3B, path = Plots_out, filename = "Figure_S3B.svg",
       width = 4, height = 4, units = "cm")
```

Figure S3C - SBS signatures by BM cat

```
# tobacco smoking sig
laml.tnm.BM = trinucleotideMatrix(maf = BM.high_maf, prefix = 'chr', add = TRUE)
laml.sig.BM = extractSignatures(mat = laml.tnm.BM, n = 3)
laml.tnm.ALV = trinucleotideMatrix(maf = BM.low_maf, prefix = 'chr', add = TRUE)
laml.sig.ALV = extractSignatures(mat = laml.tnm.ALV, n = 3)
contr_ALV <- laml.sig.ALV$contributions
contr_BM <- laml.sig.BM$contributions

maftools::plotSignatures(nmfRes = laml.sig.BM, title_size = 1.2, sig_db = "SBS")
```

```
maftools::plotSignatures(nmfRes = laml.sig.ALV, title_size = 1.2, sig_db = "SBS")
```

```
rownames(contr_ALV) <- c("APOBEC", "DNA_mismatch_repair","Tobacco_smoking")
rownames(contr_BM) <- c("APOBEC", "DNA_mismatch_repair","Tobacco_smoking")

# compare contributions
contr_ALV <- t(contr_ALV)
contr_ALV <- as.data.frame(contr_ALV)
contr_ALV$dev_programme <- NA
contr_ALV$dev_programme <- "BM_low"

contr_BM <- t(contr_BM)
contr_BM <- as.data.frame(contr_BM)

contr_BM$dev_programme <- NA
contr_BM$dev_programme <- "BM_high"

contr <- rbind(contr_BM, contr_ALV)

LUAD_traits$APOBEC <- contr$APOBEC[match(LUAD_traits$Patient, rownames(contr))]
LUAD_traits$DNA_mismatch_repair <- contr$DNA_mismatch_repair[match(LUAD_traits$Patient, rownames(contr))]
LUAD_traits$Tobacco_smoking <- contr$Tobacco_smoking[match(LUAD_traits$Patient, rownames(contr))]

contr$dev_programme <- factor(contr$dev_programme, levels = c("BM_low", "BM_high"))
palette <- c("#794DFF", "#FF6341")

Figure_S3C <- 
  contr %>% ggplot(aes(x = dev_programme, y = Tobacco_smoking,
                fill = dev_programme)) +
  theme_pubr(base_size = 7) +
  geom_jitter(alpha = 0.5, size = 0.1, width = 0.2) +
  geom_boxplot(outlier.shape = NA) +
  stat_pwc(label = "p.adj.signif", p.adjust.method = "fdr", fontsize = 2, hide.ns = T, vjust = 0.5, tip.length = 0)+
  scale_fill_manual(values = palette) +
  theme(axis.title.x = element_blank(), legend.position = "none")  + ylab("Tobacco smoking\n(SBS mutational signature)")

Figure_S3C
```

```
ggsave(Figure_S3C, path = Plots_out, filename = "Figure_S3C.svg",
       width = 4, height = 4, units = "cm")
```

Figure S3 D&E - Analysis of BM levels in the context of TP53,
KRAS and EGFR mutations

```
# KRAS,TP53, EGFR in LUAD
setwd(input_files)
oncoplot(maf = LUAD_maf, top = 10, genes = c("TP53", "KRAS", "EGFR"), writeMatrix = T)
```

```
matrix <- read.delim(file="onco_matrix.txt", header = T, sep = '\t', na.strings = c("", "NA"))
matrix <- t(matrix)
matrix <- as.data.frame(matrix)

# remove samples with all zeros (data not available)
all_char_zeros_rows <- apply(matrix, 1, function(row) {
  all(sapply(row, function(x) is.character(x) && x == "0"))
})
matrix <- matrix[!all_char_zeros_rows, ]

matrix$KRAS_only[!(is.na(matrix$KRAS)) & is.na(matrix$TP53) & is.na(matrix$EGFR)] <- "KRAS_only"
matrix$TP53_only[!(is.na(matrix$TP53)) & is.na(matrix$KRAS) & is.na(matrix$EGFR)] <- "TP53_only"
matrix$EGFR_only[!(is.na(matrix$EGFR)) & is.na(matrix$KRAS) & is.na(matrix$TP53)] <- "EGFR_only"
matrix$TP53_KRAS[!(is.na(matrix$TP53)) & !is.na(matrix$KRAS) & is.na(matrix$EGFR)] <- "TP53/KRAS"
matrix$TP53_EGFR[!(is.na(matrix$TP53)) & is.na(matrix$KRAS) & !is.na(matrix$EGFR)] <- "TP53/EGFR"
# only one sample with all three mutations, not including, no samples with both KRAS/EGFR (and not TP53)


# extract sample names
KRAS_samples <- subset(matrix, KRAS_only == "KRAS_only")
KRAS_samples <- rownames(KRAS_samples)
TP53_samples <- subset(matrix, TP53_only == "TP53_only")
TP53_samples <- rownames(TP53_samples)
EGFR_samples <- subset(matrix, EGFR_only == "EGFR_only")
EGFR_samples <- rownames(EGFR_samples)
KRAS_TP53_samples <- subset(matrix, TP53_KRAS == "TP53/KRAS")
KRAS_TP53_samples <- rownames(KRAS_TP53_samples)
EGFR_TP53_samples <- subset(matrix, TP53_EGFR == "TP53/EGFR")
EGFR_TP53_samples <- rownames(EGFR_TP53_samples)

LUAD_traits$Tumour_sample_barcode <- NA
LUAD_traits$Tumour_sample_barcode <- NGS.tsb.df$Tumor_Sample_Barcode[match(LUAD_traits$Patient_Type,
                                                                           NGS.tsb.df$Patient_Type)]
TP53_samples <- gsub('\\.', '-', TP53_samples)
LUAD_traits$mutation[LUAD_traits$Tumour_sample_barcode %in% TP53_samples] <- "TP53"

KRAS_samples <- gsub('\\.', '-', KRAS_samples)
LUAD_traits$mutation[LUAD_traits$Tumour_sample_barcode %in% KRAS_samples] <- "KRAS"

EGFR_samples <- gsub('\\.', '-', EGFR_samples)
LUAD_traits$mutation[LUAD_traits$Tumour_sample_barcode %in% EGFR_samples] <- "EGFR"

KRAS_TP53_samples <- gsub('\\.', '-', KRAS_TP53_samples)
LUAD_traits$mutation[LUAD_traits$Tumour_sample_barcode %in% KRAS_TP53_samples] <- "KRAS/TP53"

EGFR_TP53_samples <- gsub('\\.', '-', EGFR_TP53_samples)
LUAD_traits$mutation[LUAD_traits$Tumour_sample_barcode %in% EGFR_TP53_samples] <- "EGFR/TP53"

# Replace NA values with WT 
LUAD_traits$mutation[is.na(LUAD_traits$mutation)] <- "WT"

# remove samples without the tumour barcode 
LUAD_traits <- LUAD_traits[!is.na(LUAD_traits$Tumour_sample_barcode), ]

LUAD_traits$mutation <- factor(LUAD_traits$mutation,
                               levels = c("WT", "TP53", "EGFR","EGFR/TP53", "KRAS",  "KRAS/TP53"),
                               labels = c("EGFR-WT | TP53-WT | KRAS-WT",
                                          "TP53-mut | KRAS/EGFR-WT",
                                          "EGFR-mut | KRAS/TP53-WT",
                                          "EGFR-mut/TP53-mut | KRAS-WT",
                                          "KRAS-mut | EGFR/TP53-WT",
                                          "KRAS-mut/TP53-mut | EGFR-WT"))

# histogram
Figure_S3D <- gghistogram(
  LUAD_traits, x = "BM",
  add = "median", rug = TRUE, fill = "mutation", add_density = TRUE, alpha = 1, size = 1
) +
  theme_pubr(base_size = 7) +
  theme(legend.key.size = unit(2,"pt"), legend.position = "right") +
  scale_fill_brewer(palette = "Paired") +
  scale_colour_brewer(palette = "Paired") +
  ylab("density")

Figure_S3D
```

```
ggsave(Figure_S3D, path = Plots_out, filename = "Figure_S3D.svg",
       width = 10, height = 6, units = "cm")
```

BM levels by TP53, KRAS and EGFR - Boxplots

```
# remove samples with all zeros (data not available)
setwd(input_files)
matrix <- read.delim(file="onco_matrix.txt", header = T, sep = '\t', na.strings = c("", "NA"))
matrix <- t(matrix)
matrix <- as.data.frame(matrix)

# remove samples with all zeros (data not available)
all_char_zeros_rows <- apply(matrix, 1, function(row) {
  all(sapply(row, function(x) is.character(x) && x == "0"))
})
matrix <- matrix[!all_char_zeros_rows, ]

matrix <- 
  matrix %>%  mutate(
    TP53_altered = !is.na(TP53),
    KRAS_altered = !is.na(KRAS),
    EGFR_altered = !is.na(EGFR)
  )

table(substr(rownames(matrix), 0,16) %in% substr(rownames(LUAD_traits), 0,16))
plot_df <- LUAD_traits
plot_df$ID.match <- substr(rownames(plot_df), 0,16)
matrix$ID.match <- substr(rownames(matrix), 0,16)

plot_df <- merge(plot_df, matrix, by = "ID.match", all.x = T)

Figure_S3Ei <- plot_df %>% filter(!is.na(TP53_altered)) %>%
  ggplot(aes(y = BM , x = TP53_altered, fill = TP53_altered)) +
  theme_pubr(base_size = 7) +
  geom_jitter(alpha = 0.5, size = 0.1, width = 0.2) +
  geom_boxplot(outlier.shape = NA) +
  stat_pwc(label = "p.adj.signif", p.adjust.method = "fdr", fontsize = 2, hide.ns = T, vjust = 0.5, tip.length = 0)+
  ylab("BM (ssGSEA score)") +
  ggtitle("All cases") +
  theme(axis.text.x = element_blank(), axis.title.x = element_blank()) +
  scale_y_continuous(expand = expansion(mult = 0.2)) +
  scale_fill_manual(values = c("#794DFF", "#FF6341"))

Figure_S3Eii <- plot_df %>% filter(!is.na(TP53_altered)) %>%
  filter(KRAS_altered == T) %>%
  ggplot(aes(y = BM , x = TP53_altered, fill = TP53_altered)) +
  theme_pubr(base_size = 7) +
  geom_jitter(alpha = 0.5, size = 0.1, width = 0.2) +
  geom_boxplot(outlier.shape = NA) +
  stat_pwc(label = "p.adj.signif", p.adjust.method = "fdr", fontsize = 2, hide.ns = T, vjust = 0.5, tip.length = 0)+
  ylab("BM (ssGSEA score)") +
  ggtitle("KRAS mutant") +
  theme(axis.text.x = element_blank(), axis.title.x = element_blank()) +
  scale_y_continuous(expand = expansion(mult = 0.2)) +
  scale_fill_manual(values = c("#794DFF", "#FF6341"))

Figure_S3Eiii <- plot_df %>% filter(!is.na(TP53_altered)) %>%
  filter(EGFR_altered == T) %>%
  ggplot(aes(y = BM , x = TP53_altered, fill = TP53_altered)) +
  theme_pubr(base_size = 7) +
  geom_jitter(alpha = 0.5, size = 0.1, width = 0.2) +
  geom_boxplot(outlier.shape = NA) +
  stat_pwc(label = "p.adj.signif", p.adjust.method = "fdr", fontsize = 2, hide.ns = T, vjust = 0.5, tip.length = 0)+
  ylab("BM (ssGSEA score)") +
  ggtitle("EGFR mutant") +
  theme(axis.text.x = element_blank(), axis.title.x = element_blank()) +
  scale_y_continuous(expand = expansion(mult = 0.2)) +
  scale_fill_manual(values = c("#794DFF", "#FF6341"))

Figure_S3E <- ggarrange(Figure_S3Ei,Figure_S3Eii,Figure_S3Eiii, ncol = 3, common.legend = T, legend = "bottom")
Figure_S3E
```

```
ggsave(Figure_S3E, path = Plots_out, filename = "Figure_S3E.svg",
       width = 8, height = 5, units = "cm")
```

### 3. TP53 LOF in K and KP LUAD GEMM scRNA-seq data

To investigate whether TP53 loss directly contributes to BM
activation in LUAD, we used scRNA-seq data (GSE154989), from murine AT2
cells at varying stages of oncogenesis (hyperplasia, adenoma and LUAD)
induced by either Kras-G12D over-expression alone (K) or simultaneous
loss of Trp53 (KP), generated by Marjanovic ND et al [@10.1016/j.ccell.2020.06.012].

For this analysis we first load and process the data from the NCBI
GEO database and supplementary information from the Marjanovic ND et al
publication. We then examined the expression of ALV and BM developmental
programs as module scores in the GEMMs to compare their expression level
across genotype and disease stage.

```
## demo code not run
file.h5 <- H5File$new(filename = "GSE154989_mmLungPlate_fQC_dSp_rawCount.h5", mode = "r")

file.h5$ls()
i = file.h5[["i"]]
j = file.h5[["j"]]
v = file.h5[["v"]]
max_i <- max(i$read())
max_j <- max(j$read())

gene_names <- read.csv("GSE154989_mmLungPlate_fQC_geneTable.csv.gz")
sample_names <- read.csv("GSE154989_mmLungPlate_fQC_smpTable.csv.gz")
sample_annot <- read.csv("GSE154989_mmLungPlate_fQC_dZ_annot_smpTable.csv.gz")
sample_QC <- read.csv("GSE154989_mmLungPlate_fQC_dZ_QCstat_smpTable.csv.gz")
sample_MetaData <- merge(sample_annot, sample_QC, by = "sampleID")
rownames(sample_MetaData) <- sample_MetaData$sampleID
sample_MetaData <- cbind(str_split_fixed(sample_MetaData$sampleID, "_", 7), sample_MetaData)
names(sample_MetaData)[1:7] <- c("Genotype", "Timepoint", "Treatment", "Mouse", "Tumour", "Plate", "Single_cell_index")

feature_MetaData <- gene_names
rownames(feature_MetaData) <- feature_MetaData$ensgID

matSparse <- sparseMatrix(
  i = i$read(),
  j = j$read(), 
  x = v$read(), 
  dims = c(max_i, max_j)
)
dim(matSparse)
rownames(matSparse) <- gene_names$geneID
colnames(matSparse) <- sample_names$sampleID

file.h5$close_all()

save(matSparse, sample_MetaData, feature_MetaData, file = "GSE154989_RAW.Rdata")
```

First we create a Seurat object using the raw counts from the
Marjanovic et al. study and aggregate to sample pseudobulk

```
GSE154989_scRNAeq.10X <- CreateSeuratObject(matSparse,
                                            meta.data = sample_MetaData)
GSE154989_scRNAeq.10X[["RNA"]][[]] <- feature_MetaData
GSE154989_scRNAeq.10X@assays$RNA@meta.data$geneID <- gsub("_", "-", GSE154989_scRNAeq.10X@assays$RNA@meta.data$geneID, fixed = T)


mito_genes <- gsub("_", "-",feature_MetaData$geneID[grep("^mt-", feature_MetaData$geneSymbol)], fixed = T)
GSE154989_scRNAeq.10X[["percent.mt"]] <- PercentageFeatureSet(GSE154989_scRNAeq.10X, features = mito_genes)
GSE154989_scRNAeq.10X <- NormalizeData(GSE154989_scRNAeq.10X, normalization.method = "LogNormalize", scale.factor = 10000)

GSE154989_scRNAeq.10X$Genotype_Timepoint <- factor(paste(GSE154989_scRNAeq.10X$Genotype, GSE154989_scRNAeq.10X$Timepoint),
                                                   levels = c("T 0w", "T 4w", "K 2w", "KP 2w", "K 12w", "K 30w", "KP 12w", "KP 18w", "KP 20w", "KP 30w"))

GSE154989_scRNAeq.10X$Histo.Grade <- factor(GSE154989_scRNAeq.10X$Genotype_Timepoint,                                                   levels = c("T 0w", "T 4w", "K 2w", "KP 2w", "K 12w", "K 30w", "KP 12w", "KP 18w", "KP 20w", "KP 30w"),
                                      labels = c("Normal", "Normal", "AAH", "AAH", "Adenoma", "Adenoma", "Adenoma", "LUAD", "LUAD", "LUAD"))

GSE154989_scRNAeq.10X$nWeeks <- as.numeric(gsub("w", "", GSE154989_scRNAeq.10X$Timepoint, fixed = T))

GSE154989_scRNAeq.10X_PB <- AggregateExpression(GSE154989_scRNAeq.10X, return.seurat = T, group.by = c("Genotype_Timepoint", "Mouse", "Tumour", "Timepoint", "Histo.Grade"))

GSE154989_scRNAeq.10X_PB$Genotype_Timepoint <- factor(GSE154989_scRNAeq.10X_PB$Genotype_Timepoint, 
                                                      levels = c("T 0w", "T 4w", "K 2w", "KP 2w", "K 12w", "K 30w", "KP 12w", "KP 18w", "KP 20w", "KP 30w"))
GSE154989_scRNAeq.10X_PB$nWeeks <- as.numeric(gsub("w", "", GSE154989_scRNAeq.10X_PB$Timepoint, fixed = T))
```

Given that the ALV/BM signatures used in our analysis were derived
from mice we will work with the original gene lists and murine genes
rather than human homologues/orthologs.

Calculate Module scores for each program.

```
setwd(input_files)
Dev.sigs_df <- readxl::read_xlsx("pnas.1311760110_sd01.xlsx")

Dev.sigs_list <- list(
  ALV = Dev.sigs_df$SYMBOL[1:100],
  BM = Dev.sigs_df$SYMBOL[(nrow(Dev.sigs_df)-99):nrow(Dev.sigs_df)]
)


ensembl <- useEnsembl(biomart = "ENSEMBL_MART_MOUSE", 
                      dataset = "mmc57bl6nj_gene_ensembl", 
                      mirror = "useast")
proliferation.go <- getBM(attributes=c('external_gene_name', 'ensembl_gene_id', 'go_id'),
                          filters = 'go', values = c('GO:0008283', "GO:0007049", "GO:0051301", "GO:0006260"), mart = ensembl)
length(unique(proliferation.go$external_gene_name))
table(Dev.sigs_list$BM %in% proliferation.go$external_gene_name )

Dev.sigs_list$BM_CC = Dev.sigs_list$BM[Dev.sigs_list$BM %in% proliferation.go$external_gene_name]
Dev.sigs_list$BM_nonCC = Dev.sigs_list$BM[!Dev.sigs_list$BM %in% proliferation.go$external_gene_name]


Dev.sigs_list.geneIDs <- list()
for(i in names(Dev.sigs_list)){
  Dev.sigs_list.geneIDs[[i]] <- gsub("_", "-", feature_MetaData$geneID[feature_MetaData$geneSymbol %in% Dev.sigs_list[[i]]])
}


GSE154989_scRNAeq.10X_PB <- AddModuleScore(GSE154989_scRNAeq.10X_PB, features = list(Dev.sigs_list.geneIDs$ALV), name = "ALV")
GSE154989_scRNAeq.10X_PB <- AddModuleScore(GSE154989_scRNAeq.10X_PB, features = list(Dev.sigs_list.geneIDs$BM), name = "BM")
GSE154989_scRNAeq.10X_PB <- AddModuleScore(GSE154989_scRNAeq.10X_PB, features = list(Dev.sigs_list.geneIDs$BM_nonCC), name = "BM_nonCC")
GSE154989_scRNAeq.10X_PB <- AddModuleScore(GSE154989_scRNAeq.10X_PB, features = list(Dev.sigs_list.geneIDs$BM_CC), name = "BM_CC")

GSE154989_scRNAeq.10X_PB$Genotype <- factor(str_split_fixed(GSE154989_scRNAeq.10X_PB$Genotype_Timepoint, " ", 2)[,1], levels = c("T", "K", "KP"))
GSE154989_scRNAeq.10X_PB$Genotype.detailed <- factor(GSE154989_scRNAeq.10X_PB$Genotype, levels = c("T", "K", "KP"), labels = c("KRAS-WT\nTP53-WT", "KRAS-G12D\nTP53-WT", "KRAS-G12D\nTP53-NULL"))
```

Figure 3F and S3G - Boxplots comparing BM and ALV levels by
genotype

```
KP_model_pal <- RColorBrewer::brewer.pal(3, "Set2")
names(KP_model_pal) <- levels(GSE154989_scRNAeq.10X_PB$Genotype.detailed)


Figure_3Fi <- GSE154989_scRNAeq.10X_PB@meta.data %>%
    filter(!Histo.Grade %in% c("AAH")) %>%
    ggplot(aes(y = ALV1, x = Genotype.detailed, fill = Genotype.detailed)) +
    theme_pubr(base_size = 7) + theme(axis.title.x = element_blank())+
    geom_boxplot(outlier.shape = NA) +
    geom_jitter(size = 0.5, width = 0.2) +
    rotate_x_text(angle = 45) +  
    ylab("ALV Module Score\n(Aggregated over single-cells)") + xlab("Genotype") +
    stat_pwc(label = "p.adj.signif", p.adjust.method = "fdr", fontsize = 2, hide.ns = T, vjust = 0.5, tip.length = 0)+
    scale_fill_manual(values = KP_model_pal) +
    scale_y_continuous(expand = expansion(mult = 0.1)) +
    theme(legend.position = "none")

Figure_3Fii <- GSE154989_scRNAeq.10X_PB@meta.data %>%
    filter(!Histo.Grade %in% c("AAH")) %>%
    ggplot(aes(y = BM1, x = Genotype.detailed, fill = Genotype.detailed)) +
    theme_pubr(base_size = 7) + theme(axis.title.x = element_blank())+
    geom_boxplot(outlier.shape = NA) +
    geom_jitter(size = 0.5, width = 0.2) +
    rotate_x_text(angle = 45) +
    ylab("BM Module Score\n(Aggregated over single-cells)") + xlab("Genotype") +
    stat_pwc(label = "p.adj.signif", p.adjust.method = "fdr", fontsize = 2, hide.ns = T, vjust = 0.5, tip.length = 0)+
    scale_fill_manual(values = KP_model_pal) +
    scale_y_continuous(expand = expansion(mult = 0.1)) + theme(legend.position = "none")

Figure_3F <- ggarrange(
  Figure_3Fi, Figure_3Fii,
  ncol = 2, legend = "none"
)
Figure_3F
```

```
ggsave(Figure_3F, path = Plots_out, file = "Figure_3f.svg",
       width = 8, height = 5, units = "cm")

# Comparison at specific time-points
Figure_S3F <- 
 GSE154989_scRNAeq.10X_PB@meta.data %>%
    filter(!Genotype == "T" & Timepoint %in% c("12w", "30w")) %>%
    ggplot(aes(y = ALV1, x = Genotype.detailed, fill = Genotype.detailed)) +
    theme_pubr(base_size = 7) + theme(axis.title.x = element_blank())+
    geom_boxplot(outlier.shape = NA) +
    geom_jitter(size = 0.5, width = 0.2) +
    scale_fill_manual(values = KP_model_pal) +
    rotate_x_text(angle = 45) +
    ylab("ALV Module Score\n(Aggregated over single-cells)") + xlab("Genotype") +
    stat_pwc(label = "p.adj.signif", p.adjust.method = "fdr", fontsize = 2, hide.ns = T, vjust = 0.5, tip.length = 0, bracket.nudge.y = -0.04)+
    theme(legend.position = "none") +
    facet_wrap(~Timepoint)
Figure_S3F
```

```
ggsave(Figure_S3F, path = Plots_out, file = "Figure_S3F.svg",
       width = 8, height = 5.5, units = "cm")

Figure_S3G <- 
  GSE154989_scRNAeq.10X_PB@meta.data %>%
    filter(!Genotype == "T" & Timepoint %in% c("12w", "30w")) %>%
    ggplot(aes(y = BM1, x = Genotype.detailed, fill = Genotype.detailed)) +
    theme_pubr(base_size = 7) + theme(axis.title.x = element_blank())+
    geom_boxplot(outlier.shape = NA) +
    geom_jitter(size = 0.5, width = 0.2) +
    scale_fill_manual(values = KP_model_pal) +
    rotate_x_text(angle = 45) +
    ylab("BM Module Score\n(Aggregated over single-cells)") + xlab("Genotype") +
    stat_pwc(label = "p.adj.signif", p.adjust.method = "fdr", fontsize = 2, hide.ns = T, vjust = 0.5, tip.length = 0, bracket.nudge.y = -0.04)+
    theme(legend.position = "none") +
    facet_wrap(~Timepoint) 
Figure_S3G
```

```
ggsave(Figure_S3G, path = Plots_out, file = "Figure_S3G.svg",
       width = 8, height = 5.5, units = "cm")
```

### 4. Session info

```
print(sessionInfo(), RNG = TRUE, locale = FALSE)
```

```
## R version 4.4.0 (2024-04-24 ucrt)
## Platform: x86_64-w64-mingw32/x64
## Running under: Windows 11 x64 (build 26100)
## 
## Matrix products: default
## 
## 
## Random number generation:
##  RNG:     Mersenne-Twister 
##  Normal:  Inversion 
##  Sample:  Rejection 
##  
## attached base packages:
## [1] stats4    stats     graphics  grDevices utils     datasets  methods  
## [8] base     
## 
## other attached packages:
##  [1] NMF_0.28                          Biobase_2.64.0                   
##  [3] cluster_2.1.6                     rngtools_1.5.2                   
##  [5] registry_0.5-1                    BSgenome.Hsapiens.UCSC.hg19_1.4.3
##  [7] BSgenome_1.72.0                   rtracklayer_1.64.0               
##  [9] BiocIO_1.14.0                     Biostrings_2.72.1                
## [11] XVector_0.44.0                    GenomicRanges_1.56.2             
## [13] GenomeInfoDb_1.40.1               IRanges_2.38.1                   
## [15] S4Vectors_0.42.1                  BiocGenerics_0.50.0              
## [17] ggplotify_0.1.2                   biomaRt_2.60.1                   
## [19] Seurat_5.2.0                      SeuratObject_5.0.2               
## [21] sp_2.1-4                          TCGAmutations_0.4.0              
## [23] data.table_1.16.0                 maftools_2.20.0                  
## [25] ggpubr_0.6.0                      GSVA_1.52.3                      
## [27] lubridate_1.9.3                   forcats_1.0.0                    
## [29] stringr_1.5.1                     dplyr_1.1.4                      
## [31] purrr_1.0.2                       readr_2.1.5                      
## [33] tidyr_1.3.1                       tibble_3.2.1                     
## [35] ggplot2_3.5.1                     tidyverse_2.0.0                  
## [37] decoupleR_2.10.0                 
## 
## loaded via a namespace (and not attached):
##   [1] bitops_1.0-9                fs_1.6.4                   
##   [3] matrixStats_1.4.1           spatstat.sparse_3.1-0      
##   [5] doParallel_1.0.17           httr_1.4.7                 
##   [7] RColorBrewer_1.1-3          tools_4.4.0                
##   [9] sctransform_0.4.1           backports_1.5.0            
##  [11] R6_2.5.1                    HDF5Array_1.32.1           
##  [13] mgcv_1.9-1                  lazyeval_0.2.2             
##  [15] uwot_0.2.2                  rhdf5filters_1.16.0        
##  [17] withr_3.0.2                 prettyunits_1.2.0          
##  [19] gridExtra_2.3               progressr_0.15.1           
##  [21] textshaping_0.4.1           cli_3.6.3                  
##  [23] spatstat.explore_3.3-4      fastDummies_1.7.4          
##  [25] labeling_0.4.3              sass_0.4.9                 
##  [27] spatstat.data_3.1-4         ggridges_0.5.6             
##  [29] pbapply_1.7-2               systemfonts_1.1.0          
##  [31] Rsamtools_2.20.0            yulab.utils_0.1.9          
##  [33] svglite_2.1.3               R.utils_2.12.3             
##  [35] sessioninfo_1.2.2           parallelly_1.41.0          
##  [37] readxl_1.4.3                rstudioapi_0.17.1          
##  [39] RSQLite_2.3.7               generics_0.1.3             
##  [41] gridGraphics_0.5-1          vroom_1.6.5                
##  [43] ica_1.0-3                   spatstat.random_3.3-2      
##  [45] zip_2.3.1                   car_3.1-3                  
##  [47] Matrix_1.7-0                logger_0.4.0               
##  [49] abind_1.4-8                 R.methodsS3_1.8.2          
##  [51] lifecycle_1.0.4             yaml_2.3.10                
##  [53] carData_3.0-5               SummarizedExperiment_1.34.0
##  [55] rhdf5_2.48.0                SparseArray_1.4.8          
##  [57] BiocFileCache_2.12.0        Rtsne_0.17                 
##  [59] grid_4.4.0                  blob_1.2.4                 
##  [61] promises_1.3.2              crayon_1.5.3               
##  [63] miniUI_0.1.1.1              lattice_0.22-6             
##  [65] beachmat_2.20.0             cowplot_1.1.3              
##  [67] annotate_1.82.0             KEGGREST_1.44.1            
##  [69] magick_2.8.5                pillar_1.10.1              
##  [71] knitr_1.49                  tcltk_4.4.0                
##  [73] rjson_0.2.23                future.apply_1.11.3        
##  [75] codetools_0.2-20            glue_1.8.0                 
##  [77] spatstat.univar_3.1-1       vctrs_0.6.5                
##  [79] png_0.1-8                   spam_2.11-0                
##  [81] cellranger_1.1.0            gtable_0.3.6               
##  [83] cachem_1.1.0                OmnipathR_3.15.9           
##  [85] xfun_0.50                   S4Arrays_1.4.1             
##  [87] mime_0.12                   survival_3.8-3             
##  [89] SingleCellExperiment_1.26.0 iterators_1.0.14           
##  [91] fitdistrplus_1.2-2          ROCR_1.0-11                
##  [93] nlme_3.1-166                bit64_4.5.2                
##  [95] progress_1.2.3              filelock_1.0.3             
##  [97] RcppAnnoy_0.0.22            bslib_0.8.0                
##  [99] irlba_2.3.5.1               KernSmooth_2.23-26         
## [101] colorspace_2.1-1            DBI_1.2.3                  
## [103] DNAcopy_1.78.0              tidyselect_1.2.1           
## [105] bit_4.5.0                   compiler_4.4.0             
## [107] curl_6.1.0                  rvest_1.0.4                
## [109] httr2_1.0.7                 graph_1.82.0               
## [111] xml2_1.3.6                  DelayedArray_0.30.1        
## [113] plotly_4.10.4               checkmate_2.3.2            
## [115] scales_1.3.0                lmtest_0.9-40              
## [117] rappdirs_0.3.3              SpatialExperiment_1.14.0   
## [119] digest_0.6.37               goftest_1.2-3              
## [121] spatstat.utils_3.1-2        rmarkdown_2.29             
## [123] htmltools_0.5.8.1           pkgconfig_2.0.3            
## [125] sparseMatrixStats_1.16.0    MatrixGenerics_1.16.0      
## [127] dbplyr_2.5.0                fastmap_1.2.0              
## [129] rlang_1.1.4                 htmlwidgets_1.6.4          
## [131] UCSC.utils_1.0.0            shiny_1.10.0               
## [133] farver_2.1.2                jquerylib_0.1.4            
## [135] zoo_1.8-12                  jsonlite_1.8.9             
## [137] BiocParallel_1.38.0         R.oo_1.27.0                
## [139] RCurl_1.98-1.16             BiocSingular_1.20.0        
## [141] magrittr_2.0.3              Formula_1.2-5              
## [143] GenomeInfoDbData_1.2.12     dotCall64_1.2              
## [145] patchwork_1.3.0             Rhdf5lib_1.26.0            
## [147] munsell_0.5.1               Rcpp_1.0.13                
## [149] reticulate_1.40.0           stringi_1.8.4              
## [151] zlibbioc_1.50.0             MASS_7.3-61                
## [153] plyr_1.8.9                  parallel_4.4.0             
## [155] listenv_0.9.1               ggrepel_0.9.6              
## [157] deldir_2.0-4                splines_4.4.0              
## [159] tensor_1.5                  hms_1.1.3                  
## [161] igraph_2.0.3                spatstat.geom_3.3-4        
## [163] ggsignif_0.6.4              RcppHNSW_0.6.0             
## [165] reshape2_1.4.4              ScaledMatrix_1.12.0        
## [167] XML_3.99-0.18               evaluate_1.0.3             
## [169] BiocManager_1.30.25         selectr_0.4-2              
## [171] foreach_1.5.2               tzdb_0.4.0                 
## [173] httpuv_1.6.15               RANN_2.6.2                 
## [175] polyclip_1.10-7             future_1.34.0              
## [177] scattermore_1.2             gridBase_0.4-7             
## [179] rsvd_1.0.5                  broom_1.0.7                
## [181] xtable_1.8-4                restfulr_0.0.15            
## [183] RSpectra_0.16-2             rstatix_0.7.2              
## [185] later_1.4.1                 ragg_1.3.3                 
## [187] viridisLite_0.4.2           GenomicAlignments_1.40.0   
## [189] memoise_2.0.1               AnnotationDbi_1.66.0       
## [191] timechange_0.3.0            globals_0.16.3             
## [193] GSEABase_1.66.0
```
