# Supplementary material for: Developmental programmes drive cellular plasticity, disease progression and therapy resistance in lung adenocarcinoma
Source: Mol Oncol. 2026 May 27:10.1002/1878-0261.70263. Online ahead of print. doi: 10.1002/1878-0261.70263 (PMC13398952; doi:10.1002/1878-0261.70263)
Supplement: Supplementary file 1 — File 1. R Markdown HTML reports. [file MOL2-9999-0-s006.zip › Bienkowska_etal_MolOnc_Fig4.html]

Developmental programmes drive cellular plasticity, disease progression and therapy resistance in lung adenocarcinoma


# Developmental programmes drive cellular plasticity, disease progression and therapy resistance in lung adenocarcinoma

### Figure 4 - BM activation is associated with targeted-therapy resistance in lung adenocarcinomas

#### Kamila J Bienkowska, Stephany Gallardo Y, Nur S Zainal, Leena Arora, Matthew Ellis, Maria-Antoinette Lopez, Judith Austine, Sai Pittla, Serena J Chee, Aiman Alzetani, Emily C Shaw, Christian H Ottensmeier, Gareth J Thomas, Christopher J Hanley

#### 2025-11-25

Load packages

```
library(ggplot2)
library(ggpubr)
library(Seurat)
library(GSVA)
library(dplyr)
library(readr)
library(stringr)
library(survival)
library(survminer)
library(ggsci)
library(tidyr)
library(forestmodel)
```

Load objects

```
setwd(input_files)
load("Maynard_Epi_pseudobulk.Rdata") 
load("Maynard_Epi_seurat.Rdata")
load(file="Alveogenesis_signature_modified.Rdata")
load(file="Morphogenesis_signature_modified.Rdata")
load(file="NSCLC_TKI_2021.Rdata")
load(file="GSE135222_ICB.Rdata")
load(file="GSE207422_ICB.Rdata")
load(file="GSE207422_seurat.Rdata")
PDL1 <- read.delim(file = 'CD274_RPPA.txt', sep = '\t', header = TRUE)
load(file="NSCLC_traits_all.Rdata")
load(file="NSCLC_vsd_all.Rdata")
```

Figure 4B - Maynard et al. pseudobulk

```
Dev.sig_list <- list(
  ALV = Alveogenesis_sig,
  BM = Morphogenesis_sig
)

ssGSEA_Maynard.Epi_pseudobulk <- GSVA::ssgseaParam(expr=as.matrix(Maynard_Epi_pseudobulk@assays$RNA@data),
                                     Dev.sig_list)
ssGSEA_Maynard.Epi_pseudobulk <- gsva(ssGSEA_Maynard.Epi_pseudobulk, verbose = T)

rownames(Maynard_Epi_pseudobulk@meta.data) <- Maynard_Epi_pseudobulk@meta.data$Row.names
Maynard_Epi_pseudobulk@meta.data <- merge(Maynard_Epi_pseudobulk@meta.data, t(ssGSEA_Maynard.Epi_pseudobulk), by = 0)
rownames(Maynard_Epi_pseudobulk@meta.data) <- Maynard_Epi_pseudobulk@meta.data$Row.names
Maynard_Epi_pseudobulk@meta.data <- Maynard_Epi_pseudobulk@meta.data[,-c(1,2)]


Maynard_Epi_pseudobulk$FirstlineTKI <- 
  Maynard_Epi_pseudobulk$treatment_history_detail %in% c("on firstline TKI", "on firsline TKI")
Maynard_Epi_pseudobulk$analysis2 <- factor(Maynard_Epi_pseudobulk$analysis,
                                           levels = c("naive", "grouped_pr", "grouped_pd"),
                                           labels = c("TN", "PR", "PD"))
Figure_4B <- 
  Maynard_Epi_pseudobulk@meta.data %>%
  filter(!is.na(biopsy_timing)) %>%
  #filter(biopsy_timing %in% c("Early", "Late")) %>%
  ggplot(aes(x = analysis2, y = BM, fill = analysis2)) +
  theme_pubr(base_size = 7) +
  geom_boxplot(outlier.shape = NA) +
  geom_jitter(width = 0.2, size = 0.5) +
  ylab("BM") + xlab("TKI response") +
  ggpubr::geom_pwc(method = "wilcox_test", label = "p.adj.signif", p.adjust.method = "fdr", hide.ns = T, label.size = 2, tip.length = 0, vjust = 0.5) +
  expand_limits(y = 1.0) + scale_fill_manual(values = c("#794DFF", "#FFD3C4", "#FF6341")) + NoLegend() + ylab("BM (ssGSEA score)")
Figure_4B
```

```
ggsave(Figure_4B, path = Plots_out, file = "Figure_4B.svg", width = 3.5, height = 4.5, units = "cm")
```

Figure 4c-d, Maynard et al. single-cell ALV and BM module scores

```
Maynard_Epi_seurat <- AddModuleScore(Maynard_Epi_seurat, features = Dev.sig_list)

Maynard_Epi_seurat$analysis3 <- factor(Maynard_Epi_seurat$analysis,
                                       levels = c("grouped_pr", "grouped_pd"),
                                       labels = c("PR", "PD"))


thresh_ALV = median(Maynard_Epi_seurat$Cluster1)+mad(Maynard_Epi_seurat$Cluster1)
thresh_BM = median(Maynard_Epi_seurat$Cluster2)+mad(Maynard_Epi_seurat$Cluster2)

Maynard_Epi_seurat$Alv_BM.class <- 
  ifelse(Maynard_Epi_seurat$Cluster1 > thresh_ALV & Maynard_Epi_seurat$Cluster2 < thresh_BM, "ALV+BM-", 
         ifelse(Maynard_Epi_seurat$Cluster1 < thresh_ALV & Maynard_Epi_seurat$Cluster2 < thresh_BM, "ALV-BM-", 
                ifelse(Maynard_Epi_seurat$Cluster1 < thresh_ALV & Maynard_Epi_seurat$Cluster2 > thresh_BM, "ALV-BM+", "ALV+BM+")))

Figure_4C <-
  Maynard_Epi_seurat@meta.data %>%
  ggplot(aes(x = Cluster1, y = Cluster2, colour = Alv_BM.class)) +
  theme_pubr(base_size = 7) +
  scattermore::geom_scattermore(pointsize = 2, dpi = 521) +
  geom_hline(yintercept = thresh_BM, linetype = "dotted") + geom_vline(xintercept = thresh_ALV, linetype = "dotted") +
  theme(legend.position = "none") +
  xlab("ALV (module score)") + ylab("BM (module score)") + scale_color_manual(values = c("dodgerblue3", "darkorange3", "forestgreen", "maroon2"))

ggsave(Figure_4C, path = Plots_out, file = "Figure_4C.svg", width = 4.5, height = 4.5, units = "cm")


Figure_4D <- 
  Maynard_Epi_seurat@meta.data %>%
  filter(!is.na(analysis3)) %>%
  ggplot(aes(x = analysis3, fill = Alv_BM.class)) +
  theme_pubr(base_size = 7) +
  geom_bar(position = "fill") +
  xlab("TKI response") + 
  ylab("Fraction of All Epithelial Cells") +
  theme(legend.position = "right", legend.title = element_blank(), legend.key.size = unit(5,"pt")) +
  scale_fill_manual(values = c("dodgerblue3", "darkorange3", "forestgreen", "maroon2"))
ggsave(Figure_4D, path = Plots_out, file = "Figure_4D.svg", width = 4.5, height = 4.5, units = "cm")

Figure_4C + Figure_4D
```

Figure 4F

```
# TKI survival
# adding ssGSEA scores
ssGSEA_TKI <- GSVA::ssgseaParam(expr=mRNA_filtered[!duplicated(rownames(mRNA_filtered)),],
                         Dev.sig_list)

ssGSEA_TKI <- gsva(ssGSEA_TKI, verbose = T)

rownames(clinicalData) <- clinicalData$Sample.ID
clinicalData <- merge(clinicalData, t(ssGSEA_TKI), by = 0)
rownames(clinicalData) <- clinicalData$Row.names
names(clinicalData)
clinicalData <- clinicalData[,-1]

clinicalData$DFS <- 1
clinicalData$DFS_YEARS <- clinicalData$Disease.free.survival.months/12

cutpoint <- surv_cutpoint(clinicalData, time = "DFS_YEARS", event = "DFS",
                          "BM", minprop = 0.1, progressbar = TRUE)
surv_cat <- surv_categorize(cutpoint)

surv_cat$BM <- factor(surv_cat$BM, levels = c("low", "high"))

Figure_4F_surv <- ggsurvplot(fit = survfit(Surv(DFS_YEARS, DFS) ~ BM, data = surv_cat),
           data = surv_cat, risk.table = T, pval = T, conf.int = T,
           pval.coord = c(0,0), pval.size = 2,
           conf.int.alpha = 0.05,
           theme = "theme_pubr",
           palette = "aaas",
           ylab = "DFS (Probability)", legend.labs = c("BM-Low", "BM-High"),
           censor.size = 1, censor.shape = 124, fontsize = 2) +
  theme_survminer(base_size = 7)

Figure_4F <- 
  ggarrange(Figure_4F_surv$plot + theme_pubr(base_size = 7) + theme(legend.position = c(1,1), legend.justification = c(1,1), legend.key.size = unit(5,"pt"), axis.title.x = element_blank(), axis.text.x = element_blank(), legend.background = element_rect(fill='transparent'), legend.title = element_blank()),
            Figure_4F_surv$table + theme_pubr(base_size = 7) + theme(plot.title = element_blank()) + xlab("Time (Years)"),
            heights = c(2,1), nrow = 2, align = "v")
Figure_4F
```

```
ggsave(Figure_4F, path = Plots_out, file = "Figure_4F.svg", width = 6.5, height = 4.5, units = "cm")
```

Figure 4H

```
#Alv and BM signatures####
ssGSEA_GSE135222_ALV.BM <- GSVA::ssgseaParam(expr=as.matrix(log2(GSE135222_TPM_filtered+1)),
                                      Dev.sig_list)

ssGSEA_GSE135222_ALV.BM <- gsva(ssGSEA_GSE135222_ALV.BM, verbose = T)
rownames(GSE135222_traits) <- gsub(" ", "", GSE135222_traits$PatientID, fixed = T)
GSE135222_traits <- merge(GSE135222_traits[,-1], t(ssGSEA_GSE135222_ALV.BM), by = 0)

#ALL - KM analysis
Opt_cuts <- surv_cutpoint(GSE135222_traits,
                          time = "PFS_YEARS", event = "PFS",
                          variables = c("BM", "ALV"),
                          minprop = 0.1, progressbar = TRUE)

GSE135222_Opt_Cat <- surv_categorize(Opt_cuts, variables = NULL, labels = c("Low", "High"))
GSE135222_Opt_Cat$BM <- factor(GSE135222_Opt_Cat$BM,
                                          levels = c("Low", "High"))

Figure_4H_surv <- ggsurvplot(survfit(Surv(PFS_YEARS, PFS) ~ BM, data = GSE135222_Opt_Cat),
                       data = GSE135222_Opt_Cat, risk.table = T, pval = T, conf.int = T,
                       pval.coord = c(0,0), pval.size = 2,
                       conf.int.alpha = 0.05,
                       theme = "theme_pubr",
                       palette = "aaas",
                       ylab = "PFS (Probability)", legend.labs = c("BM-Low", "BM-High"),
                       censor.size = 1, censor.shape = 124, fontsize = 2) +
  theme_survminer(base_size = 7)

Figure_4H <- 
  ggarrange(Figure_4H_surv$plot + theme_pubr(base_size = 7) + theme(legend.position = c(1,1), legend.justification = c(1,1), legend.key.size = unit(5,"pt"), axis.title.x = element_blank(), axis.text.x = element_blank(), legend.background = element_rect(fill='transparent'), legend.title = element_blank()),
            Figure_4H_surv$table + theme_pubr(base_size = 7) + theme(plot.title = element_blank()) + xlab("Time (Years)"),
            heights = c(2,1), nrow = 2, align = "v")
Figure_4H
```

```
ggsave(Figure_4H, path = Plots_out, file = "Figure_4H.svg", width = 6.5, height = 4.5, units = "cm")
```

Figure 4J

```
#GSE207422####
GSE207422_Tumour.samples <- GSE207422_metadata %>% filter(!Resource == "Pre_biopsy") %>% select(Sample)

ssGSEA_GSE207422_ALV.BM <- GSVA::ssgseaParam(expr=as.matrix(GSE207422_logTPM[ , names(GSE207422_logTPM) %in% GSE207422_Tumour.samples$Sample]), Dev.sig_list)

ssGSEA_GSE207422_ALV.BM <- gsva(ssGSEA_GSE207422_ALV.BM)


rownames(GSE207422_metadata) <- GSE207422_metadata$Sample
GSE207422_metadata <- merge(GSE207422_metadata, t(ssGSEA_GSE207422_ALV.BM), by = 0)
GSE207422_metadata <- GSE207422_metadata[, !duplicated(names(GSE207422_metadata))]
GSE207422_metadata$MPR.Binary <- factor(GSE207422_metadata$MPR.Binary, levels = c("TRUE", "FALSE"))
levels(GSE207422_metadata$MPR.Binary) <- c("Responder", "Non-responder")

Figure_4J <- 
  GSE207422_metadata %>%
  filter(!Resource == "Pre_biopsy") %>% #Exclude PBMC samples
  ggplot(aes(x = MPR.Binary, y = BM, fill = MPR.Binary)) +
  geom_boxplot(outlier.shape = NA) +
  geom_jitter(width = 0.2, size = 0.5) +
  ggpubr::geom_pwc(method = "wilcox_test", label = "p.signif", p.adjust.method = "fdr", hide.ns = F, label.size = 2, tip.length = 0, bracket.nudge.y = -0.04) +
  theme_pubr(base_size = 7) +
  rotate_x_text(angle = 45) +
  theme(legend.position = "none") +
  xlab("ICB response") +
  ylab("BM (ssGSEA score)") +
  scale_fill_manual("LUAD", values = c("#794DFF", "#FF6341"))
Figure_4J
```

```
ggsave(Figure_4J, path = Plots_out, file = "Figure_4J_corrected.svg", width = 3, height = 4.5, units = "cm")
```

Figure 4L-M

```
GSE207422_seurat <- PercentageFeatureSet(GSE207422_seurat, pattern = "^KRT", col.name = "PanCK.pct")
GSE207422_seurat_Epi <- GSE207422_seurat[, GSE207422_seurat$seurat_clusters %in% c(4,7,9,10,13,14) &
                                           GSE207422_seurat$Pathology == "Adeno"]

GSE207422_seurat_Epi <- AddModuleScore(GSE207422_seurat_Epi, features = Dev.sig_list, assay = "RNA")

thresh_ALV = median(GSE207422_seurat_Epi$Cluster1)+mad(GSE207422_seurat_Epi$Cluster1)
thresh_BM = median(GSE207422_seurat_Epi$Cluster2)+mad(GSE207422_seurat_Epi$Cluster2)

GSE207422_seurat_Epi$Alv_BM.class <- 
  ifelse(GSE207422_seurat_Epi$Cluster1 > thresh_ALV & GSE207422_seurat_Epi$Cluster2 < thresh_BM, "ALV+BM-", 
         ifelse(GSE207422_seurat_Epi$Cluster1 < thresh_ALV & GSE207422_seurat_Epi$Cluster2 < thresh_BM, "ALV-BM-", 
                ifelse(GSE207422_seurat_Epi$Cluster1 < thresh_ALV & GSE207422_seurat_Epi$Cluster2 > thresh_BM, "ALV-BM+", "ALV+BM+")))

Figure_4L <- 
  GSE207422_seurat_Epi@meta.data %>%
  ggplot(aes(x = Cluster1, y = Cluster2, colour = Alv_BM.class)) +
  theme_pubr(base_size = 7) +
  scattermore::geom_scattermore(pointsize = 2, dpi = 521) +
  theme(legend.position = "none") +
  geom_hline(yintercept = thresh_BM, linetype = "dotted") + geom_vline(xintercept = thresh_ALV, linetype = "dotted") +
  xlab("ALV (module score)") + ylab("BM (module score)") + scale_color_manual(values = c("dodgerblue3", "darkorange3", "forestgreen", "maroon2"))

ggsave(Figure_4L, path = Plots_out, file = "Figure_4L.svg", width = 4, height = 4.5, units = "cm")


GSE207422_seurat_Epi$MPR.Binary <- factor(GSE207422_seurat_Epi$Pathologic.Response,
                                          levels = c("pCR", "MPR", "NMPR"),
                                          labels = c("Responder", "Responder", "Non-responder"))
Figure_4M <- 
  GSE207422_seurat_Epi@meta.data %>%
  filter(!is.na(MPR.Binary)) %>%
  ggplot(aes(x = factor(MPR.Binary, labels = c("R", "NR")), fill = Alv_BM.class)) +
  theme_pubr(base_size = 7) +
  geom_bar(position = "fill") +
  xlab("ICB Response") + 
  ylab("Fraction of All Epithelial Cells") +
  theme(legend.position = "right",
        legend.title = element_blank(), legend.key.size = unit(5,"pt")) +
  scale_fill_manual(values = c("dodgerblue3", "darkorange3", "forestgreen", "maroon2"))

ggsave(Figure_4M, path = Plots_out, file = "Figure_4M.svg", width = 4, height = 4.5, units = "cm")

Figure_4L + Figure_4M
```

SUPPLEMENTARY Figure S4A-B

```
Maynard_Epi_pseudobulk$FirstlineTKI <- 
  Maynard_Epi_pseudobulk$treatment_history_detail %in% c("on firstline TKI", "on firsline TKI")
Maynard_Epi_pseudobulk$analysis2 <- factor(Maynard_Epi_pseudobulk$analysis,
                                           levels = c("naive", "grouped_pr", "grouped_pd"),
                                           labels = c("TN", "PR", "PD"))
Maynard_Epi_pseudobulk$exc.Normal <- !Maynard_Epi_pseudobulk$pathlogy_review %in% c("positive", "no eval")

driver_order <- Maynard_Epi_pseudobulk@meta.data %>%
  dplyr::select(driver_gene) %>% table() %>% as.data.frame() %>% arrange(Freq)
Figure_S4A <- 
  Maynard_Epi_pseudobulk@meta.data %>%
  filter(!is.na(biopsy_timing), histolgy == "Adenocarcinoma", exc.Normal == F) %>%
  ggplot(aes(x = factor(driver_gene, levels = rev(driver_order$driver_gene)), fill = analysis2)) +
  geom_bar() +
  theme_pubr(base_size = 12) +
  scale_fill_manual(values = c("#794DFF", "#FFD3C4", "#FF6341")) +
  theme(legend.title = element_blank(),
        legend.position = "right") +
  rotate_x_text(angle = 45) +
  xlab("Driver gene")


EGFR.TKI_order <- Maynard_Epi_pseudobulk@meta.data %>%
  filter(!is.na(biopsy_timing), histolgy == "Adenocarcinoma", exc.Normal == F, driver_gene == "EGFR", analysis2 %in% c("PR", "PD")) %>%
  dplyr::select(treatment) %>% table() %>% as.data.frame() %>% arrange(Freq)
Figure_S4Bii <- 
  Maynard_Epi_pseudobulk@meta.data %>%
  filter(!is.na(biopsy_timing), histolgy == "Adenocarcinoma", exc.Normal == F, driver_gene == "EGFR", analysis2 %in% c("PR", "PD") ) %>%
  ggplot(aes(x = factor(treatment, levels = rev(EGFR.TKI_order$treatment)), fill = primary_or_metastaic)) +
  geom_bar() +
  theme_pubr(base_size = 12) +
  theme(legend.position = "right") +
  xlab("EGFRi") +
  scale_fill_manual(name = "Sample Site", values = c("#794DFF", "#FF6341")) +
  rotate_x_text(angle = 45)


ALK.TKI_order <- Maynard_Epi_pseudobulk@meta.data %>%
  filter(!is.na(biopsy_timing), histolgy == "Adenocarcinoma", exc.Normal == F, driver_gene == "ALK", analysis2 %in% c("PR", "PD")) %>%
  dplyr::select(treatment) %>% table() %>% as.data.frame() %>% arrange(Freq)
Figure_S4Bi <- 
  Maynard_Epi_pseudobulk@meta.data %>%
  filter(!is.na(biopsy_timing), histolgy == "Adenocarcinoma", exc.Normal == F, driver_gene == "ALK", analysis2 %in% c("PR", "PD")) %>%
  ggplot(aes(x = factor(treatment, levels = rev(ALK.TKI_order$treatment)), fill = primary_or_metastaic)) +
  geom_bar() +
  theme_pubr(base_size = 12) +
  theme(legend.position = "none") +
  rotate_x_text(angle = 45) +
  scale_fill_manual(name = "Sample Site", values = c("#794DFF", "#FF6341")) +
  xlab("ALKi")


driver_plots <- ggarrange(Figure_S4A,
                          NULL,
                          Figure_S4Bi,
                          Figure_S4Bii,
                          align = "h",
                          ncol = 4, widths = c(1.5,0.25,1,1.5) )
driver_plots
```

Figure S4C

```
Figure_S4C <- 
  Maynard_Epi_pseudobulk@meta.data %>%
  filter(!is.na(biopsy_timing), histolgy == "Adenocarcinoma", exc.Normal == F, stage.at.dx == "IV" ) %>%
  ggplot(aes(x = analysis2, y = BM, fill = analysis2)) +
  theme_pubr(base_size = 12) +
  geom_boxplot(outlier.shape = NA) +
  geom_jitter(width = 0.2, size = 1, show.legend = T) +
  theme(axis.title.x = element_text(size = 12),
        axis.title.y = element_text(size = 12),
        axis.text.x = element_text(size=12),
        axis.text.y = element_text(size=12)) +
  #facet_wrap(~stage.at.dx) +
  ylab("BM (ssGSEA score)") + xlab("TKI response (stage IV LUAD)") +
  ggpubr::geom_pwc(method = "wilcoxon", label = "p.adj.signif", hide.ns = T,
                   p.adjust.method = "fdr", label.size = 4, tip.length = 0, step.increase = 0.1, 
                   y.position = 0.9) +
  expand_limits(y = 1.0) + scale_fill_manual(values = c("#794DFF", "#FFD3C4", "#FF6341")) + NoLegend()

Figure_S4C
```

Figure S4D

```
cutpoint <- surv_cutpoint(clinicalData, time = "DFS_YEARS", event = "DFS",
                          c("BM"), minprop = 0.1, progressbar = TRUE)
surv_cat <- surv_categorize(cutpoint)

surv_cat$BM <- factor(surv_cat$BM, levels = c("low", "high"))


TKI_all.data <- merge(surv_cat, clinicalData, by = 0)
names(TKI_all.data)[names(TKI_all.data) == 'BM.y'] <- 'BM'


Figure_S4D <- TKI_all.data %>%
  ggplot(aes(y = BM, x = T790M, fill = T790M)) +
  geom_boxplot(outlier.shape = NA) +
  geom_jitter(width = 0.1) +
  theme_pubr(base_size = 12) +
  ylab("BM (ssGSEA score)") +
  stat_pwc(label = "p.signif") +
  theme(axis.title.x = element_blank(), legend.position = "none") +
  scale_fill_manual(values = c("#794DFF", "#FF6341")) +
  rotate_x_text(angle = 45)

Figure_S4D
```

Figure S4E

```
Figure_S4E_surv <- ggsurvplot(fit = survfit(Surv(DFS_YEARS.x, DFS.x) ~ T790M, data = TKI_all.data),
                           data = TKI_all.data, pval = TRUE,
                           pval.coord = c(0,0), pval.size = 2,
                           conf.int = T, conf.int.alpha =0.05,
                           theme = "theme_pubr",
                           legend.labs = c("T790M-", "T790M+"),
                           ylab = "DFS (probability)",
                           palette = "aaas", censor.size = 1, censor.shape = 124,
                           risk.table = T, fontsize = 2) +
  theme_survminer(base_size = 7,
                  legend = c(0.7,0.9))

Figure_S4E <- 
  ggarrange(Figure_S4E_surv$plot + theme_pubr(base_size = 7) + theme(legend.position = c(1,1), legend.justification = c(1,1), legend.key.size = unit(5,"pt"), axis.title.x = element_blank(), axis.text.x = element_blank(), legend.background = element_rect(fill='transparent'), legend.title = element_blank()),
            Figure_S4E_surv$table + theme_pubr(base_size = 7) + theme(plot.title = element_blank()) + xlab("Time (Years)"),
            heights = c(2,1), nrow = 2, align = "v")
Figure_S4E
```

```
ggsave(Figure_S4E, path = Plots_out, file = "Figure_S4E.svg", width = 7, height = 4.5, units = "cm")
```

Figure S4F

```
Figure_S4F_surv <- ggsurvplot(fit = survfit(Surv(DFS_YEARS.x, DFS.x) ~ BM.x, 
                                         data = TKI_all.data %>% filter(T790M == "T790M+")),
                           data = TKI_all.data %>% filter(T790M == "T790M+"),
                           pval = TRUE, pval.coord = c(0,0), pval.size = 2,
                           conf.int = T, conf.int.alpha =0.05,
                           theme = "theme_pubr",
                           legend.labs = c("BM-Low", "BM-High"),
                           ylab = "DFS (probability)",
                           palette = "aaas", censor.size = 1, censor.shape = 124,
                           risk.table = T, fontsize = 2) +
  theme_survminer(base_size = 7, legend = c(0.7,0.9))

Figure_S4F <- 
  ggarrange(Figure_S4F_surv$plot + theme_pubr(base_size = 7) + theme(legend.position = c(1,1), legend.justification = c(1,1), legend.key.size = unit(5,"pt"), axis.title.x = element_blank(), axis.text.x = element_blank(), legend.background = element_rect(fill='transparent'), legend.title = element_blank()) + ggtitle("T790M+ cases"),
            Figure_S4F_surv$table + theme_pubr(base_size = 7) + theme(plot.title = element_blank()) + xlab("Time (Years)"),
            heights = c(2,1), nrow = 2, align = "v")
Figure_S4F
```

```
ggsave(Figure_S4F, path = Plots_out, file = "Figure_S4F.svg", width = 7, height = 4.5, units = "cm")
```

Figure S4G

```
TKI_all.data2 <- TKI_all.data[, c(1:4,24)]
names(TKI_all.data2)[names(TKI_all.data2) == 'BM.x'] <- 'BM'
Figure_S4G <- forest_model(coxph(Surv(DFS_YEARS.x, DFS.x) ~ T790M + BM, data = TKI_all.data2))
Figure_S4G
```

FIGURE S4H

```
# PDL1
ssGSEA_Dev_TCGA <- ssgseaParam(NSCLC_vsd_all, Dev.sig_list)
gsva.es <- gsva(ssGSEA_Dev_TCGA, verbose = T)

NSCLC_traits_all <- merge(NSCLC_traits_all, t(gsva.es), by = 0)
rownames(NSCLC_traits_all) <- NSCLC_traits_all$Row.names
NSCLC_traits_all <- NSCLC_traits_all[,-1]

LUAD_traits <- NSCLC_traits_all[NSCLC_traits_all$Subtype == "LUAD", ]

#based on LUAD TCGA surv cutpoint for 5year OS
LUAD_traits$Dev_prog <- NA
LUAD_traits$Dev_prog[LUAD_traits$BM > 0.1881233] <- "BM_high"
LUAD_traits$Dev_prog[LUAD_traits$BM < 0.1881233] <- "BM_low"


rownames(PDL1) <- PDL1$SAMPLE_ID
colnames(PDL1)[colnames(PDL1) == "CD274"] = "PDL1"
LUAD_traits$PDL1 <- PDL1$PDL1[match(LUAD_traits$Patient_Type, PDL1$SAMPLE_ID)]

LUAD_traits$Dev_prog <- factor(LUAD_traits$Dev_prog, levels = c("BM_low", "BM_high"), labels = c("BM-Low", "BM-High"))
my.comparisons <- list(c("BM-Low", "BM-High"))


# when LUAD grouped into ALV and BM groups based on BM score
Figure_S4H <- LUAD_traits %>% drop_na(PDL1) %>%
  ggplot(aes(x = Dev_prog, y = PDL1, fill = Dev_prog)) +
  theme_pubr(base_size = 12) +
  geom_boxplot() +
  #geom_jitter(color="black", alpha=0.9) +
  stat_pwc(label = "p.signif") +
  scale_fill_manual("LUAD", values = c("#794DFF", "#FF6341")) + theme(axis.title.x = element_blank(),
                                                                      axis.title.y = element_text(size = 12),
                                                                      axis.text.x = element_text(size=12),
                                                                      axis.text.y = element_text(size=12),
                                                                      legend.text = element_text(size=12),
                                                                      legend.title = element_text(size=14)) +
  ylab("PDL1 (RPPA)")

Figure_S4H
```

## Session Info

```
print(sessionInfo(), RNG = TRUE, locale = FALSE)
```

```
## R version 4.4.0 (2024-04-24 ucrt)
## Platform: x86_64-w64-mingw32/x64
## Running under: Windows 10 x64 (build 19045)
## 
## Matrix products: default
## 
## 
## Random number generation:
##  RNG:     Mersenne-Twister 
##  Normal:  Inversion 
##  Sample:  Rejection 
##  
## attached base packages:
## [1] stats     graphics  grDevices utils     datasets  methods   base     
## 
## other attached packages:
##  [1] forestmodel_0.6.2  tidyr_1.3.1        ggsci_3.2.0        survminer_0.5.0   
##  [5] survival_3.8-3     stringr_1.5.1      readr_2.1.5        dplyr_1.1.4       
##  [9] GSVA_2.0.5         Seurat_5.2.1       SeuratObject_5.0.2 sp_2.2-0          
## [13] ggpubr_0.6.0       ggplot2_3.5.1     
## 
## loaded via a namespace (and not attached):
##   [1] ggtext_0.1.2                matrixStats_1.5.0          
##   [3] spatstat.sparse_3.1-0       httr_1.4.7                 
##   [5] RColorBrewer_1.1-3          tools_4.4.0                
##   [7] sctransform_0.4.1           backports_1.5.0            
##   [9] R6_2.5.1                    HDF5Array_1.34.0           
##  [11] lazyeval_0.2.2              uwot_0.2.2                 
##  [13] rhdf5filters_1.18.0         withr_3.0.2                
##  [15] gridExtra_2.3               progressr_0.15.1           
##  [17] cli_3.6.2                   Biobase_2.66.0             
##  [19] textshaping_1.0.0           exactRankTests_0.8-35      
##  [21] spatstat.explore_3.3-4      fastDummies_1.7.5          
##  [23] labeling_0.4.3              sass_0.4.9                 
##  [25] mvtnorm_1.3-3               survMisc_0.5.6             
##  [27] spatstat.data_3.1-4         ggridges_0.5.6             
##  [29] pbapply_1.7-2               systemfonts_1.3.1          
##  [31] svglite_2.2.2               parallelly_1.42.0          
##  [33] rstudioapi_0.17.1           RSQLite_2.3.9              
##  [35] generics_0.1.3              ica_1.0-3                  
##  [37] spatstat.random_3.3-2       car_3.1-3                  
##  [39] Matrix_1.7-2                S4Vectors_0.44.0           
##  [41] abind_1.4-8                 lifecycle_1.0.4            
##  [43] yaml_2.3.10                 carData_3.0-5              
##  [45] SummarizedExperiment_1.36.0 rhdf5_2.50.2               
##  [47] SparseArray_1.6.1           Rtsne_0.17                 
##  [49] grid_4.4.0                  blob_1.2.4                 
##  [51] promises_1.3.2              crayon_1.5.3               
##  [53] miniUI_0.1.1.1              lattice_0.22-6             
##  [55] beachmat_2.22.0             cowplot_1.1.3              
##  [57] annotate_1.84.0             KEGGREST_1.46.0            
##  [59] magick_2.8.5                pillar_1.10.1              
##  [61] knitr_1.49                  GenomicRanges_1.58.0       
##  [63] rjson_0.2.23                future.apply_1.11.3        
##  [65] codetools_0.2-20            glue_1.7.0                 
##  [67] spatstat.univar_3.1-1       data.table_1.15.4          
##  [69] vctrs_0.6.5                 png_0.1-8                  
##  [71] spam_2.11-1                 gtable_0.3.6               
##  [73] cachem_1.1.0                xfun_0.50                  
##  [75] S4Arrays_1.6.0              mime_0.12                  
##  [77] SingleCellExperiment_1.28.1 KMsurv_0.1-5               
##  [79] fitdistrplus_1.2-2          ROCR_1.0-11                
##  [81] nlme_3.1-167                bit64_4.6.0-1              
##  [83] RcppAnnoy_0.0.22            GenomeInfoDb_1.42.3        
##  [85] bslib_0.9.0                 maxstat_0.7-25             
##  [87] irlba_2.3.5.1               KernSmooth_2.23-26         
##  [89] colorspace_2.1-1            BiocGenerics_0.52.0        
##  [91] DBI_1.2.3                   tidyselect_1.2.1           
##  [93] bit_4.5.0.1                 compiler_4.4.0             
##  [95] graph_1.84.1                xml2_1.3.6                 
##  [97] DelayedArray_0.32.0         plotly_4.10.4              
##  [99] scales_1.3.0                lmtest_0.9-40              
## [101] SpatialExperiment_1.16.0    digest_0.6.35              
## [103] goftest_1.2-3               spatstat.utils_3.1-2       
## [105] rmarkdown_2.29              XVector_0.46.0             
## [107] htmltools_0.5.8.1           pkgconfig_2.0.3            
## [109] sparseMatrixStats_1.18.0    MatrixGenerics_1.18.1      
## [111] fastmap_1.2.0               rlang_1.1.4                
## [113] htmlwidgets_1.6.4           UCSC.utils_1.2.0           
## [115] shiny_1.10.0                farver_2.1.2               
## [117] jquerylib_0.1.4             zoo_1.8-12                 
## [119] jsonlite_1.8.9              BiocParallel_1.40.0        
## [121] BiocSingular_1.22.0         magrittr_2.0.3             
## [123] Formula_1.2-5               GenomeInfoDbData_1.2.13    
## [125] dotCall64_1.2               patchwork_1.3.0            
## [127] Rhdf5lib_1.28.0             munsell_0.5.1              
## [129] Rcpp_1.0.14                 reticulate_1.40.0          
## [131] stringi_1.8.4               zlibbioc_1.52.0            
## [133] MASS_7.3-64                 plyr_1.8.9                 
## [135] parallel_4.4.0              listenv_0.9.1              
## [137] ggrepel_0.9.6               deldir_2.0-4               
## [139] Biostrings_2.74.1           splines_4.4.0              
## [141] gridtext_0.1.5              tensor_1.5                 
## [143] hms_1.1.3                   igraph_2.1.4               
## [145] spatstat.geom_3.3-5         ggsignif_0.6.4             
## [147] RcppHNSW_0.6.0              reshape2_1.4.4             
## [149] stats4_4.4.0                ScaledMatrix_1.14.0        
## [151] XML_3.99-0.18               evaluate_1.0.3             
## [153] tzdb_0.4.0                  httpuv_1.6.15              
## [155] RANN_2.6.2                  purrr_1.0.4                
## [157] polyclip_1.10-7             future_1.34.0              
## [159] km.ci_0.5-6                 scattermore_1.2            
## [161] rsvd_1.0.5                  broom_1.0.7                
## [163] xtable_1.8-4                RSpectra_0.16-2            
## [165] rstatix_0.7.2               later_1.4.1                
## [167] viridisLite_0.4.2           ragg_1.5.0                 
## [169] tibble_3.2.1                memoise_2.0.1              
## [171] AnnotationDbi_1.68.0        IRanges_2.40.1             
## [173] cluster_2.1.8               globals_0.16.3             
## [175] GSEABase_1.68.0
```
