# Supplementary material for: Developmental programmes drive cellular plasticity, disease progression and therapy resistance in lung adenocarcinoma
Source: Mol Oncol. 2026 May 27:10.1002/1878-0261.70263. Online ahead of print. doi: 10.1002/1878-0261.70263 (PMC13398952; doi:10.1002/1878-0261.70263)
Supplement: Supplementary file 1 — File 1. R Markdown HTML reports. [file MOL2-9999-0-s006.zip › Bienkowska_etal_MolOnc_Fig5.html]

Developmental programmes drive cellular plasticity, disease progression and therapy resistance in lung adenocarcinoma


# Developmental programmes drive cellular plasticity, disease progression and therapy resistance in lung adenocarcinoma

### Figure 5 - BM activation in LUAD is associated with a basal-like phenotype

#### Kamila J Bienkowska, Stephany Gallardo Y, Nur S Zainal, Leena Arora, Matthew Ellis, Maria-Antoinette Lopez, Judith Austine, Sai Pittla, Serena J Chee, Aiman Alzetani, Emily C Shaw, Christian H Ottensmeier, Gareth J Thomas, Christopher J Hanley

#### 2025-11-17

## load libraries

```
library(Seurat)
library(ggplot2)
library(ggpubr)
library(dplyr)
library(stringr)
library(WGCNA)
library(impute)
library(preprocessCore)
library(ggcorrplot)
library(ggrepel)
library(matrixStats)
library(ggcorrplot)
library(Nebulosa)
library(ggrepel)
```

## load objects

```
setwd(input_files)
load(file = "Merged filtered seurats, all cell types minus mural and neutrophils, 7.11.23.Rdata")
load(file = "NSCLC_integrated_epi.Rdata")
load("Sample_level_data, with czbiohub.Rdata")
load(file = "scEPI_DevssGSEA, 61 samples,18.02.25.Rdata")
load(file = "BasalCells_metaData.Rdata")
```

## Figure 5a

```
DefaultAssay(Merged.filtered) <- "RNA"

Figure_5a <- 
  data.frame(Merged.filtered@meta.data, Merged.filtered@reductions$umap@cell.embeddings) %>%
  ggplot(aes(x = umap_1, y = umap_2, colour = Meta_Lineage)) +
  scattermore::geom_scattermore() + theme_void(base_size = 7) +
  theme(legend.position = "bottom", legend.key.size = unit(2,"pt"), legend.title = element_blank(), legend.justification = c(0,0)) + 
  ylab("UMAP_2") + xlab("UMAP_1")


Figure_5a
```

```
ggsave(Figure_5a, path = Plots_out, file = "Figure_5a.svg",
       width = 5, height = 4, units = "cm")
```

## Figure 5B

```
####
Idents(Epi.integrated.filtered) <- Epi.integrated.filtered@meta.data$All_subtype
LUAD_cells <- WhichCells(Epi.integrated.filtered, idents = "LUAD")
LUSC_cells <- WhichCells(Epi.integrated.filtered, idents = "LUSC")
Normal_cells <- WhichCells(Epi.integrated.filtered, idents = "Normal")

p3 <- DimPlot(Epi.integrated.filtered, cells.highlight = c(LUSC_cells), pt.size = 1, sizes.highlight = 3,   cols.highlight = "skyblue1", raster = T) & theme_pubr(base_size = 7) & NoLegend()
p2 <- DimPlot(Epi.integrated.filtered, cells.highlight = c(LUAD_cells), pt.size = 1, sizes.highlight = 3, cols.highlight = "darkorange3", raster = T) & theme_pubr(base_size = 7) & NoLegend()
p1 <- DimPlot(Epi.integrated.filtered, cells.highlight = c(Normal_cells), pt.size = 1, sizes.highlight = 3, cols.highlight = "forestgreen", raster = T) & theme_pubr(base_size = 7) & NoLegend()

Figure_5b <- ggarrange(p1,p2, p3, ncol=3, labels = list("Normal", "LUAD", "LUSC"), label.x = 0.5, font.label = list(size=7, face="plain"))
Figure_5b
```

```
ggsave(Figure_5b, path = Plots_out, file = "Figure_5b.svg",
       width = 12.5, height = 4, units = "cm")
```

## Figure 5C - UMAP

```
DefaultAssay(Epi.integrated.filtered) <- "integrated"
Epi.integrated.filtered <-
  FindClusters(Epi.integrated.filtered,
               resolution = 0.05,
               verbose = FALSE)

Epi.integrated.filtered@meta.data$Epi_subpops <- 
  factor(Epi.integrated.filtered$integrated_snn_res.0.05,
         levels = 0:5,
         labels = c("Inflamed", "Basal", "AT2", "AT1", "Ciliated", "Club"))
Epi.integrated.filtered <- SetIdent(Epi.integrated.filtered, value = "Epi_subpops")


Figure_5c <- DimPlot(Epi.integrated.filtered, label = F, pt.size = 2, raster = T)  & theme_pubr(base_size = 7) + theme(legend.position = "bottom", legend.key.size = unit(2, "pt"), legend.background = element_blank())
Figure_5c
```

```
ggsave(Figure_5c, path = Plots_out, file = "Figure_5c.svg",
       width = 4, height = 4, units = "cm")
```

## Figure 5D and S5C

```
Figure_5d <- Sample_MetaData2[] %>%
  dplyr::filter(Epi_SubPop %in% c("Inflamed", "Basal", "AT2")) %>%
  ggplot(aes(x = Sample.Subtype2, y = Epi.pct )) +
  theme_pubr(base_size = 7) +
  facet_wrap(~factor(Epi_SubPop, levels = c( "AT2", "Basal","Inflamed")), ncol = 3) +
  geom_boxplot(outlier.shape = NA, aes(fill = Sample.Subtype2)) +
  scale_fill_manual(values = c("forestgreen", "darkorange3", "skyblue1"), labels=c('Normal', 'LUAD', 'LUSC')) +
  geom_jitter(width = 0.2, size = 0.1) +
  rotate_x_text(angle = 45) +
  geom_pwc(method = "wilcox_test", p.adjust.method = "fdr", label = "p.adj.signif", tip.length = 0, label.size = 2, hide.ns = T, vjust = 0.5, y.position = 100)  +
  theme(axis.title.x = element_blank(), axis.text.x = element_blank(), legend.position = "bottom", legend.title = element_blank()) +
  ylab("% of all Epi cells") +
  scale_y_continuous(breaks = c(0,25,50,75,100), limits = c(0,130)) 

Figure_5d
```

```
ggsave(Figure_5d, path = Plots_out, filename = "Figure_5d.svg",
      width = 5.5, height = 4, unit = "cm")

Figure_S5d <- Sample_MetaData2[] %>%
  dplyr::filter(!Epi_SubPop %in% c("Inflamed", "Basal", "AT2")) %>%
  ggplot(aes(x = Sample.Subtype2, y = Epi.pct )) +
  theme_pubr(base_size = 7) +
  facet_wrap(~Epi_SubPop, ncol = 3) +
  geom_boxplot(outlier.shape = NA, aes(fill = Sample.Subtype2)) +
  scale_fill_manual(values = c("forestgreen", "darkorange3", "skyblue1"), labels=c('Normal', 'LUAD', 'LUSC')) +
  geom_jitter(width = 0.2, size = 0.1) +
  rotate_x_text(angle = 45) +
  geom_pwc(method = "wilcox_test", p.adjust.method = "fdr", label = "p.adj.signif", tip.length = 0, label.size = 2, hide.ns = T, vjust = 0.5)  +
  theme(axis.title.x = element_blank(), axis.text.x = element_blank(), legend.position = "bottom", legend.title = element_blank()) +
  ylab("% of all Epi cells") 

Figure_S5d
```

```
ggsave(Figure_S5d, path = Plots_out, filename = "Figure_S5d.svg",
      width = 7, height = 4, unit = "cm")
```

## Figure 5E

```
Epi.integrated.filtered$SampleID2 <- paste(Epi.integrated.filtered$Dataset, Epi.integrated.filtered$All_sample_ID, sep = "_")
dt <- as.table(as.matrix(table(Epi.integrated.filtered$SampleID2,
                               Epi.integrated.filtered$Epi_subpops)))

Sample.pct <- reshape2::dcast(as.data.frame(dt), formula = Var1 ~ Var2)
Sample.pct.rownames <- Sample.pct$Var1
Sample.pct <- Sample.pct[,2:ncol(Sample.pct)]/rowSums(Sample.pct[,2:ncol(Sample.pct)])*100
rownames(Sample.pct) <- Sample.pct.rownames
Sample.pct <- data.frame(
  row.names = Sample.pct.rownames,
  Dataset = str_split_fixed(Sample.pct.rownames, "_", 2)[,1],
  SampleID = str_split_fixed(Sample.pct.rownames, "_", 2)[,2],
  Sample.pct
)

temp <-  Sample_MetaData2[!duplicated(Sample_MetaData2$SampleID2), ]
Sample.pct.wide <- merge(Sample.pct, temp, by = "SampleID")

Sample.pct.wide <- merge(Sample.pct.wide, cell.type_df.metadata[,c("ALV", "BM", "SampleID")],
                         by = "SampleID")

Epi_subpops <- levels(Epi.integrated.filtered$Epi_subpops)
Cor_res <- WGCNA::corAndPvalue(Sample.pct.wide[, Epi_subpops], 
                               Sample.pct.wide[, c("ALV", "BM")])


new_order <- c("Ciliated", "AT1", "AT2", "Club", "Inflamed", "Basal")
Cor_res$cor[new_order,]
Figure_5E <- ggcorrplot(t(Cor_res$cor[new_order,]), p.mat = t(Cor_res$p[new_order,]), sig.level = 0.01, method = "circle", pch.cex = 2) +
  theme_pubr(base_size = 7) + 
   scale_size_continuous(range = c(0.5, 5)) +
  theme(axis.title = element_blank(), legend.position = "right", legend.key.width = unit(5, "pt"), legend.key.height = unit(10, "pt"), legend.margin = margin(l=-5)) +
  rotate_x_text(angle = 45)
Figure_5E
```

```
ggsave(Figure_5E, path = Plots_out, filename = "Figure_5E.svg",
      width = 3, height = 4, unit = "cm")
```

## Figure 5F-I

```
cells2plot <- paste(Epi.integrated.filtered$Epi_subpops, Epi.integrated.filtered$All_subtype)
cells2plot <- factor(cells2plot, levels = unique(cells2plot),
                  labels = c("Basal (LUSC)", rep(F, 5),
                             "Basal (LUAD)", "Inflamed (LUAD)", "AT2 (LUAD)",
                              rep(F, 3), F, "Basal (Normal)", rep(F, 3), "AT2 (Normal)"))
levels(cells2plot)
cells2plot <- factor(cells2plot, levels = rev(c("Basal (Normal)", "Basal (LUSC)", "Basal (LUAD)", "Inflamed (LUAD)", "AT2 (LUAD)",  "AT2 (Normal)")))
Epi.integrated.filtered$Plotting_Labels_lin.ident <- cells2plot
Basal_cells$Plotting_Labels_lin.ident <- "Basal (Normal)"

matching.cols <- names(Basal_cells)[names(Basal_cells) %in% names(Epi.integrated.filtered@meta.data)]
Lineage.Fidelity.scores <- rbind(Epi.integrated.filtered@meta.data %>% select(all_of(matching.cols)), Basal_cells %>% select(all_of(matching.cols)))

Figure_5f <- 
  plot_density(Epi.integrated.filtered, c("prediction.score.Epi_AT2"), size = 0.1, raster = T) + 
  ggtitle("AT2 Lineage Fidelity") +
  theme_pubr(base_size = 7) +
  theme(legend.position = c("none")) +
  ylab("UMAP_2") + xlab("UMAP_1")

AT2.score_stats <- 
  Lineage.Fidelity.scores %>% group_by(Plotting_Labels_lin.ident) %>% filter(!is.na(Plotting_Labels_lin.ident)) %>%
  summarise(
    median = median(prediction.score.Epi_AT2, na.rm = TRUE),
    q25 = quantile(prediction.score.Epi_AT2, 0.25, na.rm = TRUE),
    q75 = quantile(prediction.score.Epi_AT2, 0.75, na.rm = TRUE),
    n = n()
)


Figure_5g <- 
 Lineage.Fidelity.scores %>%
  filter(!Plotting_Labels_lin.ident == F) %>%
  ggplot(aes(x = Plotting_Labels_lin.ident, y = prediction.score.Epi_AT2, fill = Plotting_Labels_lin.ident)) +
  theme_pubr(base_size = 7) +
  theme(legend.position = "none", axis.title.x = element_blank(), axis.text.x = element_text(size =  5)) +
  geom_text(aes(y = 1.1, x = Plotting_Labels_lin.ident, label = paste0(round(median,2), "\n(", round(q25,1), "-", round(q75,1),")")), data = AT2.score_stats, size = 1.5) +
  ggrastr::geom_jitter_rast(alpha = 0.1, size = 0.1, width = 0.2) +
  geom_violin(scale = "width") +
  ylab("AT2 Lineage Fidelity\n(Probability)") + rotate_x_text(angle = 45)

Figure_5h <- 
  plot_density(Epi.integrated.filtered, ("prediction.score.Epi_Basal"), size = 0.1, raster = T) +
  ggtitle("Basal Lineage Fidelity") +
  theme_pubr(base_size = 7) +
  theme(legend.position = c("none")) +
  ylab("UMAP_2") + xlab("UMAP_1")

Basal.score_stats <- 
  Lineage.Fidelity.scores %>% group_by(Plotting_Labels_lin.ident) %>% filter(!is.na(Plotting_Labels_lin.ident)) %>%
  summarise(
    median = median(prediction.score.Epi_Basal, na.rm = TRUE),
    q25 = quantile(prediction.score.Epi_Basal, 0.25, na.rm = TRUE),
    q75 = quantile(prediction.score.Epi_Basal, 0.75, na.rm = TRUE),
    n = n()
)

Figure_5i <- 
  Lineage.Fidelity.scores %>%
  filter(!Plotting_Labels_lin.ident == F) %>%
  ggplot(aes(x = Plotting_Labels_lin.ident, y = prediction.score.Epi_Basal, fill = Plotting_Labels_lin.ident)) +
  theme_pubr(base_size = 7) +
  theme(legend.position = "none", axis.title.x = element_blank(), axis.text.x = element_text(size =  5)) +
    geom_text(aes(y = 1.1, x = Plotting_Labels_lin.ident, label = paste0(round(median,2), "\n(", round(q25,1), "-", round(q75,1),")")), data = Basal.score_stats, size = 1.5) +
ggrastr::geom_jitter_rast(alpha = 0.1, size = 0.1, width = 0.2) +
  geom_violin(scale = "width") +
  ylab("Basal Lineage Fidelity\n(Probability)") + rotate_x_text(angle = 45) 

LineageFidelity.plots <- ggarrange(Figure_5f, Figure_5g, Figure_5h, Figure_5i, ncol = 4)
LineageFidelity.plots
```

```
ggsave(LineageFidelity.plots, path = Plots_out, filename = "Figure_5FGHI.svg",
      width = 18, height = 5, unit = "cm")
```

## Figure 5J-K

```
#Add to figure script
Epi.integrated.filtered$LUAD_comparison <- NA
Epi.integrated.filtered$LUAD_comparison[Epi.integrated.filtered$All_subtype == "LUAD" & 
                                          Epi.integrated.filtered$Epi_subpops == "Inflamed"] <- "LUAD_Inflamed"
Epi.integrated.filtered$LUAD_comparison[Epi.integrated.filtered$All_subtype == "LUAD" & 
                                          Epi.integrated.filtered$Epi_subpops == "Basal"] <- "LUAD_Basal"

Epi.integrated.filtered$LUAD_comparison[Epi.integrated.filtered$Dataset == "TLDS"] <- NA


## Figure 5J
UMAP_df <- data.frame(Epi.integrated.filtered@meta.data,
           Epi.integrated.filtered@reductions$umap@cell.embeddings)
Figure_5j <- 
  UMAP_df %>%
  ggplot(aes(x = UMAP_1, y = UMAP_2, colour = LUAD_comparison)) +
  scattermore::geom_scattermore(data = UMAP_df %>% filter(is.na(LUAD_comparison)), pointsize  = 2, show.legend = F, colour = "grey90")+
  scattermore::geom_scattermore(data = UMAP_df %>% filter(!is.na(LUAD_comparison)), pointsize  = 2, show.legend = T)+
  scale_color_manual(values=c("brown", "blue", "grey90"), na.translate = FALSE) +
  theme_pubr(base_size = 7) +
  theme(legend.position = "bottom", legend.key.size = unit(2,"pt"),plot.title = element_blank(),legend.title = element_blank()) 

ggsave(Figure_5j, path = Plots_out, filename = "Figure_5j.svg",
      width = 4, height = 4, unit = "cm")

DefaultAssay(Epi.integrated.filtered) <- "RNA"
Idents(Epi.integrated.filtered) <- Epi.integrated.filtered$LUAD_comparison
LUAD_DEGs <- FindConservedMarkers(Epi.integrated.filtered,
                                  ident.1 = "LUAD_Basal", ident.2 = "LUAD_Inflamed",
                                  grouping.var = "Dataset", min.pct = 0, logfc.threshold = 0.1)
LUAD_DEGs$gene <- rownames(LUAD_DEGs)
FC_cols <- grep("log2FC", names(LUAD_DEGs), fixed = T)
pval_cols <- grep("p_val_adj", names(LUAD_DEGs), fixed = T)
LUAD_DEGs$max_pval.adj <- rowMaxs(as.matrix(LUAD_DEGs[, pval_cols]))
LUAD_DEGs$MedianConsensus_log2FC <- rowMedians(as.matrix(LUAD_DEGs[, FC_cols]))

LUAD_DEGs <- LUAD_DEGs %>%
  mutate(Group = case_when(MedianConsensus_log2FC >= 1 & max_pval.adj <= 0.05 ~ "LUAD_Basal",
                           MedianConsensus_log2FC <= -1 & max_pval.adj <= 0.05 ~ "LUAD_Inflamed",
                           TRUE ~ "ns")) 
# with selected genes labelled
#From HLCA - https://www.nature.com/articles/s41586-020-2922-4
basal_markers <- c("KRT5", "KRT14", "TP63", "DAPL1") 
AT1_markers <- c("AGER", "PDPN", "CLIC5")
AT2_markers <- c("SFTPB", "SFTPC", "SFTPD", "MUC1", "ETV5")

genes_to_plot <- c("SFTPD", "SFTPB", "SFTPA2", "ABCA3", "NAPSA", "SCGB3A2", "HOPX", #ALV
                   "KRT17", "KRT19",  "S100A9", #LUSC/basal 
                   "MKI67", "TOP2A" #proliferation
                   )

LUAD_DEGs$labels <- LUAD_DEGs$gene
LUAD_DEGs$labels[!LUAD_DEGs$gene %in% genes_to_plot] <- NA

LUAD_DEGs$basal_markers <- LUAD_DEGs$gene
LUAD_DEGs$basal_markers[!LUAD_DEGs$gene %in% basal_markers] <- NA

#Calculate detection rates
test <- FindMarkers(Epi.integrated.filtered,
                    ident.1 = "LUAD_Basal", ident.2 = "LUAD_Inflamed",
                    features = LUAD_DEGs$gene)
test$max_min.pct <- rowMaxs(as.matrix(test[, 3:4]))
LUAD_DEGs2 <- merge(LUAD_DEGs, test, by = 0)

Figure_5k <- 
  LUAD_DEGs2 %>%
  ggplot(aes(y = MedianConsensus_log2FC, x = max_min.pct, colour = Group)) +
  ggrastr::rasterise(geom_point(data = LUAD_DEGs2 %>% dplyr::filter(Group == "ns"), size = 0.1, alpha = 0.1, colour = "grey90"), dpi = 512) +
  ggrastr::rasterise(geom_point(data = LUAD_DEGs2 %>% dplyr::filter(!Group == "ns"), aes(size = -log10(max_pval.adj))), dpi = 512) +
  scale_size(range = c(0.1,2))+
  theme_pubr(base_size = 7) +
  geom_label_repel(aes(label = labels), show.legend = F, min.segment.length = 0, nudge_x = 0.01, size = 2, fontface = "italic", label.padding = 0.1, box.padding = 0.1) +
  scale_color_manual(values=c("brown", "blue", ""), labels = c("LUAD_Basal", "LUAD_Inflamed", "")) +
  xlab("Detection rate in highest expressing group") + ylab("Median Consensus log2FC") +
  theme(legend.position = "right", legend.key.size = unit(2, "pt"))

ggsave(Figure_5k, path = Plots_out, filename = "Figure_5k.svg",
      width = 8, height = 4, unit = "cm")

Figure_5j + Figure_5k
```

## Figure 5L

```
Epi.integrated.filtered$Basal_cat <- paste(Epi.integrated.filtered$Epi_subpops,
                                           ifelse(Epi.integrated.filtered$prediction.score.Epi_Basal > 0.5, "Typical", "Atypical"))
table(Epi.integrated.filtered$Basal_cat)
Epi.integrated.filtered$Basal_cat[!Epi.integrated.filtered$Epi_subpops == "Basal"] <- NA
Epi.integrated.filtered$Basal_cat <- paste(Epi.integrated.filtered$All_subtype, Epi.integrated.filtered$Basal_cat)
Epi.integrated.filtered$Basal_cat <- factor(Epi.integrated.filtered$Basal_cat,
                                            levels = c("LUAD Basal Atypical", "LUAD Basal Typical", "LUSC Basal Atypical", "LUSC Basal Typical"),
                                            labels = c("Atypical-Basal\n(LUAD)", "Typical-Basal\n(LUAD)", "Basal (LUSC)", "Basal (LUSC)" ))

VlnPlot_df <- data.frame(Epi.integrated.filtered@meta.data, t(Epi.integrated.filtered@assays$RNA@data[c("KRT17", "KRT6A", "KRT5"), ]))

KRT17_vln <- 
  VlnPlot_df %>% dplyr::filter(!is.na(Basal_cat)) %>%
  ggplot(aes(x = Basal_cat, y = KRT17, fill = Basal_cat)) +
  theme_pubr(base_size = 7) +
  ggrastr::geom_jitter_rast(alpha = 0.1, size = 0.1, width = 0.2) +
  geom_violin(scale = "width", alpha = 1) +
  scale_fill_manual(values = c("blue", "brown", "gold")) +
  theme(axis.title.x = element_blank(), axis.text.x = element_blank(), plot.title = element_text(face = "italic"), legend.key.size = unit(5, "pt"), legend.title = element_blank(), axis.title.y = element_text(face = "italic"))
KRT6A_vln <- 
  VlnPlot_df %>% dplyr::filter(!is.na(Basal_cat)) %>%
  ggplot(aes(x = Basal_cat, y = KRT6A, fill = Basal_cat)) +
  theme_pubr(base_size = 7) +
  ggrastr::geom_jitter_rast(alpha = 0.1, size = 0.1, width = 0.2) +
  geom_violin(scale = "width", alpha = 1) +
  scale_fill_manual(values = c("blue", "brown", "gold")) +
  theme(axis.title.x = element_blank(), axis.text.x = element_blank(), plot.title = element_text(face = "italic"), legend.key.size = unit(5, "pt"), legend.title = element_blank(), axis.title.y = element_text(face = "italic"))
KRT5_vln <- 
  VlnPlot_df %>% dplyr::filter(!is.na(Basal_cat)) %>%
  ggplot(aes(x = Basal_cat, y = KRT5, fill = Basal_cat)) +
  theme_pubr(base_size = 7) +
  ggrastr::geom_jitter_rast(alpha = 0.1, size = 0.1, width = 0.2) +
  geom_violin(scale = "width", alpha = 1) +
  scale_fill_manual(values = c("blue", "brown", "gold")) +
  theme(axis.title.x = element_blank(), axis.text.x = element_blank(), plot.title = element_text(face = "italic"), legend.key.size = unit(5, "pt"), legend.title = element_blank(), axis.title.y = element_text(face = "italic"))

Figure_5L <- ggarrange(KRT17_vln, KRT6A_vln, KRT5_vln, ncol = 3, common.legend = T, legend = "bottom")
Figure_5L
```

```
ggsave(Figure_5L, path = Plots_out, filename = "Figure_5L.svg",
      width = 6, height = 4, unit = "cm")
```

## Figure S5A

```
Figure_S5a <- cell.type_df.metadata %>%
  ggplot(aes(y = ALV, x = BM)) +
  theme_pubr(base_size = 7) +
  geom_point(size = 0.5) +
  stat_smooth(method = "lm", colour = "grey30") +
  stat_cor(aes(label = paste(after_stat(r.label),after_stat(p.label), sep = "~`,`~")),colour = "black", size = 2, label.x = Inf, label.y = Inf, hjust = 1, vjust = 1, method = "spearman") + 
  ylab("BM (ssGSEA score)") + xlab("ALV (ssGSEA score)")

Figure_S5a
```

```
ggsave(Figure_S5a, path = Plots_out, filename = "Figure_S5a.svg",
      width = 6, height = 4.5, unit = "cm")
```

## Figure S5b

```
DefaultAssay(Epi.integrated.filtered) <- "RNA"

genes <- c("KRT5", "DAPL1", "TP63", "SFTPC", "SFTPD", "ABCA3", "AGER", "CAV1", "EMP2", "TPPP3", "SNTN", "CAPS", "SCGB1A1",
           "SCGB3A1", "BPIFB1", "SPINK1", "HPGD", "IRF7")

plots <- list()

for (gene in genes) {
  plots[[gene]] <- plot_density(Epi.integrated.filtered, features = gene, size = 0.1)  + ggtitle(NULL) + 
  theme_void(base_size = 0) + NoLegend()
}

Figure_S5b_basal <- wrap_plots(plots[1:3], ncol = 3) 
Figure_S5b_AT2 <- wrap_plots(plots[4:6], ncol = 3) 
Figure_S5b_AT1 <- wrap_plots(plots[7:9], ncol = 3) 
Figure_S5b_Ciliated <- wrap_plots(plots[10:12], ncol = 3) 
Figure_S5b_Club <- wrap_plots(plots[13:15], ncol = 3) 
Figure_S5b_Inflamed <- wrap_plots(plots[16:18], ncol = 3) 

Figure_S5b <- ggarrange(Figure_S5b_basal, Figure_S5b_AT2, Figure_S5b_AT1, Figure_S5b_Ciliated, Figure_S5b_Club, Figure_S5b_Inflamed, ncol = 1)

#Figure_S5b
ggsave(Figure_S5b, path = Plots_out, filename = "Figure_S5b.png", width = 4.5, height = 10, unit = "cm", dpi = 600, bg = "transparent")
```

Figure S5c

```
DefaultAssay(Epi.integrated.filtered) <- "RNA"
HOPX_density <- plot_density(Epi.integrated.filtered, features = "HOPX", size = 0.1) + 
  theme_pubr(base_size = 7) + NoLegend()

VlnPlot_df2 <- data.frame(Epi.integrated.filtered@meta.data, t(Epi.integrated.filtered@assays$RNA@data[c("HOPX", "AGER"), ]))
HOPX_vln <- 
  VlnPlot_df2 %>% 
  ggplot(aes(x = reorder(Epi_subpops, HOPX), y = HOPX, fill = Epi_subpops)) +
  theme_pubr(base_size = 7) +
  ggrastr::geom_jitter_rast(alpha = 0.1, size = 0.1, width = 0.2) +
  geom_violin(scale = "width", alpha = 1) +
  scale_fill_brewer(palette = "Set2") +
  rotate_x_text(angle = 45) + theme(legend.position = "none", axis.title.x = element_blank())

Figure_S5c <- ggarrange(HOPX_density, HOPX_vln, nrow = 2)

ggsave(Figure_S5c, path = Plots_out, filename = "Figure_S5c.svg", width = 5.5, height = 6, unit = "cm", bg = "transparent")
```

## Figure S5e

```
#correlation without LUSC
Sample.pct.wide_no_LUSC <- subset(Sample.pct.wide, Sample.Subtype2 != "LUSC")
Cor_res <- WGCNA::corAndPvalue(Sample.pct.wide_no_LUSC[, Epi_subpops], 
                               Sample.pct.wide_no_LUSC[, c("ALV", "BM")])
Figure_S5e <- ggcorrplot(t(Cor_res$cor[new_order,]), p.mat = t(Cor_res$p[new_order,]), sig.level = 0.01, method = "circle", pch.cex = 2) +
  theme_pubr(base_size = 7) + 
  scale_size_continuous(range = c(0.5, 5)) +
  theme(axis.title = element_blank(), legend.position = "right", legend.key.width = unit(5, "pt"), legend.key.height = unit(10, "pt"), legend.margin = margin(l=-5)) +
  rotate_x_text(angle = 45)
Figure_S5e
```

```
ggsave(Figure_S5e, path = Plots_out, filename = "Figure_S5e.svg",
      width = 3, height = 4, unit = "cm")
```

## Figure S5f

```
# basal markers in LUAD-Inflamed, LUAD-Basal, LUSC
Epi.integrated.filtered$clusters <- NA
Epi.integrated.filtered$clusters[Epi.integrated.filtered$All_subtype == "LUAD" & 
                                          Epi.integrated.filtered$Epi_subpops == "Inflamed"] <- "LUAD_Inflamed"
Epi.integrated.filtered$clusters[Epi.integrated.filtered$All_subtype == "LUAD" & 
                                          Epi.integrated.filtered$Epi_subpops == "Basal"] <- "LUAD_Basal"
Epi.integrated.filtered$clusters[Epi.integrated.filtered$All_subtype == "LUSC"& 
                                   Epi.integrated.filtered$Epi_subpops == "Basal"] <- "LUSC_Basal"

DefaultAssay(Epi.integrated.filtered) <- "RNA"
Epi.integrated.filtered$clusters <- factor(Epi.integrated.filtered$clusters, levels = c("LUAD_Inflamed", "LUAD_Basal", "LUSC_Basal"))
Idents(Epi.integrated.filtered) <- Epi.integrated.filtered$clusters


for_dotplot <- subset(Epi.integrated.filtered, idents = c("LUAD_Inflamed", "LUAD_Basal", "LUSC_Basal"))

# combine into 1 plot
features <- c("SFTPB", "SFTPD", "NAPSA", "HOPX", "ABCA3", "SCGB3A2", "AQP4", "KRT17", "KRT6A", "KRT14", "KRT5", "TP63", "DAPL1", "STMN1", "S100A9", "S100A2")
dotplot <- DotPlot(for_dotplot, features = features, assay = "RNA", group.by = "clusters", cols = c("blue", "red")) + RotatedAxis() 
Figure_S5f <- dotplot + theme_pubr(base_size = 7) + theme(legend.position = "right", legend.key.height = unit(5, "pt"), legend.key.width = unit(2, "pt"), axis.text.x = element_text(face = "italic")) +
  rotate_x_text(angle = 45)
Figure_S5f
```

```
ggsave(Figure_S5f, path = Plots_out, filename = "Figure_S5f.svg",
      width = 12, height = 4.5, unit = "cm")
```

## Figure S5g

```
VlnPlot_df2 <- data.frame(Epi.integrated.filtered@meta.data, t(Epi.integrated.filtered@assays$RNA@data[c("SFTPB", "NAPSA", "ABCA3"), ]))

SFTPB_vln <- 
  VlnPlot_df2 %>% dplyr::filter(!is.na(Basal_cat)) %>%
  ggplot(aes(x = Basal_cat, y = SFTPB, fill = Basal_cat)) +
  theme_pubr(base_size = 7) +
  ggrastr::geom_jitter_rast(alpha = 0.1, size = 0.1, width = 0.2) +
  geom_violin(scale = "width", alpha = 1) +
  scale_fill_manual(values = c("blue", "brown", "gold")) +
  theme(axis.title.x = element_blank(), axis.text.x = element_blank(), plot.title = element_text(face = "italic"), legend.key.size = unit(5, "pt"), legend.title = element_blank(), axis.title.y = element_text(face = "italic"))
NAPSA_vln <- 
  VlnPlot_df2 %>% dplyr::filter(!is.na(Basal_cat)) %>%
  ggplot(aes(x = Basal_cat, y = NAPSA, fill = Basal_cat)) +
  theme_pubr(base_size = 7) +
  ggrastr::geom_jitter_rast(alpha = 0.1, size = 0.1, width = 0.2) +
  geom_violin(scale = "width", alpha = 1) +
  scale_fill_manual(values = c("blue", "brown", "gold")) +
  theme(axis.title.x = element_blank(), axis.text.x = element_blank(), plot.title = element_text(face = "italic"), legend.key.size = unit(5, "pt"), legend.title = element_blank(), axis.title.y = element_text(face = "italic"))
ABCA3_vln <- 
  VlnPlot_df2 %>% dplyr::filter(!is.na(Basal_cat)) %>%
  ggplot(aes(x = Basal_cat, y = ABCA3, fill = Basal_cat)) +
  theme_pubr(base_size = 7) +
  ggrastr::geom_jitter_rast(alpha = 0.1, size = 0.1, width = 0.2) +
  geom_violin(scale = "width", alpha = 1) +
  scale_fill_manual(values = c("blue", "brown", "gold")) +
  theme(axis.title.x = element_blank(), axis.text.x = element_blank(), plot.title = element_text(face = "italic"), legend.key.size = unit(5, "pt"), legend.title = element_blank(), axis.title.y = element_text(face = "italic"))

Figure_S5g <- ggarrange(SFTPB_vln, NAPSA_vln, ABCA3_vln, ncol = 3, common.legend = T, legend = "bottom")

Figure_S5g
```

```
ggsave(Figure_S5g, path = Plots_out, filename = "Figure_S5g.svg",
      width = 6, height = 4, unit = "cm")
```

## Session Info

```
print(sessionInfo(), RNG = TRUE, locale = FALSE)
```

```
## R version 4.4.0 (2024-04-24 ucrt)
## Platform: x86_64-w64-mingw32/x64
## Running under: Windows 10 x64 (build 19045)
## 
## Matrix products: default
## 
## 
## Random number generation:
##  RNG:     Mersenne-Twister 
##  Normal:  Inversion 
##  Sample:  Rejection 
##  
## attached base packages:
## [1] stats     graphics  grDevices utils     datasets  methods   base     
## 
## other attached packages:
##  [1] Nebulosa_1.16.0       patchwork_1.3.0       matrixStats_1.5.0    
##  [4] ggrepel_0.9.6         ggcorrplot_0.1.4.1    preprocessCore_1.68.0
##  [7] impute_1.80.0         WGCNA_1.73            fastcluster_1.2.6    
## [10] dynamicTreeCut_1.63-1 stringr_1.5.1         dplyr_1.1.4          
## [13] ggpubr_0.6.0          ggplot2_3.5.1         Seurat_5.2.1         
## [16] SeuratObject_5.0.2    sp_2.2-0             
## 
## loaded via a namespace (and not attached):
##   [1] spatstat.sparse_3.1-0       httr_1.4.7                 
##   [3] RColorBrewer_1.1-3          doParallel_1.0.17          
##   [5] numDeriv_2016.8-1.1         tools_4.4.0                
##   [7] sctransform_0.4.1           backports_1.5.0            
##   [9] R6_2.5.1                    mgcv_1.9-1                 
##  [11] lazyeval_0.2.2              uwot_0.2.2                 
##  [13] sn_2.1.1                    withr_3.0.2                
##  [15] gridExtra_2.3               progressr_0.15.1           
##  [17] cli_3.6.2                   Biobase_2.66.0             
##  [19] textshaping_1.0.0           Cairo_1.7-0                
##  [21] spatstat.explore_3.3-4      fastDummies_1.7.5          
##  [23] sandwich_3.1-1              labeling_0.4.3             
##  [25] sass_0.4.9                  mvtnorm_1.3-3              
##  [27] spatstat.data_3.1-4         ggridges_0.5.6             
##  [29] pbapply_1.7-2               systemfonts_1.3.1          
##  [31] foreign_0.8-88              svglite_2.2.2              
##  [33] parallelly_1.42.0           plotrix_3.8-4              
##  [35] limma_3.62.2                rstudioapi_0.17.1          
##  [37] RSQLite_2.3.9               generics_0.1.3             
##  [39] ica_1.0-3                   spatstat.random_3.3-2      
##  [41] car_3.1-3                   GO.db_3.20.0               
##  [43] Matrix_1.7-2                ggbeeswarm_0.7.2           
##  [45] S4Vectors_0.44.0            abind_1.4-8                
##  [47] lifecycle_1.0.4             multcomp_1.4-28            
##  [49] yaml_2.3.10                 carData_3.0-5              
##  [51] mathjaxr_1.6-0              SummarizedExperiment_1.36.0
##  [53] SparseArray_1.6.1           Rtsne_0.17                 
##  [55] grid_4.4.0                  blob_1.2.4                 
##  [57] promises_1.3.2              crayon_1.5.3               
##  [59] miniUI_0.1.1.1              lattice_0.22-6             
##  [61] cowplot_1.1.3               KEGGREST_1.46.0            
##  [63] pillar_1.10.1               knitr_1.49                 
##  [65] GenomicRanges_1.58.0        future.apply_1.11.3        
##  [67] codetools_0.2-20            mutoss_0.1-13              
##  [69] glue_1.7.0                  spatstat.univar_3.1-1      
##  [71] data.table_1.15.4           vctrs_0.6.5                
##  [73] png_0.1-8                   spam_2.11-1                
##  [75] Rdpack_2.6.2                gtable_0.3.6               
##  [77] cachem_1.1.0                ks_1.14.3                  
##  [79] xfun_0.50                   rbibutils_2.3              
##  [81] S4Arrays_1.6.0              mime_0.12                  
##  [83] pracma_2.4.4                survival_3.8-3             
##  [85] SingleCellExperiment_1.28.1 iterators_1.0.14           
##  [87] statmod_1.5.0               TH.data_1.1-3              
##  [89] fitdistrplus_1.2-2          ROCR_1.0-11                
##  [91] nlme_3.1-167                bit64_4.6.0-1              
##  [93] RcppAnnoy_0.0.22            GenomeInfoDb_1.42.3        
##  [95] bslib_0.9.0                 irlba_2.3.5.1              
##  [97] vipor_0.4.7                 KernSmooth_2.23-26         
##  [99] rpart_4.1.24                colorspace_2.1-1           
## [101] BiocGenerics_0.52.0         DBI_1.2.3                  
## [103] Hmisc_5.2-2                 nnet_7.3-20                
## [105] ggrastr_1.0.2               mnormt_2.1.1               
## [107] tidyselect_1.2.1            bit_4.5.0.1                
## [109] compiler_4.4.0              htmlTable_2.4.3            
## [111] TFisher_0.2.0               DelayedArray_0.32.0        
## [113] plotly_4.10.4               checkmate_2.3.2            
## [115] scales_1.3.0                lmtest_0.9-40              
## [117] digest_0.6.35               goftest_1.2-3              
## [119] presto_1.0.0                spatstat.utils_3.1-2       
## [121] rmarkdown_2.29              XVector_0.46.0             
## [123] htmltools_0.5.8.1           pkgconfig_2.0.3            
## [125] base64enc_0.1-3             MatrixGenerics_1.18.1      
## [127] fastmap_1.2.0               rlang_1.1.4                
## [129] htmlwidgets_1.6.4           UCSC.utils_1.2.0           
## [131] shiny_1.10.0                farver_2.1.2               
## [133] jquerylib_0.1.4             zoo_1.8-12                 
## [135] jsonlite_1.8.9              mclust_6.1.1               
## [137] magrittr_2.0.3              Formula_1.2-5              
## [139] GenomeInfoDbData_1.2.13     dotCall64_1.2              
## [141] munsell_0.5.1               Rcpp_1.0.14                
## [143] reticulate_1.40.0           stringi_1.8.4              
## [145] zlibbioc_1.52.0             MASS_7.3-64                
## [147] plyr_1.8.9                  parallel_4.4.0             
## [149] listenv_0.9.1               deldir_2.0-4               
## [151] Biostrings_2.74.1           splines_4.4.0              
## [153] multtest_2.62.0             tensor_1.5                 
## [155] qqconf_1.3.2                igraph_2.1.4               
## [157] spatstat.geom_3.3-5         ggsignif_0.6.4             
## [159] RcppHNSW_0.6.0              reshape2_1.4.4             
## [161] stats4_4.4.0                evaluate_1.0.3             
## [163] metap_1.11                  foreach_1.5.2              
## [165] httpuv_1.6.15               RANN_2.6.2                 
## [167] tidyr_1.3.1                 purrr_1.0.4                
## [169] polyclip_1.10-7             future_1.34.0              
## [171] scattermore_1.2             broom_1.0.7                
## [173] xtable_1.8-4                RSpectra_0.16-2            
## [175] rstatix_0.7.2               later_1.4.1                
## [177] viridisLite_0.4.2           ragg_1.5.0                 
## [179] tibble_3.2.1                memoise_2.0.1              
## [181] beeswarm_0.4.0              AnnotationDbi_1.68.0       
## [183] IRanges_2.40.1              cluster_2.1.8              
## [185] globals_0.16.3
```
