# Supplementary material for: Developmental programmes drive cellular plasticity, disease progression and therapy resistance in lung adenocarcinoma
Source: Mol Oncol. 2026 May 27:10.1002/1878-0261.70263. Online ahead of print. doi: 10.1002/1878-0261.70263 (PMC13398952; doi:10.1002/1878-0261.70263)
Supplement: Supplementary file 1 — File 1. R Markdown HTML reports. [file MOL2-9999-0-s006.zip › Bienkowska_etal_MolOnc_Fig6.html]

Developmental programmes drive cellular plasticity, disease progression and therapy resistance in lung adenocarcinoma


# Developmental programmes drive cellular plasticity, disease progression and therapy resistance in lung adenocarcinoma

### Figure 6 - BM activation is found in high grade LUAD tumours

#### Kamila J Bienkowska, Stephany Gallardo Y, Nur S Zainal, Leena Arora, Matthew Ellis, Maria-Antoinette Lopez, Judith Austine, Sai Pittla, Serena J Chee, Aiman Alzetani, Emily C Shaw, Christian H Ottensmeier, Gareth J Thomas, Christopher J Hanley

#### 2025-11-18

Load packages

```
library(GSVA)
library(tidyr)
library(maditr)
library(randomcoloR)
library(ggplot2)
library(ggpubr)
library(stringr)
library(Seurat)
```

Load files

```
setwd(input_files)
load(file="MergedLUADTraits.Rdata")
load(file="MergedLUSCTraits.Rdata")
load(file="Bulk_ALV.BM_scores.Rdata")
load(file="LungTMA_EPI_MxIHC_Data.Rdata")
```

Figure 6A

```
LUAD_all <- merge(Merged.LUADtraits, merged_ALV.BM, by = "Sample.ID")
LUAD_all$Grade2 <- factor(LUAD_all$Grade, levels = c("G1", "well","G2", "mod", "G3", "poor"), labels = c("G1", "G1", "G2", "G2","G3", "G3"))

# 6A 3 dataset BM scores ####
Figure_6A <- LUAD_all %>% drop_na(Grade2) %>%
  ggplot(aes(x = Grade2, y = BM, fill = Grade2)) +
  theme_pubr(base_size = 7) +
  geom_jitter(width = 0.2, size = 0.5, alpha = 0.5) +
  geom_boxplot(outlier.shape = NA) +
  facet_wrap(~Dataset.factor, scales = "free_y") +
  ggpubr::geom_pwc(method = "wilcox_test", p.adjust.method = "fdr",label = "p.adj.signif", 
                   #bracket.nudge.y = -0.2, step.increase = 0.1,
                   vjust = 0.5, hide.ns = T,
                   label.size = 2, tip.length = 0) +
  scale_fill_manual(values = c("#794DFF", "#FFD3C4", "#FF6341")) +
  theme(legend.position = "bottom", legend.title = element_blank(), axis.title.x = element_blank(), axis.text.x = element_blank(), legend.margin = margin(t=-5)) +
  ylab("BM (ssGSEA score)") +
  ggtitle("LUAD Grade")
Figure_6A
```

```
ggsave(Figure_6A, path = Plots_out, file = "Figure_6A.svg",
       width = 7, height = 4.5, units = "cm")

# S6A 3 dataset ALV scores ####
Figure_S6A <- LUAD_all %>% drop_na(Grade2) %>%
  ggplot(aes(x = Grade2, y = ALV, fill = Grade2)) +
  theme_pubr(base_size = 7) +
  geom_jitter(width = 0.2, size = 0.5, alpha = 0.5) +
  geom_boxplot(outlier.shape = NA) +
  facet_wrap(~Dataset.factor, scales = "free_y") +
  ggpubr::geom_pwc(method = "wilcox_test", p.adjust.method = "holm",label = "p.adj.signif", 
                   #bracket.nudge.y = -0.2, step.increase = 0.1,
                   vjust = 0.5, hide.ns = T,
                   label.size = 2, tip.length = 0) +
  scale_fill_manual(values = c("#794DFF", "#FFD3C4", "#FF6341")) +
  theme(legend.position = "bottom", legend.title = element_blank(), axis.title.x = element_blank(), axis.text.x = element_blank(), legend.margin = margin(t=-5)) +
  ylab("ALV (ssGSEA score)") +
  ggtitle("LUAD Grade")
Figure_S6A
```

```
ggsave(Figure_S6A, path = Plots_out, file = "Figure_S6A.svg",
       width = 7, height = 4.5, units = "cm")
```

FIGURE 6D

```
#Functions####
Create_ds_dataset <- function(x, class.col, prop = 0.01, omit){
  #Creates a downsampled dataset for plotting
  #x = Spatial_data: list object with "Cell_class", "exprs" and "input" elements
  #prop = Proportion of data to include
  #class.col = character specifying name of column in Cell_class df to use
  #omit = vector of class labels to omit from DS dataset
  sequence = 1:nrow(x$Cell_class)
  valid_rows = sequence[!is.na(x$Cell_class[[class.col]]) & !x$Cell_class[[class.col]] %in% omit]
  sub_sample <- sample(valid_rows, size = prop * length(valid_rows))
  x_ds <- x
  for(i in names(x)){
    x_ds[[i]] <- x[[i]][sub_sample, ]
  }
  return(x_ds)
}

col_pal <- c("purple", "orange" )
names(col_pal) <- c("ALV/Inflamed", "BM")

#EPI cells####
P2_EPI_NuclearData$Subtype <- factor(
  P2_EPI_NuclearData$Structure,
  levels = unique(P2_EPI_NuclearData$Structure)[c(2,4,5,3)],
  labels = c("CTR", "LUAD", "LUAD", "LUSC")
)
P2_EPI_NuclearData$Subtype2 <- factor(
  P2_EPI_NuclearData$Structure,
  levels = unique(P2_EPI_NuclearData$Structure)[c(2,4,5,3)],
  labels = c("CTR", "LUAD (WD)", "LUAD (PD)", "LUSC")
)
test_EPI <- P2_EPI_NuclearData 
test_EPI$Cell_Class <- "Unknown"
Intensity_cols <- names(test_EPI)[grep("Intensity", names(test_EPI), fixed = T)]
intensity.mtx <- as.matrix(t(test_EPI[, Intensity_cols]))
Coverage_cols <- names(test_EPI)[grep("Coverage", names(test_EPI), fixed = T)]
Coverage.mtx <- as.matrix(t(test_EPI[, Coverage_cols]))
intensity.mtx <- Coverage.mtx*intensity.mtx

rownames(Coverage.mtx) <- str_split_fixed(Intensity_cols, "_", 2)[,1]
colnames(Coverage.mtx) <- paste(test_EPI$Core, test_EPI$X.Center..Pxl., test_EPI$Y.Center..Pxl., sep = "_")
#str(Coverage.mtx)

binary_class <- as.data.frame(t(Coverage.mtx))

#calculate thresh as median + 2x MADs
thresh <- list()
for (i in names(binary_class)) {
  thresh[[i]] <- median(binary_class[,i]) + mad(binary_class[,i])
  if (thresh[[i]] < 0.2) {
    thresh[[i]] <- 0.2
  }
}
thresh <- unlist(thresh)
thresh
thresh[["PANCK"]] <- 0.2

for(i in 1:ncol(binary_class)) {
  binary_class[,i] <- binary_class[,i] > thresh[i]
}
binary_class$EPI <- binary_class$PANCK == T
binary_class$BM <- rowSums(binary_class[, c("S100A9","KRT17")]) > 0
binary_class$ALV <- rowSums(binary_class[, c("HOPX","SFTPD")]) > 0 
binary_class$ALL <- paste(binary_class$EPI, binary_class$BM, binary_class$ALV)
table(binary_class$ALL)
binary_class$Epi.class <- factor(
  binary_class$ALL,
  levels = c("TRUE FALSE FALSE", "TRUE FALSE TRUE", "TRUE TRUE FALSE", "TRUE TRUE TRUE"),
  labels = c("Undetermined", "ALV", "BM", "Undetermined"))

PhenoMarkers <- c("S100A9","KRT17", "MKI67", "SOX9", "HOPX", "SFTPD")
binary_class$Phenotype <- unlist(lapply(apply(binary_class[,PhenoMarkers],1, function(x) PhenoMarkers[which(x %in% T)]),
                                        function(x) paste(x, "+", sep = "", collapse = " ")))

binary_class$Phenotype <- factor(binary_class$Phenotype)

Spatial_data <- list()
Spatial_data[["Cell_class"]] <- binary_class
Spatial_data[["exprs"]] <- as.data.frame(t(Coverage.mtx))
Spatial_data[["input"]] <- test_EPI

set.seed(12)
DS_data <- Create_ds_dataset(x = Spatial_data, class.col = "Epi.class", omit = "Undetermined", prop = 0.05)
DS_data.umap <- umap::umap(DS_data$exprs[,c("S100A9","KRT17","HOPX", "SFTPD", "PANCK")])

DS_data$Cell_class <- 
  DS_data$Cell_class %>%  mutate(
    UMAP_1 = DS_data.umap$layout[,1],
    UMAP_2 = DS_data.umap$layout[,2],
    Phenotype_ALV.BM = factor(paste(SFTPD, HOPX, S100A9, KRT17)),
    Epi.class2 = factor(Epi.class, levels = c("ALV", "BM"), labels = c("ALV/Inflamed", "BM"))
  )

Figure_5D <- 
  DS_data$Cell_class %>% 
  ggplot(aes(x = UMAP_1, y = UMAP_2, colour = Epi.class2)) +
  theme_pubr(base_size = 7) +
  scattermore::geom_scattermore(pointsize = 2) +
  scale_color_manual(values = col_pal) +
  theme(legend.position = "right", legend.key.size = unit(2,"pt"), legend.title = element_blank())
Figure_5D
```

```
ggsave(Figure_5D, path = Plots_out, file = "Figure_5D.svg",
       width = 7, height = 4, units = "cm")

Figure_S5C <- 
  ggarrange(
  DS_data$Cell_class %>% 
    ggplot(aes(x = UMAP_1, y = UMAP_2, colour = HOPX)) +
    theme_pubr(base_size = 7) +
    scattermore::geom_scattermore(pointsize = 2) +
    scale_colour_viridis_d() + 
    theme(legend.key.size = unit(2,"pt"), legend.title = element_blank()) +
    ggtitle("HOPX"),
  DS_data$Cell_class %>% 
    ggplot(aes(x = UMAP_1, y = UMAP_2, colour = SFTPD)) +
    theme_pubr(base_size = 7) +
    scattermore::geom_scattermore(pointsize = 2)+
    scale_colour_viridis_d()+ 
    theme(legend.key.size = unit(2,"pt"), legend.title = element_blank()) +
    ggtitle("SFTPD"),
  DS_data$Cell_class %>% 
    ggplot(aes(x = UMAP_1, y = UMAP_2, colour = S100A9)) +
    theme_pubr(base_size = 7) +
    scattermore::geom_scattermore(pointsize = 2)+
    scale_colour_viridis_d()+ 
    theme(legend.key.size = unit(2,"pt"), legend.title = element_blank()) +
    ggtitle("S100A9"),
  DS_data$Cell_class %>% 
    ggplot(aes(x = UMAP_1, y = UMAP_2, colour = KRT17)) +
    theme_pubr(base_size = 7) +
    scattermore::geom_scattermore(pointsize = 2)+
    scale_colour_viridis_d()+ 
    theme(legend.key.size = unit(2,"pt"), legend.title = element_blank()) +
    ggtitle("KRT17"),
  ncol = 4, nrow = 1, common.legend = T, legend = "right")

Figure_S5C
```

```
ggsave(Figure_S5C, path = Plots_out, file = "Figure_S5C.svg",
       width = 18, height = 4, units = "cm")
```

FIGURE 6E

```
P2_EPI_NuclearData$Epi.class <- binary_class$Epi.class
P2_EPI_NuclearData$Phenotype <- binary_class$Phenotype

#Chi sq residual analysis####
EPI_cells <- P2_EPI_NuclearData[P2_EPI_NuclearData$PANCK_Coverage.mod. > 0.2, ]
EPI_cells$Cell.type <- paste("EPI", EPI_cells$Epi.class, sep = "_")
EPI_cells$SampleID <- EPI_cells$`Mixed Lung TMA Yr 2023`

EPI.chi_sq <- chisq.test(table(
  EPI_cells$SampleID,
  EPI_cells$Cell.type
))
EPI_residuals <- as.data.frame(EPI.chi_sq$residuals)
EPI_residuals$SampleID <- rownames(EPI.chi_sq$residuals)
EPI_residuals <- merge(EPI_residuals, EPI_cells[!duplicated(EPI_cells$SampleID), c("Core", "Subtype", "Subtype2", "SampleID")],
                       by = "SampleID", all.x = T, all.y = F)


Figure_6e <- EPI_residuals %>%
  mutate(Cell_class2 = factor(Var2, levels = c("EPI_ALV", "EPI_BM", "EPI_Undetermined"),
                              labels = c("ALV/Inflamed\n(HOPX+|SFTPD+)", "BM\n(KRT17+|S100A9+)", "Undetermined"))) %>%
  dplyr::filter(!is.na(Subtype2) & !Cell_class2 == "Undetermined") %>%
  ggplot(aes(x = Subtype2, y = Freq, fill = Subtype2)) +
  theme_pubr(base_size = 7) +
  geom_boxplot(outlier.shape = NA, show.legend = F) +
  geom_jitter(width = 0.2, size = 0.5, show.legend = F) +
  facet_wrap(~Cell_class2) +
  rotate_x_text(angle = 45) +
  ylab("Subpopulation enrichment\n(Pearson's residuals)") +
  ggpubr::geom_pwc(method = "wilcox_test", label = "p.adj.signif", p.adjust.method = "fdr", hide.ns = T, tip.length = 0, label.size = 2, vjust = 0.5) +
  scale_fill_manual(values = c("forestgreen", "darkorange", "darkorange4", "skyblue1")) + theme(axis.title.x = element_blank())
Figure_6e
```

```
ggsave(Figure_6e, path = Plots_out, file = "Figure_6e.svg",
       width = 6, height = 4, units = "cm")
```

Figure S6B

```
# LUSC
LUSC_all <- merge(Merged.LUSCtraits, merged_ALV.BM, by = "Sample.ID")
LUSC_all$Grade2 <- factor(LUSC_all$Grade, levels = c("G1", "well","G2", "mod", "G3", "poor"), labels = c("G1", "G1", "G2", "G2","G3", "G3"))

Figure_S6b <- LUSC_all %>% drop_na(Grade2) %>%
  ggplot(aes(x = Grade2, y = BM, fill = Grade2)) +
  theme_pubr(base_size = 7) +
  geom_jitter(width = 0.2, size = 0.5, alpha = 0.5) +
  geom_boxplot(outlier.shape = NA) +
  facet_wrap(~Dataset, scales = "free_y") +
  ggpubr::geom_pwc(method = "wilcox_test", p.adjust.method = "fdr",label = "p.adj.signif", vjust = 0.25, hide.ns = F, label.size = 2, tip.length = 0) +
  scale_fill_manual(values = c("#794DFF", "#FFD3C4", "#FF6341")) +
  theme(legend.position = "bottom", axis.text.x = element_blank(), axis.title.x = element_blank(), legend.margin = margin(t=-5), legend.title = element_blank()) +
  ylab("BM (ssGSEA score)") + 
  ggtitle("LUSC Grade")
Figure_S6b
```

```
ggsave(Figure_S6b, path = Plots_out, file = "Figure_S6b.svg",
       width = 7, height = 4.5, units = "cm")
```

FIGURE S6D

```
EPI_residuals_wide <- dcast(EPI_residuals, Var1 ~ Var2, value.var = "Freq")

#Plot showing increased MKI67 in BM+ cells###

MKI67_pos.test <- 
  EPI_cells %>%
  mutate(MKI67_pos = MKI67_Coverage > 0.2) %>%
  dplyr::select(Epi.class, SampleID, MKI67_pos) %>%
  table
MKI67_pos.test <- 
  as.data.frame(MKI67_pos.test) %>% group_by(SampleID, Epi.class) %>% mutate(percent = prop.table(Freq))
MKI67_pos.test <- merge(MKI67_pos.test, EPI_cells[!duplicated(EPI_cells$SampleID), c("SampleID", "Subtype2", "Subtype")],
                        by = "SampleID")


Figure_S6d <- MKI67_pos.test %>%
  dplyr::filter(MKI67_pos == T & !Epi.class == "Undetermined") %>%
  ggplot(aes(x = Epi.class, y = percent*100, fill = factor(Epi.class, labels = c("ALV/Inflamed", "BM")))) +
  theme_pubr(base_size = 7) +
  facet_wrap(~Subtype2, ncol = 4) +
  geom_boxplot(outlier.shape = NA) +
  geom_jitter(width = 0.2, size = 0.5, show.legend = F) +
  ggpubr::geom_pwc(method = "wilcox_test", label = "p.adj.signif", p.adjust.method = "fdr", label.size = 2, tip.length = 0, vjust = 0.5, hide.ns = T) +
  ylab("MKI67+ (% of subpopulation)") + scale_fill_manual(values = col_pal) +
  theme(axis.title.x = element_blank(), axis.text.x = element_blank(), legend.title = element_blank(), legend.position = "bottom", legend.margin = margin(t=-5))
  
Figure_S6d
```

```
ggsave(Figure_S6d, path = Plots_out, file = "Figure_S6d.svg",
       width = 6, height = 4, units = "cm")
```

FIGURE S6E

```
SOX9_pos.test <- 
  EPI_cells %>%
  mutate(SOX9_pos = SOX9_Coverage.mod. > 0.2) %>%
  dplyr::select(Epi.class, SampleID, SOX9_pos) %>%
  table
SOX9_pos.test <- 
  as.data.frame(SOX9_pos.test) %>% group_by(SampleID, Epi.class) %>% mutate(percent = prop.table(Freq))
SOX9_pos.test <- merge(SOX9_pos.test, EPI_cells[!duplicated(EPI_cells$SampleID), c("SampleID", "Subtype2", "Subtype")],
                       by = "SampleID")


Figure_S6e <- SOX9_pos.test %>%
  dplyr::filter(SOX9_pos == T & !Epi.class == "Undetermined") %>%
  ggplot(aes(x = Epi.class, y = percent*100, fill = factor(Epi.class, labels = c("ALV/Inflamed", "BM")))) +
  theme_pubr(base_size = 7) +
  facet_wrap(~Subtype2, ncol = 4) +
  geom_boxplot(outlier.shape = NA) +
  geom_jitter(width = 0.2, size = 0.5, show.legend = F) +
  ggpubr::geom_pwc(method = "wilcox_test", label = "p.adj.signif", p.adjust.method = "fdr", label.size = 2, tip.length = 0, vjust = 0.5, hide.ns = T) +
  ylab("SOX9+ (% of subpopulation)") + scale_fill_manual(values = col_pal) +
  theme(axis.title.x = element_blank(), axis.text.x = element_blank(), legend.title = element_blank(), legend.position = "bottom", legend.margin = margin(t=-5))
Figure_S6e
```

```
ggsave(Figure_S6e, path = Plots_out, file = "Figure_S6e.svg",
       width = 6, height = 4, units = "cm")
```

## Session Info

```
print(sessionInfo(), RNG = TRUE, locale = FALSE)
```

```
## R version 4.4.0 (2024-04-24 ucrt)
## Platform: x86_64-w64-mingw32/x64
## Running under: Windows 10 x64 (build 19045)
## 
## Matrix products: default
## 
## 
## Random number generation:
##  RNG:     Mersenne-Twister 
##  Normal:  Inversion 
##  Sample:  Rejection 
##  
## attached base packages:
## [1] stats     graphics  grDevices utils     datasets  methods   base     
## 
## other attached packages:
##  [1] Seurat_5.2.1        SeuratObject_5.0.2  sp_2.2-0           
##  [4] stringr_1.5.1       ggpubr_0.6.0        ggplot2_3.5.1      
##  [7] randomcoloR_1.1.0.1 maditr_0.8.5        tidyr_1.3.1        
## [10] GSVA_2.0.5         
## 
## loaded via a namespace (and not attached):
##   [1] RcppAnnoy_0.0.22            splines_4.4.0              
##   [3] later_1.4.1                 tibble_3.2.1               
##   [5] polyclip_1.10-7             graph_1.84.1               
##   [7] XML_3.99-0.18               fastDummies_1.7.5          
##   [9] lifecycle_1.0.4             rstatix_0.7.2              
##  [11] globals_0.16.3              lattice_0.22-6             
##  [13] MASS_7.3-64                 backports_1.5.0            
##  [15] magrittr_2.0.3              plotly_4.10.4              
##  [17] sass_0.4.9                  rmarkdown_2.29             
##  [19] jquerylib_0.1.4             yaml_2.3.10                
##  [21] httpuv_1.6.15               sctransform_0.4.1          
##  [23] askpass_1.2.1               spam_2.11-1                
##  [25] spatstat.sparse_3.1-0       reticulate_1.40.0          
##  [27] cowplot_1.1.3               pbapply_1.7-2              
##  [29] DBI_1.2.3                   RColorBrewer_1.1-3         
##  [31] abind_1.4-8                 zlibbioc_1.52.0            
##  [33] Rtsne_0.17                  GenomicRanges_1.58.0       
##  [35] purrr_1.0.4                 BiocGenerics_0.52.0        
##  [37] GenomeInfoDbData_1.2.13     IRanges_2.40.1             
##  [39] S4Vectors_0.44.0            ggrepel_0.9.6              
##  [41] irlba_2.3.5.1               spatstat.utils_3.1-2       
##  [43] listenv_0.9.1               umap_0.2.10.0              
##  [45] goftest_1.2-3               RSpectra_0.16-2            
##  [47] spatstat.random_3.3-2       annotate_1.84.0            
##  [49] fitdistrplus_1.2-2          parallelly_1.42.0          
##  [51] svglite_2.2.2               codetools_0.2-20           
##  [53] DelayedArray_0.32.0         tidyselect_1.2.1           
##  [55] UCSC.utils_1.2.0            farver_2.1.2               
##  [57] ScaledMatrix_1.14.0         spatstat.explore_3.3-4     
##  [59] matrixStats_1.5.0           stats4_4.4.0               
##  [61] jsonlite_1.8.9              progressr_0.15.1           
##  [63] Formula_1.2-5               ggridges_0.5.6             
##  [65] survival_3.8-3              systemfonts_1.3.1          
##  [67] tools_4.4.0                 ragg_1.5.0                 
##  [69] ica_1.0-3                   Rcpp_1.0.14                
##  [71] glue_1.7.0                  gridExtra_2.3              
##  [73] SparseArray_1.6.1           xfun_0.50                  
##  [75] MatrixGenerics_1.18.1       GenomeInfoDb_1.42.3        
##  [77] dplyr_1.1.4                 HDF5Array_1.34.0           
##  [79] withr_3.0.2                 fastmap_1.2.0              
##  [81] rhdf5filters_1.18.0         openssl_2.3.2              
##  [83] digest_0.6.35               rsvd_1.0.5                 
##  [85] R6_2.5.1                    mime_0.12                  
##  [87] textshaping_1.0.0           colorspace_2.1-1           
##  [89] scattermore_1.2             tensor_1.5                 
##  [91] spatstat.data_3.1-4         RSQLite_2.3.9              
##  [93] generics_0.1.3              data.table_1.15.4          
##  [95] httr_1.4.7                  htmlwidgets_1.6.4          
##  [97] S4Arrays_1.6.0              uwot_0.2.2                 
##  [99] pkgconfig_2.0.3             gtable_0.3.6               
## [101] blob_1.2.4                  lmtest_0.9-40              
## [103] SingleCellExperiment_1.28.1 XVector_0.46.0             
## [105] htmltools_0.5.8.1           carData_3.0-5              
## [107] dotCall64_1.2               GSEABase_1.68.0            
## [109] scales_1.3.0                Biobase_2.66.0             
## [111] png_0.1-8                   SpatialExperiment_1.16.0   
## [113] spatstat.univar_3.1-1       knitr_1.49                 
## [115] rstudioapi_0.17.1           reshape2_1.4.4             
## [117] rjson_0.2.23                nlme_3.1-167               
## [119] curl_6.2.0                  cachem_1.1.0               
## [121] zoo_1.8-12                  rhdf5_2.50.2               
## [123] KernSmooth_2.23-26          parallel_4.4.0             
## [125] miniUI_0.1.1.1              AnnotationDbi_1.68.0       
## [127] pillar_1.10.1               grid_4.4.0                 
## [129] vctrs_0.6.5                 RANN_2.6.2                 
## [131] promises_1.3.2              car_3.1-3                  
## [133] BiocSingular_1.22.0         beachmat_2.22.0            
## [135] xtable_1.8-4                cluster_2.1.8              
## [137] evaluate_1.0.3              magick_2.8.5               
## [139] cli_3.6.2                   compiler_4.4.0             
## [141] rlang_1.1.4                 crayon_1.5.3               
## [143] future.apply_1.11.3         ggsignif_0.6.4             
## [145] labeling_0.4.3              plyr_1.8.9                 
## [147] stringi_1.8.4               deldir_2.0-4               
## [149] viridisLite_0.4.2           BiocParallel_1.40.0        
## [151] munsell_0.5.1               Biostrings_2.74.1          
## [153] lazyeval_0.2.2              spatstat.geom_3.3-5        
## [155] V8_8.0.1                    Matrix_1.7-2               
## [157] RcppHNSW_0.6.0              patchwork_1.3.0            
## [159] sparseMatrixStats_1.18.0    bit64_4.6.0-1              
## [161] future_1.34.0               Rhdf5lib_1.28.0            
## [163] KEGGREST_1.46.0             shiny_1.10.0               
## [165] SummarizedExperiment_1.36.0 ROCR_1.0-11                
## [167] igraph_2.1.4                broom_1.0.7                
## [169] memoise_2.0.1               bslib_0.9.0                
## [171] bit_4.5.0.1
```
