# Supplementary material for: Developmental programmes drive cellular plasticity, disease progression and therapy resistance in lung adenocarcinoma
Source: Mol Oncol. 2026 May 27:10.1002/1878-0261.70263. Online ahead of print. doi: 10.1002/1878-0261.70263 (PMC13398952; doi:10.1002/1878-0261.70263)
Supplement: Supplementary file 1 — File 1. R Markdown HTML reports. [file MOL2-9999-0-s006.zip › Bienkowska_etal_MolOnc_Fig7.html]

Developmental programmes drive cellular plasticity, disease progression and therapy resistance in lung adenocarcinoma


# Developmental programmes drive cellular plasticity, disease progression and therapy resistance in lung adenocarcinoma

### Figure 7 - Type I interferon promotes BM activation in TP53-mutant LUAD

#### Kamila J Bienkowska, Stephany Gallardo Y, Nur S Zainal, Leena Arora, Matthew Ellis, Maria-Antoinette Lopez, Judith Austine, Sai Pittla, Serena J Chee, Aiman Alzetani, Emily C Shaw, Christian H Ottensmeier, Gareth J Thomas, Christopher J Hanley

#### 2025-11-18

Load packages

```
library(clusterProfiler)
library(ggplot2)
library(ggpubr)
library(dplyr)
library(nichenetr)
library(AnnotationDbi)
library(org.Mm.eg.db)
library(org.Hs.eg.db)
library(ReactomePA)
library(enrichplot)
library(dynamicTreeCut)
library(stringr)
library(edgeR)
library(Seurat)
library(reactome.db)
library(readxl)
library(ggpubr)
library(tidyverse)
library(reshape2)
library(rstatix)
library(gtools)
```

Load objects

```
setwd(input_files)
DEGs <- read.csv(file = "TCGA_LUAD_DEGs_BMhigh_vs_BMlow.csv")
load(file = "MicroarrayData.RData")
load(file = "Merged_LUAD_traits.Rdata")
load("NSCLC_integrated_epi.Rdata")
InVitro_data_H441vA549.IFN <- read_excel("InVitro_data.xlsx", sheet = "H441vA549 Data")
InVitro_data_3D.IFN.doseR <- read_excel("InVitro_data.xlsx", sheet = "3D IFN data")
InVitro_data_2Dv3D.IFN <- read_excel("InVitro_data.xlsx", sheet = "2Dv3D IFN Data")
```

Pathway enrichment based on DEGs in BM-high vs BM-low (REACTOME and
TCGA)

```
DEGs$sig.Rank <- DEGs$log2FoldChange * (1-DEGs$padj)
names(DEGs)[names(DEGs) == 'gene_symbol'] <- 'Gene'

gene.df <- bitr(DEGs$Gene, fromType = "SYMBOL",
                toType = c("SYMBOL", "ENTREZID"),
                OrgDb = org.Hs.eg.db)
DEGs.mapped <- merge(DEGs, gene.df, by.x = "Gene", by.y = "SYMBOL", all.x = F, all.y = F)
DEGs.mapped <- DEGs.mapped[!duplicated(DEGs.mapped$Gene), ]
geneList <- DEGs.mapped$sig.Rank[order(-DEGs.mapped$sig.Rank)]
names(geneList) <- DEGs.mapped$ENTREZID[order(-DEGs.mapped$sig.Rank)]

GSEA_REACTOME <- gsePathway(geneList, pvalueCutoff = 0.05)
GSEA_REACTOME <- setReadable(GSEA_REACTOME, OrgDb = org.Hs.eg.db, keyType="ENTREZID")
```

DEGs for microarray data (cutpoint based on 5Y-OS survival data) and
REACTOME

```
# GSE72094 ####
GSE72094_traits <- subset(merged_LUAD_traits, Dataset == "GSE72094")
GSE72094_traits$Group <- NA
GSE72094_traits$Group[GSE72094_traits$BM > 0.3143011] <- "high"
GSE72094_traits$Group[GSE72094_traits$BM < 0.3143011] <- "low"
GSE72094_traits$Group <- factor(GSE72094_traits$Group)


design <- model.matrix(~ 0 + factor(GSE72094_traits$Group))
colnames(design) <- levels(factor(GSE72094_traits$Group))
fit <- lmFit(GSE72094_matrix_filtered, design)
fit <- eBayes(fit)
results <- topTable(fit, adjust = "fdr", sort.by = "F", number = Inf)

contrast <- makeContrasts(high-low, levels = design)
fit2 <- contrasts.fit(fit, contrast)
fit2 <- eBayes(fit2)
GSE72094_DEGs <- topTable(fit2, adjust = "fdr", sort.by = "logFC", number = Inf)
GSE72094_DEGs$gene <- rownames(GSE72094_DEGs)
# some rows have more than one gene (they'll have the same statistic). We only keep the first gene
GSE72094_DEGs$Gene <- sapply(strsplit(GSE72094_DEGs$gene, " "), `[`, 1)
GSE72094_DEGs <- GSE72094_DEGs[-grep(" ", GSE72094_DEGs$gene, fixed = T),]

gene.df <- bitr(GSE72094_DEGs$Gene, fromType = "SYMBOL",
                toType = c("SYMBOL", "ENTREZID"),
                OrgDb = org.Hs.eg.db)
#head(gene.df)

GSE72094_DEGs.mapped <- merge(GSE72094_DEGs, gene.df, by.x = "Gene", by.y = "SYMBOL", all.x = F, all.y = F)
GSE72094_DEGs.mapped$sig.Rank <- GSE72094_DEGs.mapped$logFC * (1-GSE72094_DEGs.mapped$adj.P.Val)
table(duplicated(GSE72094_DEGs.mapped$Gene))
GSE72094_DEGs.mapped <- GSE72094_DEGs.mapped[!duplicated(GSE72094_DEGs.mapped$Gene), ]

# Okayama ####
Okayama_traits <- subset(merged_LUAD_traits, Dataset == "Okayama")
Okayama_traits$Group <- NA
Okayama_traits$Group[Okayama_traits$BM > 0.3036852] <- "high"
Okayama_traits$Group[Okayama_traits$BM < 0.3036852] <- "low"
Okayama_traits$Group <- factor(Okayama_traits$Group)

design <- model.matrix(~ 0 + factor(Okayama_traits$Group))
colnames(design) <- levels(factor(Okayama_traits$Group))
fit <- lmFit(Okayama_matrix_filtered, design)
fit <- eBayes(fit)
results <- topTable(fit, adjust = "fdr", sort.by = "F", number = Inf)

contrast <- makeContrasts(high-low, levels = design)
fit2 <- contrasts.fit(fit, contrast)
fit2 <- eBayes(fit2)
Okayama_DEGs <- topTable(fit2, adjust = "fdr", sort.by = "logFC", number = Inf)
Okayama_DEGs$gene <- rownames(Okayama_DEGs)

# some rows have more than one gene (they'll have the same statistic). We only keep the first gene
Okayama_DEGs$Gene <- sapply(strsplit(Okayama_DEGs$gene, " "), `[`, 1)
Okayama_DEGs <- Okayama_DEGs[-grep(" ", Okayama_DEGs$gene, fixed = T),]

gene.df <- bitr(Okayama_DEGs$Gene, fromType = "SYMBOL",
                toType = c("SYMBOL", "ENTREZID"),
                OrgDb = org.Hs.eg.db)
#head(gene.df)

Okayama_DEGs.mapped <- merge(Okayama_DEGs, gene.df, by.x = "Gene", by.y = "SYMBOL", all.x = F, all.y = F)
Okayama_DEGs.mapped$sig.Rank <- Okayama_DEGs.mapped$logFC * (1-Okayama_DEGs.mapped$adj.P.Val)

table(duplicated(Okayama_DEGs.mapped$Gene))
Okayama_DEGs.mapped <- Okayama_DEGs.mapped[!duplicated(Okayama_DEGs.mapped$Gene), ]


# Shedden ####
Shedden_traits <- subset(merged_LUAD_traits, Dataset == "Shedden")
Shedden_traits$Group <- NA
Shedden_traits$Group[Shedden_traits$BM > 0.04176004] <- "high"
Shedden_traits$Group[Shedden_traits$BM < 0.04176004] <- "low"
Shedden_traits$Group <- factor(Shedden_traits$Group)
levels(Shedden_traits$Group)

design <- model.matrix(~ 0 + factor(Shedden_traits$Group))
colnames(design) <- levels(factor(Shedden_traits$Group))
fit <- lmFit(Shedden_matrix_filtered, design)
fit <- eBayes(fit)
results <- topTable(fit, adjust = "fdr", sort.by = "F", number = Inf)


contrast <- makeContrasts(high-low, levels = design)
fit2 <- contrasts.fit(fit, contrast)
fit2 <- eBayes(fit2)
Shedden_DEGS <- topTable(fit2, adjust = "fdr", sort.by = "logFC", number = Inf)
Shedden_DEGS$gene <- rownames(Shedden_DEGS)
# some rows have more than one gene (they'll have the same statistic). We only keep the first gene
Shedden_DEGS$Gene <- sapply(strsplit(Shedden_DEGS$gene, " "), `[`, 1)
Shedden_DEGS <- Shedden_DEGS[-grep(" ", Shedden_DEGS$gene, fixed = T),]

gene.df <- bitr(Shedden_DEGS$Gene, fromType = "SYMBOL",
                toType = c("SYMBOL", "ENTREZID"),
                OrgDb = org.Hs.eg.db)
#head(gene.df)

Shedden_DEGs.mapped <- merge(Shedden_DEGS, gene.df, by.x = "Gene", by.y = "SYMBOL", all.x = F, all.y = F)
Shedden_DEGs.mapped$sig.Rank <- Shedden_DEGs.mapped$logFC * (1-Shedden_DEGs.mapped$adj.P.Val)

#table(duplicated(Shedden_DEGs.mapped$Gene))
Shedden_DEGs.mapped <- Shedden_DEGs.mapped[!duplicated(Shedden_DEGs.mapped$Gene), ]
#plot(Shedden_DEGs.mapped$sig.Rank[order(-Shedden_DEGs.mapped$sig.Rank)])
```

Combine results

```
#Combine results ####
DEGs_list <- list(
  TCGA = DEGs.mapped,
  GSE72094 = GSE72094_DEGs.mapped,
  Okayama = Okayama_DEGs.mapped,
  Shedden = Shedden_DEGs.mapped
)
for(i in names(DEGs_list)){
  rownames(DEGs_list[[i]]) <- DEGs_list[[i]]$Gene
  names(DEGs_list[[i]]) <- paste(names(DEGs_list[[i]]), i, sep = "_")
  DEGs_list[[i]]$Join = rownames(DEGs_list[[i]])
} 
DEGs_merge <- DEGs_list %>% purrr::reduce(left_join, by = "Join")

save(DEGs_merge, file = paste(input_files, "DEGs_merge.Rdata", sep = "/"))
```

Meta-analysis

```
# load(paste(input_files, "DEGs_merge.Rdata", sep = "/"))
DEGs_merge$meta.sigRank <- apply(
  DEGs_merge[, grep("sig.Rank", names(DEGs_merge), fixed = T)],
  1, sum
)

names(DEGs_merge)[c(4,15,25,35)]
DEGs_merge$median_logFC <- apply(DEGs_merge[,c("log2FoldChange_TCGA", "logFC_Shedden", "logFC_Okayama", "logFC_GSE72094")], 1, median)
meta_p <- list()
for(i in DEGs_merge$Gene_TCGA){
  adj.P_vals <- as.numeric(DEGs_merge[DEGs_merge$Gene_TCGA == i, c("padj_TCGA", "adj.P.Val_GSE72094", "adj.P.Val_Okayama", "adj.P.Val_Shedden")])
  sumlog_res <- metap::sumlog(na.omit(adj.P_vals))
  meta_p[[i]] <- sumlog_res$p
}
DEGs_merge$meta_adj.P <- unlist(meta_p)

Figure_7A <- 
  DEGs_merge %>%
  ggplot(aes(x = median_logFC, y = -log10(meta_adj.P), colour = meta.sigRank)) +
  theme_pubr(base_size = 7) +
  ggrastr::geom_point_rast(size = 0.1, raster.dpi = 512)+
  scale_color_viridis_c(name = "GSEA Ranking Metric") +
  theme(legend.position = c(0,1), legend.justification = c(-0.1,1), legend.key.width = unit(2,"pt"), legend.key.height = unit(5,"pt")) +
  xlab("Median Log2FC\n(BM-high vs -low)") + ylab("-log10(Sumlog meta adj.P)")
Figure_7A
```

```
ggsave(filename = "Figure_7A.svg", path = Plots_out,
       plot = Figure_7A, width = 5.5, height = 5.5, unit = "cm")

#GSEA
geneList <- DEGs_merge$meta.sigRank[order(-DEGs_merge$meta.sigRank)]
names(geneList) <- DEGs_merge$ENTREZID_TCGA[order(-DEGs_merge$meta.sigRank)]
geneList <- na.omit(geneList)
set.seed(123)
GSEA_REACTOME_meta <- gsePathway(geneList, pvalueCutoff = 0.05)
GSEA_REACTOME_meta <- setReadable(GSEA_REACTOME_meta, OrgDb = org.Hs.eg.db, keyType="ENTREZID")
```

```
# Group terms by core enrichment
create_LE.list <- function(GSEA_res){
  Terms = GSEA_res$ID
  LE = GSEA_res$core_enrichment
  
  LE.list = list()
  for(i in 1:length(Terms)){
    LE.list[[Terms[i]]] = unlist(strsplit(LE[i], "/", fixed = T))
  }
  return(LE.list)
}
LE_genes <- create_LE.list(GSEA_REACTOME_meta@result)

# Create Jaccard Overlap matrix
jaccard <- function(a, b) {
  shared <- length(intersect(a, b))
  union <- length(unique(c(a, b)))
  shared / union
}
term_IDs <- names(LE_genes)
jaccard_matrix <- outer(
  LE_genes, 
  LE_genes, 
  Vectorize(function(x,y) jaccard(x,y))
)
dimnames(jaccard_matrix) <- list(term_IDs, term_IDs)

# Visualise clustering 
# pheatmap::pheatmap(jaccard_matrix, clustering_method = "ward.D2", show_rownames = F, show_colnames = F, breaks = seq(0,0.5, length = 101))

term.clust <- hclust(dist(jaccard_matrix), method = "ward.D2")
term.kclust <- cutree(term.clust, k = 6)

term.clustering.df <- data.frame(
  row.names = rownames(jaccard_matrix),
  Term = rownames(jaccard_matrix),
  Kcluster = factor(term.kclust))
pheatmap::pheatmap(jaccard_matrix,
                   clustering_method = "ward.D2",
                   show_rownames = F, show_colnames = F,
                   annotation_row = term.clustering.df %>% dplyr::select(Kcluster),
                   breaks = seq(0,0.5, length = 101))
```

```
term.clustering.df$cluster2 <- factor(term.clustering.df$Kcluster, exclude = 3,
                                      levels = c(2,6,4,5,1), labels = c("C1","C1","C2","C3","C4"))

# pheatmap::pheatmap(jaccard_matrix, clustering_method = "ward.D2", show_rownames = F, show_colnames = F, annotation_row = term.clustering.df %>% dplyr::select(cluster2), breaks = seq(0,0.5, length = 101))

GSEA_REACTOME_results <- merge(GSEA_REACTOME_meta@result, term.clustering.df, by.x = "ID", by.y = "Term", all.x = T)
cluster.names <- 
  GSEA_REACTOME_results %>%
  filter(!is.na(cluster2)) %>%
  group_by(cluster2) %>%
  filter(setSize > 100) %>%
  top_n(1, -qvalue) %>%
  top_n(1, NES) %>%
  arrange(cluster2) %>%
  dplyr::select(Description)

term.clustering.df$Cluster.Core.term <- factor(term.clustering.df$cluster2, 
                                               levels = c(as.character(cluster.names$cluster2), NA),
                                               labels = c(cluster.names$Description, "other"), exclude = NULL)
GSEA_REACTOME_results$Cluster.Core.term <- factor(GSEA_REACTOME_results$cluster2, 
                                                  levels = c(as.character(cluster.names$cluster2), NA),
                                                  labels = c(cluster.names$Description, "other"), exclude = NULL)

pheatmap::pheatmap(jaccard_matrix,
                   clustering_method = "ward.D2",
                   show_rownames = F, show_colnames = F, 
                   breaks = seq(0,0.5, length = 101), fontsize = 7, 
                   treeheight_col = 10, treeheight_row = 0,
                   annotation_row = term.clustering.df %>% dplyr::select(Cluster.Core.term),
                   annotation_names_row = F, legend = T, annotation_legend = T)
```

```
Bulk_GSEA_heatmap.plot <- 
  pheatmap::pheatmap(jaccard_matrix,
                   clustering_method = "ward.D2",
                   show_rownames = F, show_colnames = F, 
                   breaks = seq(0,0.5, length = 101), fontsize = 7, 
                   treeheight_col = 10, treeheight_row = 0,
                   annotation_row = term.clustering.df %>% dplyr::select(Cluster.Core.term),
                   annotation_names_row = F, legend = F, annotation_legend = F, silent = T)

Bulk_GSEA_heatmap.plot.grobs <- 
  pheatmap::pheatmap(jaccard_matrix,
                     clustering_method = "ward.D2",
                     show_rownames = F, show_colnames = F, 
                     breaks = seq(0,0.5, length = 101), fontsize = 7, 
                     treeheight_col = 10, treeheight_row = 0,
                     annotation_row = term.clustering.df %>% dplyr::select(Cluster.Core.term),
                     annotation_names_row = F, silent = T)
Bulk_GSEA_heatmap.plot.legend <- Bulk_GSEA_heatmap.plot.grobs$gtable$grobs[[length(Bulk_GSEA_heatmap.plot.grobs$gtable$grobs)-1]]

#save the heatmap and legend separately as png and svg files respectively for memory efficiency
ggsave(filename = "Figure_7B.png", path = Plots_out, plot = Bulk_GSEA_heatmap.plot, width = 8, height = 8.5, unit = "cm")
ggsave(filename = "Figure_7B_legend.svg", path = Plots_out, plot = ggplotify::as.ggplot(Bulk_GSEA_heatmap.plot.legend), width = 5.5, height = 2.5, unit = "cm")
```

Bulk tissue GSEA plot for IFN signalling

```
#gsea results plotting ####
PW2p <- levels(GSEA_REACTOME_results$Cluster.Core.term)[3]
PW2p_ID <- GSEA_REACTOME_results$ID[GSEA_REACTOME_results$Description == PW2p]

Figure_7C <- gseaplot2(GSEA_REACTOME_meta, geneSetID = PW2p_ID,
                  title = "Antiviral mechanism by\nIFN-stimulated genes")
PW2p_data <- GSEA_REACTOME_results %>% filter(Description == PW2p)

Figure_7C[[1]] <- Figure_7C[[1]] + labs(subtitle = paste0("(NES=", signif(PW2p_data$NES,3), 
                                                "; FDR q=", signif(PW2p_data$qvalue,3), ")")) +
  ylab("Running\nES") + theme_pubr(base_size = 7) + theme(legend.position = "none")
Figure_7C[[3]] <- Figure_7C[[3]] + ylab("Ranking\nMetric") + theme_pubr(base_size = 7)
Figure_7C
```

```
ggsave(filename = "Figure_7C.svg", path = Plots_out,
       plot = Figure_7C, width = 5.5, height = 6, unit = "cm")
```

scRNAseq analysis - interferon pathway enrichment

```
DefaultAssay(Epi.integrated.filtered) <- "integrated"
Epi.integrated.filtered <-
  FindClusters(Epi.integrated.filtered,
               resolution = 0.05,
               verbose = FALSE)

Epi.integrated.filtered@meta.data$Epi_subpops <- 
  factor(Epi.integrated.filtered$integrated_snn_res.0.05,
         levels = 0:5,
         labels = c("Inflamed", "Basal", "AT2", "AT1", "Ciliated", "Club"))
Epi.integrated.filtered <- SetIdent(Epi.integrated.filtered, value = "Epi_subpops")

Epi.integrated.filtered$Subtype_Subpop <- paste(Epi.integrated.filtered$All_subtype,
                                                Epi.integrated.filtered$Epi_subpops,
                                                sep = "_")

Figure_7D <- 
  data.frame(Epi.integrated.filtered@meta.data, Epi.integrated.filtered@reductions$umap@cell.embeddings) %>%
  ggplot(aes(x = UMAP_1, y = UMAP_2)) +
  theme_pubr(base_size = 7) +
  scattermore::geom_scattermore(pointsize = 1) + 
  scattermore::geom_scattermore(data = data.frame(Epi.integrated.filtered@meta.data, Epi.integrated.filtered@reductions$umap@cell.embeddings) %>% filter(Subtype_Subpop %in% c("LUAD_Basal", "LUAD_AT2")),             aes(colour = Subtype_Subpop),pointsize = 2) + 
  theme(legend.position = c(1,1), legend.justification = c(1,1), legend.title = element_blank(), legend.key.size = unit(1,"pt")) +
  scale_colour_brewer(palette = "Set2")

Figure_7D
```

```
ggsave(filename = "Figure_7D.svg", path = Plots_out, plot = Figure_7D, width = 5.5, height = 5.5, unit = "cm")
```

```
#FindConservedMarkers
DefaultAssay(Epi.integrated.filtered) <- "RNA"

Epi.integrated.filtered <- SetIdent(Epi.integrated.filtered, value = "Subtype_Subpop")
Epi.integrated.filtered@meta.data %>%
  dplyr::select(Subtype_Subpop, Dataset) %>% table()

LUAD.Basal_v_LUAD.AT2_Markers <- FindConservedMarkers(
  Epi.integrated.filtered[, !Epi.integrated.filtered$Dataset == "TLDS"],
  ident.1 = "LUAD_Basal", ident.2 = "LUAD_AT2",
  grouping.var = "Dataset", min.pct = 0.1, 
  logfc.threshold = 0)
LUAD.Basal_v_LUAD.AT2_Markers[LUAD.Basal_v_LUAD.AT2_Markers == 0] <- .Machine$double.xmin
LUAD.Basal_v_LUAD.AT2_Markers <- 
  LUAD.Basal_v_LUAD.AT2_Markers %>%
  mutate(Czbiohub.sigRANK = Czbiohub_avg_log2FC * (1-Czbiohub_p_val_adj),
         Zilionis.sigRANK = Zilionis_avg_log2FC * (1-Zilionis_p_val_adj),
         Kim.sigRANK = Kim_avg_log2FC * (1-Kim_p_val_adj),
         Gene = rownames(LUAD.Basal_v_LUAD.AT2_Markers)
         )
LUAD.Basal_v_LUAD.AT2_Markers <- 
  LUAD.Basal_v_LUAD.AT2_Markers %>%
  mutate(meta.sigRANK = Czbiohub.sigRANK + Zilionis.sigRANK + Kim.sigRANK)
LUAD.Basal_v_LUAD.AT2_Markers$median_log2FC <- apply(
  LUAD.Basal_v_LUAD.AT2_Markers[, grep("avg_log2FC", names(LUAD.Basal_v_LUAD.AT2_Markers), fixed = T)],
  1,median
)
LUAD.Basal_v_LUAD.AT2_Markers$max_adj.P <- apply(
  LUAD.Basal_v_LUAD.AT2_Markers[, grep("p_val_adj", names(LUAD.Basal_v_LUAD.AT2_Markers), fixed = T)],
  1,max
)

# calculate meta p values
meta_p <- list()
for(i in LUAD.Basal_v_LUAD.AT2_Markers$Gene){
  sumlog_res <- metap::sumlog(LUAD.Basal_v_LUAD.AT2_Markers[i, grep("p_val_adj", names(LUAD.Basal_v_LUAD.AT2_Markers), fixed = T)])
  meta_p[[i]] <- sumlog_res$p
}
LUAD.Basal_v_LUAD.AT2_Markers$meta_adj.P <- unlist(meta_p)
gene.df <- bitr(LUAD.Basal_v_LUAD.AT2_Markers$Gene, fromType = "SYMBOL",
                toType = c("SYMBOL", "ENTREZID"),
                OrgDb = org.Hs.eg.db)
#head(gene.df)
LUAD.Basal_v_LUAD.AT2_Markers <- merge(LUAD.Basal_v_LUAD.AT2_Markers, gene.df, all.x = T, by.x = "Gene", by.y = "SYMBOL")

#plot(LUAD.Basal_v_LUAD.AT2_Markers$meta.sigRANK[order(-LUAD.Basal_v_LUAD.AT2_Markers$meta.sigRANK)])
geneList <- LUAD.Basal_v_LUAD.AT2_Markers$meta.sigRANK[order(-LUAD.Basal_v_LUAD.AT2_Markers$meta.sigRANK)]
names(geneList) <- LUAD.Basal_v_LUAD.AT2_Markers$ENTREZID[order(-LUAD.Basal_v_LUAD.AT2_Markers$meta.sigRANK)]
geneList <- na.omit(geneList)
geneList <- geneList[!is.na(names(geneList))]
#plot(geneList)
set.seed(123)
GSEA_REACTOME_sc <- gsePathway(geneList, pvalueCutoff = 1)
GSEA_REACTOME_sc <- setReadable(GSEA_REACTOME_sc, OrgDb = org.Hs.eg.db, keyType="ENTREZID")


# Example Reactome Pathway ID
pathway_id <- "R-HSA-909733"
# Get mapping of pathway IDs to Entrez gene IDs
path2gene <- as.list(reactomePATHID2EXTID)
# Retrieve genes for the given pathway
genes <- path2gene[[pathway_id]]

LUAD.Basal_v_LUAD.AT2_Markers$R_HSA_909733.genes <- LUAD.Basal_v_LUAD.AT2_Markers$ENTREZID %in% genes


# figure 7E
Figure_7E <- 
  LUAD.Basal_v_LUAD.AT2_Markers %>%
  ggplot(aes(x = median_log2FC, y = -log10(meta_adj.P))) +
  theme_pubr(base_size = 7) +
  geom_point(size = 0.1, colour = "grey50") +
  geom_point(data = LUAD.Basal_v_LUAD.AT2_Markers %>% filter(R_HSA_909733.genes == T), colour = "red", size = 0.5) +
  ggrepel::geom_text_repel(data = LUAD.Basal_v_LUAD.AT2_Markers %>% filter(R_HSA_909733.genes == T & median_log2FC > 1) %>% top_n(-meta_adj.P, n = 10), aes(label = Gene), size = 2, nudge_x = 7, fontface = "italic", xlim = c(0,9), min.segment.length = 0, max.overlaps = 100) +
  xlab("Median log2FC\n(LUAD-Basal vs LUAD-AT2)") + ylab("-log10(Sumlog meta adj.P)") + xlim(c(-10, 10))
Figure_7E
```

```
ggsave(filename = "Figure_7E.svg", path = Plots_out, plot = Figure_7E, width = 7.5, height = 5.5, unit = "cm")
```

Figure 7F

```
PW2p <- "Interferon alpha/beta signaling"
PW2p_ID <- GSEA_REACTOME_sc@result$ID[GSEA_REACTOME_sc@result$Description == PW2p]

Figure_7F <- gseaplot2(GSEA_REACTOME_sc, geneSetID = PW2p_ID,
                            title = PW2p)
PW2p_data <- GSEA_REACTOME_sc@result %>% filter(Description == PW2p)

Figure_7F[[1]] <- Figure_7F[[1]] + labs(subtitle = paste0("(NES=", signif(PW2p_data$NES,3), 
                                                                    "; p=", signif(PW2p_data$pvalue,3), ")")) +
  ylab("Running\nES") + theme_pubr(base_size = 7) + theme(legend.position = "none")
Figure_7F[[3]] <- Figure_7F[[3]] + ylab("Ranking\nMetric") + theme_pubr(base_size = 7)
Figure_7F
```

```
ggsave(filename = "Figure_7F.svg", path = Plots_out, plot = Figure_7F, width = 5.5, height = 5.5, unit = "cm")
```

Figure S7A

```
PW2p <- "Antiviral mechanism by IFN-stimulated genes"
PW2p_ID <- GSEA_REACTOME_sc@result$ID[GSEA_REACTOME_sc@result$Description == PW2p]

Figure_S7A <- gseaplot2(GSEA_REACTOME_sc, geneSetID = PW2p_ID,
                            title = PW2p)
PW2p_data <- GSEA_REACTOME_sc@result %>% filter(Description == PW2p)

Figure_S7A[[1]] <- Figure_S7A[[1]] + labs(subtitle = paste0("(NES=", signif(PW2p_data$NES,3), 
                                                                    "; p=", signif(PW2p_data$pvalue,3), ")")) +
  ylab("Running\nES") + theme_pubr(base_size = 7) + theme(legend.position = "none")
Figure_S7A[[3]] <- Figure_S7A[[3]] + ylab("Ranking\nMetric") + theme_pubr(base_size = 7)
Figure_S7A
```

```
ggsave(filename = "Figure_S7A.svg", path = Plots_out, plot = Figure_S7A, width = 9, height = 5.5, unit = "cm")
```

Figure 7H

```
#In vitro DATA ####

# 3D IFN dose response ####

#names(InVitro_data_3D.IFN.doseR)

InVitro_data_3D.IFN.doseR_long <- reshape2::melt(InVitro_data_3D.IFN.doseR, value.name = "RQ",
                                                 id.vars = c("substrate", "treatment"))

InVitro_data_H441vA549.IFN_long <- InVitro_data_H441vA549.IFN

names(InVitro_data_3D.IFN.doseR_long)
names(InVitro_data_H441vA549.IFN_long) <- make.names(names(InVitro_data_H441vA549.IFN_long))

combined_InVitroData <- rbind(
  InVitro_data_3D.IFN.doseR_long %>% filter(treatment == "1000 U/ml" & !variable == "KRT17") %>% mutate(Cell.line = "H441", Target.Name.x = variable) %>% select(Cell.line, treatment, Target.Name.x, RQ),
  InVitro_data_H441vA549.IFN_long %>% mutate(treatment = Treatment) %>% select(Cell.line, treatment, Target.Name.x, RQ)
)


DF.long.summary_H441vA549.IFN <-
  combined_InVitroData %>%
  group_by(Cell.line, treatment, Target.Name.x) %>%
  summarise(RQ.mean = mean(RQ, na.rm = T),
            RQ.sd = sd(RQ, na.rm = T),
            n = n()) %>%
  as.data.frame()
DF.long.summary_H441vA549.IFN$RQ.sem <- DF.long.summary_H441vA549.IFN$RQ.sd / sqrt(DF.long.summary_H441vA549.IFN$n) 
DF.long.summary_H441vA549.IFN$RQ <- DF.long.summary_H441vA549.IFN$RQ.mean

one.way_t.test <- combined_InVitroData %>% mutate(log2.RQ = log2(RQ)) %>% group_by(Cell.line, treatment, Target.Name.x) %>%  t_test(log2.RQ ~ 1, mu = 0) %>% as.data.frame()

DF.long.summary_H441vA549.IFN <- 
  DF.long.summary_H441vA549.IFN %>% mutate(long.ID = paste(Cell.line, treatment, Target.Name.x)) %>% as.data.frame()

one.way_t.test <- 
  one.way_t.test %>% mutate(long.ID = paste(Cell.line, treatment, Target.Name.x)) %>% as.data.frame()

DF.long.summary_H441vA549.IFN <- merge(
  DF.long.summary_H441vA549.IFN,
  one.way_t.test,
  by = "long.ID")

DF.long.summary_H441vA549.IFN$p.signif <- stars.pval(DF.long.summary_H441vA549.IFN$p)
DF.long.summary_H441vA549.IFN$Target.Name.x <- DF.long.summary_H441vA549.IFN$Target.Name.x.x
DF.long.summary_H441vA549.IFN$treatment <- DF.long.summary_H441vA549.IFN$treatment.x
DF.long.summary_H441vA549.IFN$Cell.line <- DF.long.summary_H441vA549.IFN$Cell.line.x
DF.long.summary_H441vA549.IFN$p.adj <- p.adjust(DF.long.summary_H441vA549.IFN$p, method = "fdr") 
DF.long.summary_H441vA549.IFN$p.adj.signif <- stars.pval(DF.long.summary_H441vA549.IFN$p.adj)

Figure_7H <- 
  DF.long.summary_H441vA549.IFN %>%
  ggplot(aes(x = factor(paste(Cell.line, treatment),
                        levels = c("A549 CTR", "H441 CTR", "A549 1000 U/ml", "H441 1000 U/ml"),
                        labels = c("CTR", "CTR", "A549 +IFNa", "H441 +IFNa")),
             y = log2(RQ))) +
  theme_pubr(base_size = 7) +
  geom_bar(stat = "identity", aes(fill = Cell.line)) + geom_errorbar(aes(ymin = log2(RQ-RQ.sem), ymax = log2(RQ+RQ.sem), width = 0.3)) +
  geom_jitter(data = combined_InVitroData ,
              aes(x = factor(paste(Cell.line, treatment),
                             levels = c("A549 CTR", "H441 CTR", "A549 1000 U/ml", "H441 1000 U/ml"),
                             labels = c("CTR", "CTR", "A549 +IFNa", "H441 +IFNa")),
                  y = log2(RQ)),
              width = 0.1, size = 0.5) +
  facet_grid(~Target.Name.x) +
  #scale_y_continuous(limits = c(0, NA)) +
  rotate_x_text(angle = 45) +
  xlab("IFN-alpha treatment") +
  theme(strip.text = element_text(face = "italic")) +
  geom_hline(yintercept = 0, linetype = "dotted") +
  geom_text(aes(label = p.adj.signif, y = 3), size = 2) +
  theme(strip.text = element_text(face = "italic")) +
  scale_fill_brewer(palette = "Set1", labels = c("A549 (TP53 wt)", "H441 (TP53 mut)")) +
  theme(legend.position = "right", legend.title = element_blank(), axis.title.x = element_blank(), legend.key.size = unit(5, "pt"))
Figure_7H
```

```
ggsave(filename = "Figure_7H.svg", path = Plots_out, plot = Figure_7H, width = 14, height = 5, unit = "cm")
```

Figure S7C

```
# 2D v 3D ####

#names(InVitro_data_2Dv3D.IFN)
InVitro_data_2Dv3D.IFN_long <- reshape2::melt(InVitro_data_2Dv3D.IFN, value.name = "RQ",
                                              id.vars = c("substrate", "treatment"))

#names(InVitro_data_3D.IFN.doseR_long)
#str(InVitro_data_3D.IFN.doseR_long)
InVitro_data_3D.IFN.doseR_long$treatment <- factor(InVitro_data_3D.IFN.doseR_long$treatment, levels = unique(InVitro_data_3D.IFN.doseR_long$treatment))

DF.long.summary_2Dv3D.IFN <-
  InVitro_data_2Dv3D.IFN_long %>%
  group_by(substrate, treatment, variable) %>%
  summarise(RQ.mean = mean(RQ, na.rm = T),
            RQ.sd = sd(RQ, na.rm = T),
            n = n()) %>%
  as.data.frame()
DF.long.summary_2Dv3D.IFN$RQ.sem <- DF.long.summary_2Dv3D.IFN$RQ.sd / sqrt(DF.long.summary_2Dv3D.IFN$n) 
DF.long.summary_2Dv3D.IFN$RQ <- DF.long.summary_2Dv3D.IFN$RQ.mean

Figure_S7C <- 
  DF.long.summary_2Dv3D.IFN %>%
  filter(!variable %in% c("SFTPD", "ABCA3", "HOPX")) %>%
  ggplot(aes(x = factor(paste(substrate, treatment),
                        levels = c("2D CTR", "3D CTR", "2D 1000 U/ml", "3D 1000 U/ml"),
                        labels = c("CTR", "CTR", "2D +IFNa", "3D +IFNa")), y = log2(RQ))) +
  theme_pubr(base_size = 7) +
  geom_bar(stat = "identity", aes(fill = substrate)) +
  geom_errorbar(aes(ymin = log2(RQ-RQ.sem), ymax = log2(RQ+RQ.sem), width = 0.3)) +
  geom_jitter(data = InVitro_data_2Dv3D.IFN_long %>% filter(!variable %in% c("SFTPD", "ABCA3", "HOPX")),
              aes(x = factor(paste(substrate, treatment),
                             levels = c("2D CTR", "3D CTR", "2D 1000 U/ml", "3D 1000 U/ml"),
                             labels = c("CTR", "CTR", "2D +IFNa", "3D +IFNa")),
                  y = log2(RQ)),
              width = 0.1, size = 0.5) +
  facet_grid(~variable) +
  #scale_y_continuous(limits = c(0, NA)) +
  geom_hline(yintercept = 0, linetype = "dotted") +
  rotate_x_text(angle = 45) +
  theme(strip.text = element_text(face = "italic")) +
  scale_fill_brewer(palette = "Set1") +
  theme(legend.position = "right", legend.title = element_blank(), axis.title.x = element_blank(), legend.key.size = unit(5, "pt"))
Figure_S7C
```

```
ggsave(filename = "Figure_S7C.svg", path = Plots_out, plot = Figure_S7C, width = 18, height = 5, unit = "cm")
```

Figure S7D

```
#In vitro DATA ####

# 3D IFN dose response ####

#names(InVitro_data_3D.IFN.doseR)

InVitro_data_3D.IFN.doseR_long <- reshape2::melt(InVitro_data_3D.IFN.doseR, value.name = "RQ",
                                                 id.vars = c("substrate", "treatment"))

#names(InVitro_data_3D.IFN.doseR_long)
#str(InVitro_data_3D.IFN.doseR_long)
InVitro_data_3D.IFN.doseR_long$treatment <- factor(InVitro_data_3D.IFN.doseR_long$treatment, levels = unique(InVitro_data_3D.IFN.doseR_long$treatment))

DF.long.summary_3D.IFN.doseR <-
  InVitro_data_3D.IFN.doseR_long %>%
  group_by(treatment, variable) %>%
  summarise(RQ.mean = mean(RQ, na.rm = T),
            RQ.sd = sd(RQ, na.rm = T),
            n = n()) %>%
  as.data.frame()
DF.long.summary_3D.IFN.doseR$RQ.sem <- DF.long.summary_3D.IFN.doseR$RQ.sd / sqrt(DF.long.summary_3D.IFN.doseR$n) 
DF.long.summary_3D.IFN.doseR$RQ <- DF.long.summary_3D.IFN.doseR$RQ.mean

one.way_t.test <- InVitro_data_3D.IFN.doseR_long %>% group_by(variable, treatment) %>% mutate(log2.RQ = log2(RQ)) %>% t_test(log2.RQ ~ 1, mu = 0) %>% as.data.frame()

DF.long.summary_3D.IFN.doseR <- 
  DF.long.summary_3D.IFN.doseR %>% mutate(long.ID.x = paste(variable, treatment)) %>% as.data.frame()
one.way_t.test <- 
  one.way_t.test %>% mutate(long.ID.y = paste(variable, treatment)) %>% as.data.frame()

DF.long.summary_3D.IFN.doseR <- merge(DF.long.summary_3D.IFN.doseR, one.way_t.test[, !names(one.way_t.test) %in% names(DF.long.summary_3D.IFN.doseR)],
                                      by.x = "long.ID.x", by.y = "long.ID.y")
DF.long.summary_3D.IFN.doseR$p.signif <- stars.pval(DF.long.summary_3D.IFN.doseR$p)
DF.long.summary_3D.IFN.doseR$p.adj <- p.adjust(DF.long.summary_3D.IFN.doseR$p, method = "fdr") 
DF.long.summary_3D.IFN.doseR$p.adj.signif <- stars.pval(DF.long.summary_3D.IFN.doseR$p.adj)

Figure_S7D <- 
  DF.long.summary_3D.IFN.doseR %>%
  ggplot(aes(x = treatment, y = log2(RQ))) +
  theme_pubr(base_size = 7) +
  geom_bar(stat = "identity", aes(fill = treatment)) +
  geom_errorbar(aes(ymin = log2(RQ-RQ.sem), ymax = log2(RQ+RQ.sem), width = 0.3)) +
  geom_jitter(data = InVitro_data_3D.IFN.doseR_long,
              aes(x = treatment, y = log2(RQ)), width = 0.1, size = 0.5) +
  facet_wrap(~variable, nrow = 1) +
  geom_hline(yintercept = 0, linetype = "dotted") +
  rotate_x_text(angle = 45) +
  geom_text(aes(label = p.adj.signif, y = 3), size = 2) +
  theme(strip.text = element_text(face = "italic"), axis.text.x = element_blank()) +
  scale_fill_brewer(palette = "Blues", name = "IFN-alpha treatment") +
  theme(legend.position = "bottom", axis.title.x = element_blank(), legend.margin = margin(t=-0.5), legend.key.size = unit(5, "pt"))
Figure_S7D
```

```
ggsave(filename = "Figure_S7D.svg", path = Plots_out, plot = Figure_S7D, width = 18, height = 5, unit = "cm")
```

## Session Info

```
print(sessionInfo(), RNG = TRUE, locale = FALSE)
```

```
## R version 4.4.0 (2024-04-24 ucrt)
## Platform: x86_64-w64-mingw32/x64
## Running under: Windows 10 x64 (build 19045)
## 
## Matrix products: default
## 
## 
## Random number generation:
##  RNG:     Mersenne-Twister 
##  Normal:  Inversion 
##  Sample:  Rejection 
##  
## attached base packages:
## [1] stats4    stats     graphics  grDevices utils     datasets  methods  
## [8] base     
## 
## other attached packages:
##  [1] gtools_3.9.5           rstatix_0.7.2          reshape2_1.4.4        
##  [4] lubridate_1.9.4        forcats_1.0.0          purrr_1.0.4           
##  [7] readr_2.1.5            tidyr_1.3.1            tibble_3.2.1          
## [10] tidyverse_2.0.0        readxl_1.4.3           reactome.db_1.89.0    
## [13] Seurat_5.2.1           SeuratObject_5.0.2     sp_2.2-0              
## [16] edgeR_4.4.2            limma_3.62.2           stringr_1.5.1         
## [19] dynamicTreeCut_1.63-1  enrichplot_1.26.6      ReactomePA_1.50.0     
## [22] org.Hs.eg.db_3.20.0    org.Mm.eg.db_3.20.0    AnnotationDbi_1.68.0  
## [25] IRanges_2.40.1         S4Vectors_0.44.0       Biobase_2.66.0        
## [28] BiocGenerics_0.52.0    nichenetr_2.2.1.1      dplyr_1.1.4           
## [31] ggpubr_0.6.0           ggplot2_3.5.1          clusterProfiler_4.14.4
## 
## loaded via a namespace (and not attached):
##   [1] R.methodsS3_1.8.2       nnet_7.3-20             goftest_1.2-3          
##   [4] Biostrings_2.74.1       TH.data_1.1-3           vctrs_0.6.5            
##   [7] ggtangle_0.0.6          spatstat.random_3.3-2   digest_0.6.35          
##  [10] png_0.1-8               shape_1.4.6.1           proxy_0.4-27           
##  [13] ggrepel_0.9.6           deldir_2.0-4            parallelly_1.42.0      
##  [16] MASS_7.3-64             httpuv_1.6.15           foreach_1.5.2          
##  [19] qvalue_2.38.0           withr_3.0.2             ggrastr_1.0.2          
##  [22] xfun_0.50               ggfun_0.1.8             survival_3.8-3         
##  [25] memoise_2.0.1           ggbeeswarm_0.7.2        gson_0.1.0             
##  [28] systemfonts_1.3.1       ragg_1.5.0              tidytree_0.4.6         
##  [31] zoo_1.8-12              GlobalOptions_0.1.2     pbapply_1.7-2          
##  [34] R.oo_1.27.0             Formula_1.2-5           KEGGREST_1.46.0        
##  [37] promises_1.3.2          httr_1.4.7              globals_0.16.3         
##  [40] fitdistrplus_1.2-2      rstudioapi_0.17.1       UCSC.utils_1.2.0       
##  [43] miniUI_0.1.1.1          generics_0.1.3          DOSE_4.0.0             
##  [46] base64enc_0.1-3         zlibbioc_1.52.0         ggraph_2.2.1           
##  [49] TFisher_0.2.0           polyclip_1.10-7         randomForest_4.7-1.2   
##  [52] GenomeInfoDbData_1.2.13 xtable_1.8-4            doParallel_1.0.17      
##  [55] evaluate_1.0.3          hms_1.1.3               irlba_2.3.5.1          
##  [58] colorspace_2.1-1        visNetwork_2.1.4        ROCR_1.0-11            
##  [61] reticulate_1.40.0       spatstat.data_3.1-4     magrittr_2.0.3         
##  [64] lmtest_0.9-40           later_1.4.1             viridis_0.6.5          
##  [67] ggtree_3.14.0           lattice_0.22-6          spatstat.geom_3.3-5    
##  [70] future.apply_1.11.3     scattermore_1.2         shadowtext_0.1.4       
##  [73] cowplot_1.1.3           matrixStats_1.5.0       RcppAnnoy_0.0.22       
##  [76] class_7.3-23            Hmisc_5.2-2             pillar_1.10.1          
##  [79] nlme_3.1-167            iterators_1.0.14        caTools_1.18.3         
##  [82] compiler_4.4.0          RSpectra_0.16-2         stringi_1.8.4          
##  [85] gower_1.0.2             tensor_1.5              plyr_1.8.9             
##  [88] crayon_1.5.3            abind_1.4-8             gridGraphics_0.5-1     
##  [91] sn_2.1.1                locfit_1.5-9.11         mathjaxr_1.6-0         
##  [94] graphlayouts_1.2.2      bit_4.5.0.1             sandwich_3.1-1         
##  [97] fastmatch_1.1-6         textshaping_1.0.0       multcomp_1.4-28        
## [100] codetools_0.2-20        recipes_1.1.0           bslib_0.9.0            
## [103] e1071_1.7-16            GetoptLong_1.0.5        plotly_4.10.4          
## [106] multtest_2.62.0         mime_0.12               splines_4.4.0          
## [109] circlize_0.4.16         Rcpp_1.0.14             fastDummies_1.7.5      
## [112] cellranger_1.1.0        knitr_1.49              blob_1.2.4             
## [115] clue_0.3-66             fs_1.6.5                listenv_0.9.1          
## [118] checkmate_2.3.2         Rdpack_2.6.2            ggsignif_0.6.4         
## [121] ggplotify_0.1.2         Matrix_1.7-2            statmod_1.5.0          
## [124] svglite_2.2.2           tzdb_0.4.0              pheatmap_1.0.12        
## [127] tweenr_2.0.3            pkgconfig_2.0.3         tools_4.4.0            
## [130] cachem_1.1.0            rbibutils_2.3           RSQLite_2.3.9          
## [133] numDeriv_2016.8-1.1     viridisLite_0.4.2       DBI_1.2.3              
## [136] graphite_1.52.0         fastmap_1.2.0           rmarkdown_2.29         
## [139] scales_1.3.0            grid_4.4.0              ica_1.0-3              
## [142] metap_1.11              broom_1.0.7             sass_0.4.9             
## [145] patchwork_1.3.0         dotCall64_1.2           graph_1.84.1           
## [148] carData_3.0-5           RANN_2.6.2              rpart_4.1.24           
## [151] snow_0.4-4              farver_2.1.2            tidygraph_1.3.1        
## [154] yaml_2.3.10             DiagrammeR_1.0.11       foreign_0.8-88         
## [157] cli_3.6.2               lifecycle_1.0.4         caret_7.0-1            
## [160] uwot_0.2.2              mvtnorm_1.3-3           presto_1.0.0           
## [163] lava_1.8.1              backports_1.5.0         BiocParallel_1.40.0    
## [166] timechange_0.3.0        gtable_0.3.6            rjson_0.2.23           
## [169] ggridges_0.5.6          progressr_0.15.1        parallel_4.4.0         
## [172] pROC_1.18.5             ape_5.8-1               jsonlite_1.8.9         
## [175] RcppHNSW_0.6.0          bitops_1.0-9            bit64_4.6.0-1          
## [178] qqconf_1.3.2            Rtsne_0.17              yulab.utils_0.2.0      
## [181] spatstat.utils_3.1-2    mutoss_0.1-13           jquerylib_0.1.4        
## [184] GOSemSim_2.32.0         spatstat.univar_3.1-1   R.utils_2.12.3         
## [187] timeDate_4041.110       lazyeval_0.2.2          shiny_1.10.0           
## [190] htmltools_0.5.8.1       GO.db_3.20.0            sctransform_0.4.1      
## [193] rappdirs_0.3.3          glue_1.7.0              spam_2.11-1            
## [196] XVector_0.46.0          treeio_1.30.0           mnormt_2.1.1           
## [199] gridExtra_2.3           igraph_2.1.4            R6_2.5.1               
## [202] fdrtool_1.2.18          labeling_0.4.3          cluster_2.1.8          
## [205] aplot_0.2.4             GenomeInfoDb_1.42.3     ipred_0.9-15           
## [208] vipor_0.4.7             plotrix_3.8-4           tidyselect_1.2.1       
## [211] htmlTable_2.4.3         ggforce_0.4.2           car_3.1-3              
## [214] future_1.34.0           ModelMetrics_1.2.2.2    munsell_0.5.1          
## [217] KernSmooth_2.23-26      data.table_1.15.4       htmlwidgets_1.6.4      
## [220] fgsea_1.32.2            ComplexHeatmap_2.22.0   RColorBrewer_1.1-3     
## [223] rlang_1.1.4             spatstat.sparse_3.1-0   spatstat.explore_3.3-4 
## [226] Cairo_1.7-0             ggnewscale_0.5.0        hardhat_1.4.1          
## [229] beeswarm_0.4.0          prodlim_2024.06.25
```
