# Supplementary material for: Developmental programmes drive cellular plasticity, disease progression and therapy resistance in lung adenocarcinoma
Source: Mol Oncol. 2026 May 27:10.1002/1878-0261.70263. Online ahead of print. doi: 10.1002/1878-0261.70263 (PMC13398952; doi:10.1002/1878-0261.70263)
Supplement: Supplementary file 2 — Fig. S1. Developmental Alveogenesis (ALV) and Branching Morphogenesis (BM) programmes are associated with transcriptomic variance in NSCLC. Fig. S2. High expression of BM in LUAD predicts poor survival and is associated with frequent TP53 mutations. Fig. S3. TP53 loss of function is required for BM activation. Fig. S4. High expression of BM is associated with resistance to therapies. Fig. S5. BM upregulation in LUAD is associated with acquisition of basal‐like features. Fig. S6. BM in LUAD is associated with high grade tumours. Fig. S7. Type I interferon promotes BM activation in TP53‐mutant LUAD. [file MOL2-9999-0-s001.pdf]

Developmental programmes drive cellular plasticity, disease progression and therapy resistance in lung adenocarcinoma (Supplementary Figures)

Kamila J Bienkowska[1], Stephany Gallardo[1], Nur S Zainal[1], Leena Arora[1], Matthew Ellis[1,2], Maria-Antoinette Lopez[2], Judith Austine[2], Sai Pittla[2], Serena J Chee[3], Aiman Alzetani[4], Emily C Shaw[1,2], Christian H Ottensmeier[3], Gareth J Thomas[1,2,5], Christopher J Hanley[1*]

Affiliations

[1] School of Cancer Sciences, University of Southampton, Southampton, UK, SO16 6YD

[2] Department of Histopathology, University Hospital Southampton NHS Foundation Trust, Southampton, UK, SO16 6YD.

[3] Institute of Systems, Molecular and Integrative Biology (ISMIB) and Liverpool Experimental Cancer Medicines Centre, University of Liverpool, Liverpool UK, L69 7BE.

[4] Department of Thoracic surgery, University Hospital Southampton NHS Foundation Trust, UK, SO16 6YD.

[5]Cancer Research UK and NIHR Southampton Experimental Cancer Medicine Centre, Southampton, UK, SO16 6YD.

*Corresponding Author: Dr. Christopher J. Hanley, C.j.hanley@soton.ac.uk

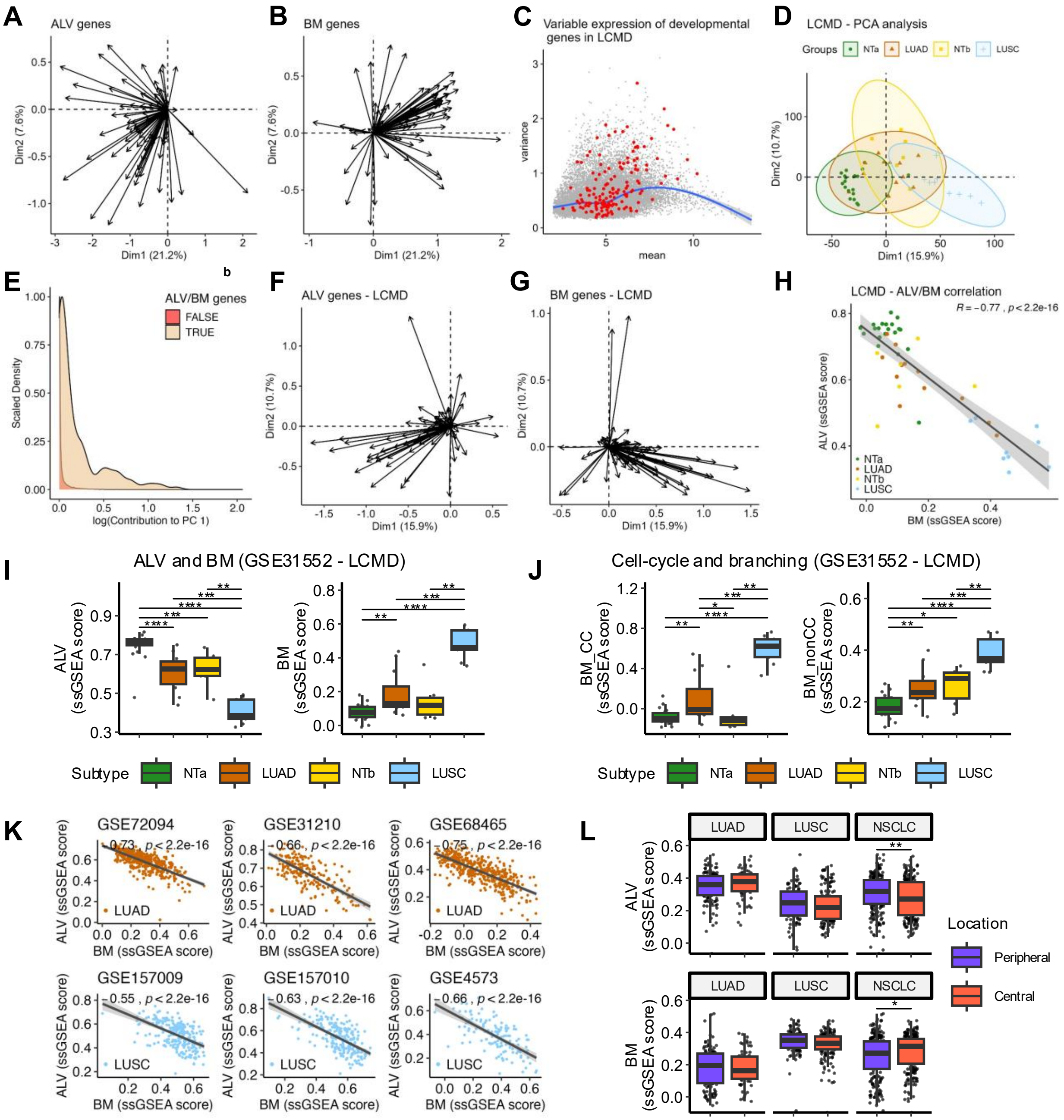

Figure S1. **Developmental Alveogenesis (ALV) and Branching Morphogenesis (BM) programmes are associated with transcriptomic variance in NSCLC. A-B** – PCA loading plots for ALV genes (A) and BM genes (B) in TCGA RNA-Seq data. **C** – Scatter plot showing mean expression levels and variance for all genes in NSCLC samples from Laser-Capture-Microdissection-Dataset (LCMD; GSE31552). BM and ALV genes are shown in red. **D-G** – PCA of NSCLC samples from LCMD. **D** – PC plot showing sample separation. **E** – Histogram showing the contribution of ALV/BM genes PC1 (LCMD). **F-G** - PCA loading plots for ALV genes (F) and BM genes (G) in LCMD. **H** – Scatter plot showing ssGSEA scores for ALV/BM programmes across LCMD samples. R = Spearman's correlation. **I-J**- Comparison of enrichment of ALV/BM (I) as well as BM_cc (cell-cycle related genes) and BM_nonCC (non-cell-cycle related genes) (J) across LCMD NSCLC samples. NTa – non-tumour alveolar, Ntb – non-tumour bronchial. **K** – Scatter

plot showing ALV/BM scores in LUAD/LUSC microarray datasets. LUAD: GSE72094 [2], GSE31210 [3], GSE68465 [4]. LUSC: GSE157009 [5], GSE157010 [5], GSE4573 [6]. R = Spearman's correlation. **L** - Comparison of ssGSEA scores for ALV/BM programmes between LUAD tumours, LUSC tumours, and NSCLC tumours (LUAD and LUSC combined) located in either peripheral or central parts of the lung (TCGA). Asterisks represent P values calculated with pairwise Wilcoxon rank-sum tests applying FDR correction where multiple tests were performed, non-significant comparisons ($p>0.05$) are not shown. NSCLC - non-small cell lung cancer, PCA - principal component analysis, PC1 - first principal component, LCMD - laser capture micro-dissection, LUSC - lung squamous cell carcinoma, LUAD - lung adenocarcinoma, TCGA - The Cancer Genome Atlas, FDR - false discovery rate, * – $p \leq 0.05$, ** – $p \leq 0.01$, *** – $p \leq 0.001$, **** – $p \leq 0.0001$.

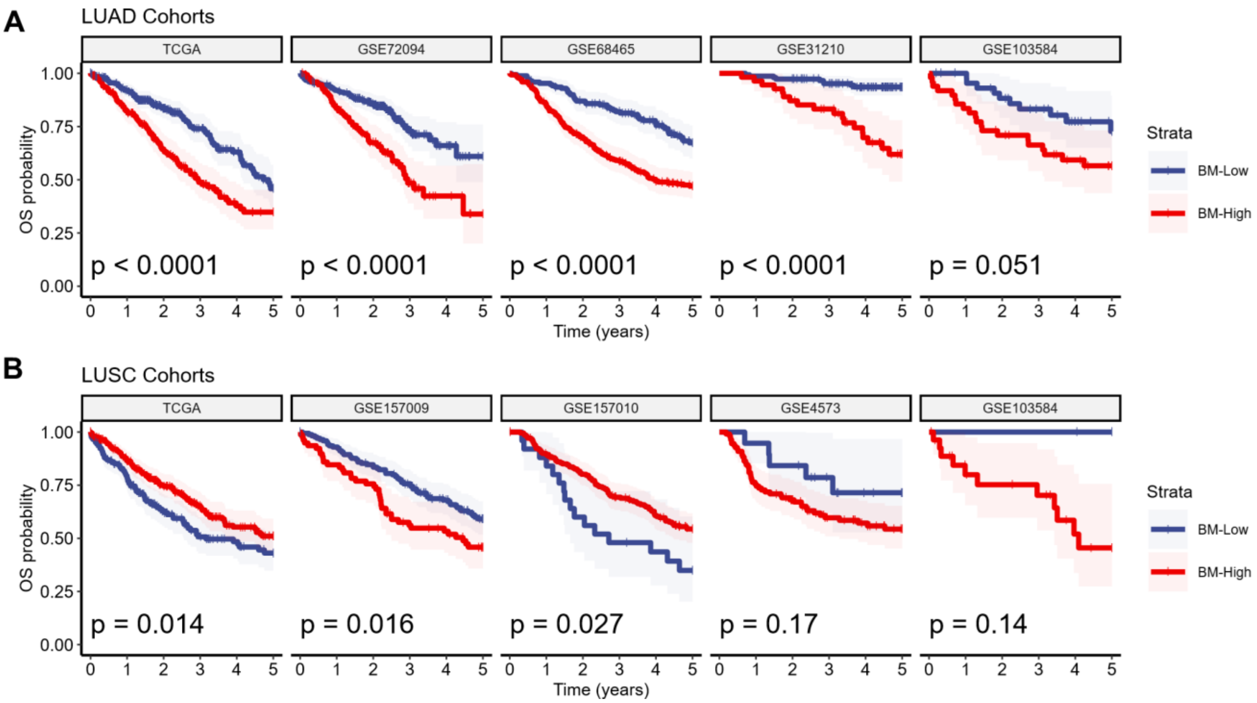

Figure S2. High expression of BM in LUAD predicts poor survival and is associated with frequent *TP53* mutations. A – KM plots showing 5-year overall survival (OS) probability based on BM expression across 5 LUAD datasets: TCGA, GSE72094, GSE68465, GSE31210, GSE103584. P values calculated with log-rank test; shaded region represents 95% CIs. **B** - KM plots showing 5-year overall survival (OS) based on BM expression across 5 LUSC datasets: TCGA, GSE157009, GSE157010, GSE4573, GSE103584. BM – branching morphogenesis, KM – Kaplan-Meier, TCGA – The Cancer Genome Atlas.

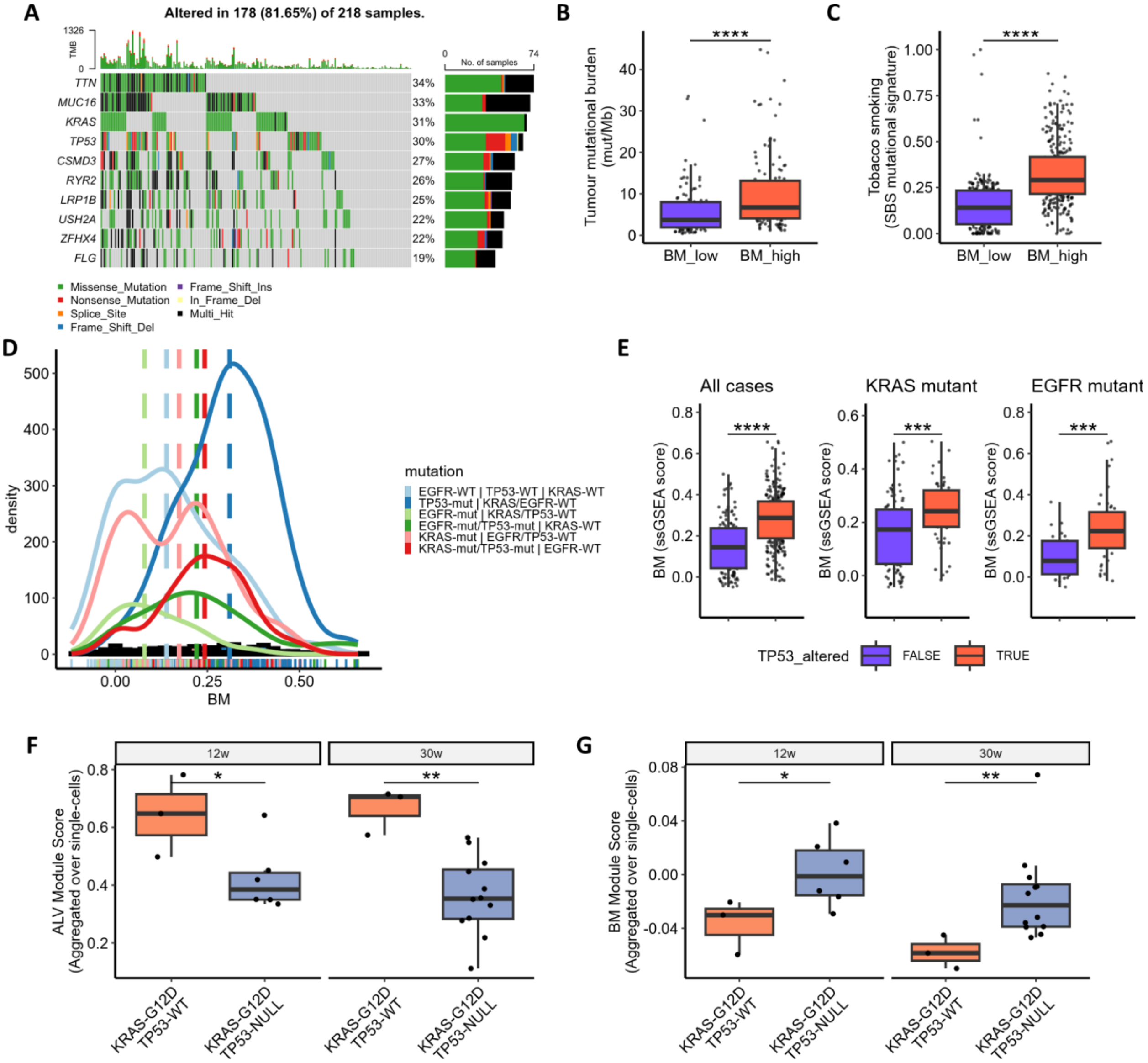

Figure S3. TP53 loss of function is required for BM activation. A – Oncoplot showing top 15 mutated genes in LUAD BM-low samples (TCGA). **B-C** – Boxplots showing the tumour mutational burden (B) and Tobacco smoking mutational signature (C) in LUAD (TCGA) samples split by BM status. **D** – Density plot showing the distribution of BM values in LUAD (TCGA) samples grouped by their *TP53*, *KRAS* and *EGFR* mutational status. **E** – Boxplots showing BM ssGSEA scores in the LUAD (TCGA samples) with the indicated *TP53*, *KRAS* and *EGFR* mutational status. True/False indicates whether a TP53 mutation was identified in the sample. **F-G** – Boxplots showing the ALV (F) and BM (G) scores for tumour cells collected from K (KrasG12D/+) or KP (KrasG12D/+; Trp53fl/fl) murine LUAD models, measured by scRNA-seq. Asterisks represent P values calculated with pairwise Wilcoxon rank-sum tests applying FDR correction where multiple tests were performed. BM – branching morphogenesis, TCGA – The Cancer Genome Atlas, ssGSEA – single-sample gene set enrichment analysis, FDR – false discovery rate, * – p ≤ 0.05, ** – p ≤ 0.01, *** – p ≤ 0.001, **** – p ≤ 0.0001.

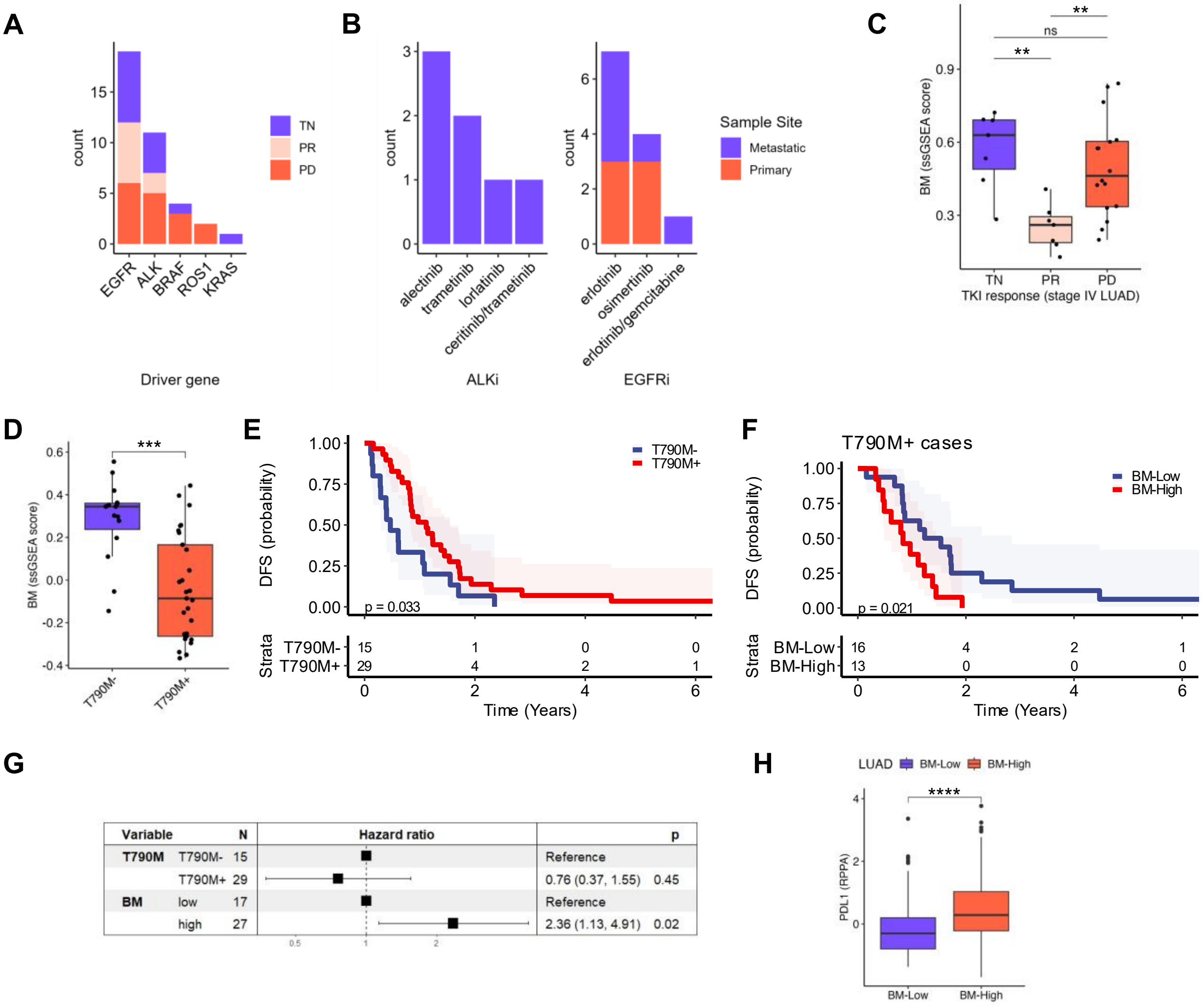

Figure S4. High expression of BM is associated with resistance to therapies. **A** – Barplot showing the number of samples analysed from the Maynard et al. cohort with the indicated driver gene and their associated grouping for analysis; TN – treatment naïve, PR – partial response, PD – progressive disease. **B** – Barplots showing the TKI treatment received for post-treatment samples with ALK or EGFR drivers. **C** – Boxplot showing the BM score for Stage IV samples only from the Maynard cohort. **D** – Boxplot showing BM scores in the IMPACT (Chua et al.) cohort grouped by T790M status. number of samples rec of post-treatment samples. **E** – Kaplan-Meier plot showing disease free survival (DFS) rates for the IMPACT cohort stratifying patients by T790M status. **F** – Kaplan-Meier plot showing DFS rates for T790M+ cases in the IMPACT cohort stratified by BM status. **G** – Forest plot showing multivariate regression analysis of the IMPACT cohort including T790M and BM status as independent variables. **H** - RPPA (reverse phase protein array) data of PD-L1 expression in LUAD TCGA samples grouped by BM expression. Asterisks represent P values calculated with pairwise Wilcoxon rank-sum tests applying FDR correction where multiple tests were performed. BM – branching morphogenesis, TKI – tyrosine kinase inhibitor, LUAD – lung adenocarcinoma, TCGA – The Cancer Genome Atlas, ** – p ≤ 0.01, *** – p ≤ 0.001, **** – p ≤ 0.0001.

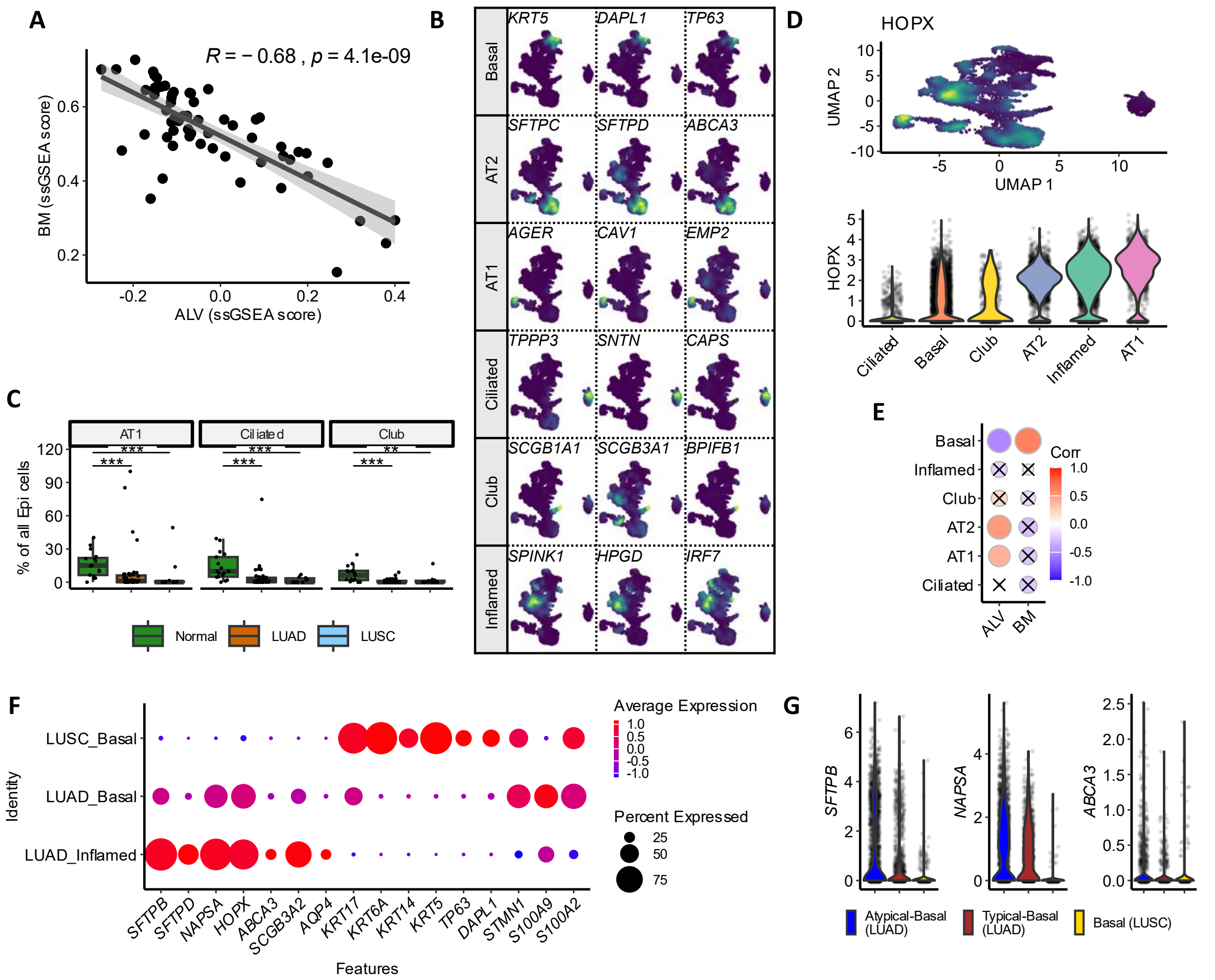

Figure S5. BM upregulation in LUAD is associated with acquisition of basal-like features. A – Scatter plot showing Pearson's correlation between ALV and BM scores in sample-level pseudobulk data from epithelial cells in the integrated scRNA-seq dataset. **B** – Feature density plots showing example marker genes for epithelial subpopulations. **C** – Boxplots showing the proportion of AT1, Ciliated and Club cell epithelial subpopulation across NSCLC sample types. **D** – Feature and Violin plots showing the expression of HOPX across epithelial cell subpopulations. **E -** Sample-level Pearson's correlation between ssGSEA scores for ALV/BM signatures and epithelial cluster abundance after excluding LUSC samples. P values > 0.01 are crossed out. **F –** Dot plot showing expression of alveolar and basal associated genes in LUAD-Inflamed, LUAD-Basal and LUSC-Basal cells. **G** – Violin plots showing expression of alveolar genes in LUAD and LUSC cells from the Basal cluster; LUAD cells with a basal prediction > 0.5 = "Typical basal cells"; < 0.5 = "Atypical basal cells". BM – branching morphogenesis, AT1 – alveolar type 1 cell, LUAD – lung adenocarcinoma, LUSC – lung squamous cell carcinoma, ssGSEA – single-sample gene set enrichment analysis, NSCLC – non-small cell lung cancer, ** – p ≤ 0.01, *** – p ≤ 0.001.

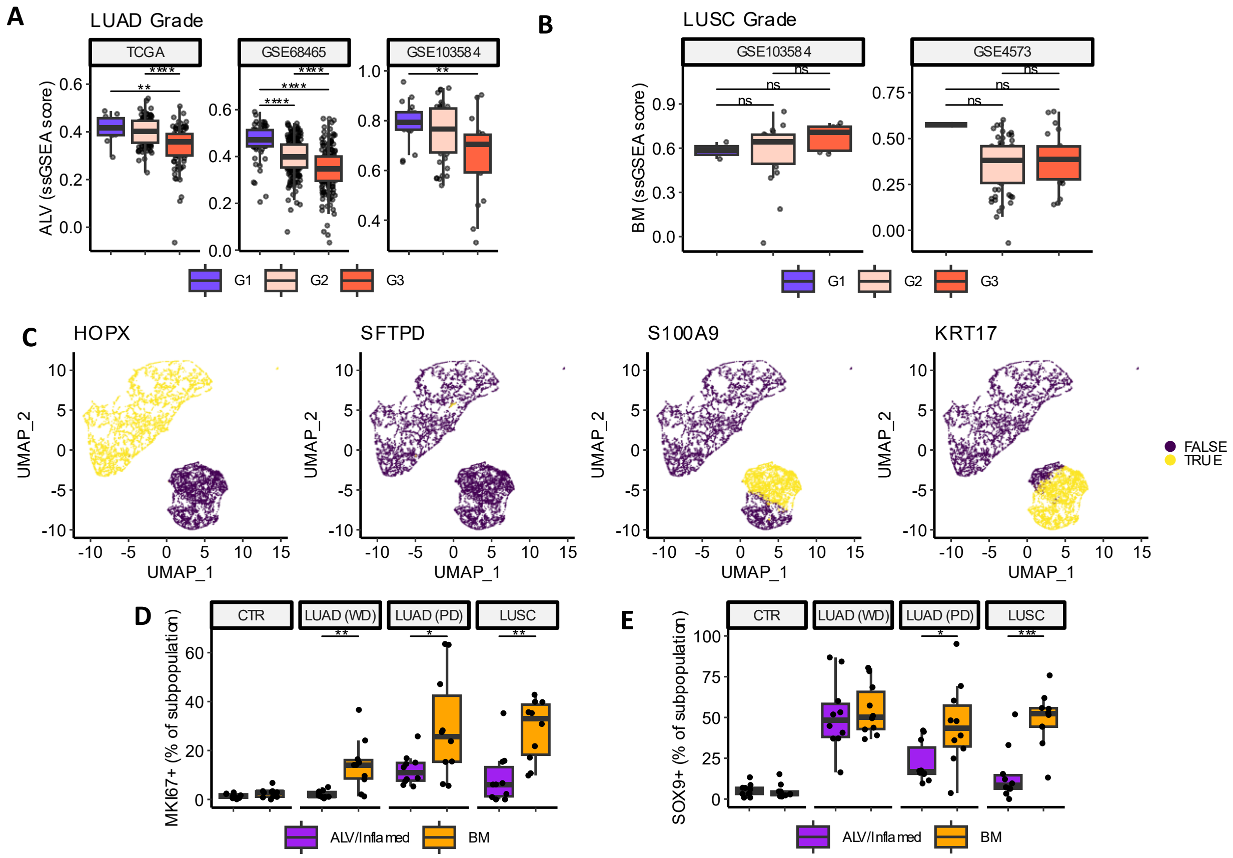

Figure S6. BM in LUAD is associated with high grade tumors. A - Boxplots showing ALV ssGSEA scores in 3 independent LUAD bulk transcriptomic datasets, grouping samples by histological grade (G1 - well differentiated, G2 - moderately differentiated, G3 - poorly differentiated). **B** – Boxplots showing BM ssGSEA scores in 2 independent LUSC bulk transcriptomic datasets, grouping samples by histological grade (p-values for all pairwise comparisons were >0.05). **C** – UMAP representation of NSCLC mxIHC epithelial (PANCK+) cells, coloured by positivity for the indicated marker. **D-E** - Boxplots showing the difference in percentage of MKI67-positive (D) and SOX9-positive epithelial cells (E) between BM population (S100A9+/KRT17+) and ALV/Inflamed population (SFTPD+/HOPX+) across NSCLC mxIHC samples. Asterisks represent P values calculated with pairwise Wilcoxon rank-sum tests applying FDR correction where multiple tests were performed. ALV – alveogenesis, BM – branching morphogenesis, mxIHC – multiplexed immunohistochemistry, LUSC – lung squamous cell carcinoma, LUAD – lung adenocarcinoma, * – p ≤ 0.05, ** – p ≤ 0.01, *** – p ≤ 0.001, **** – p ≤ 0.0001.

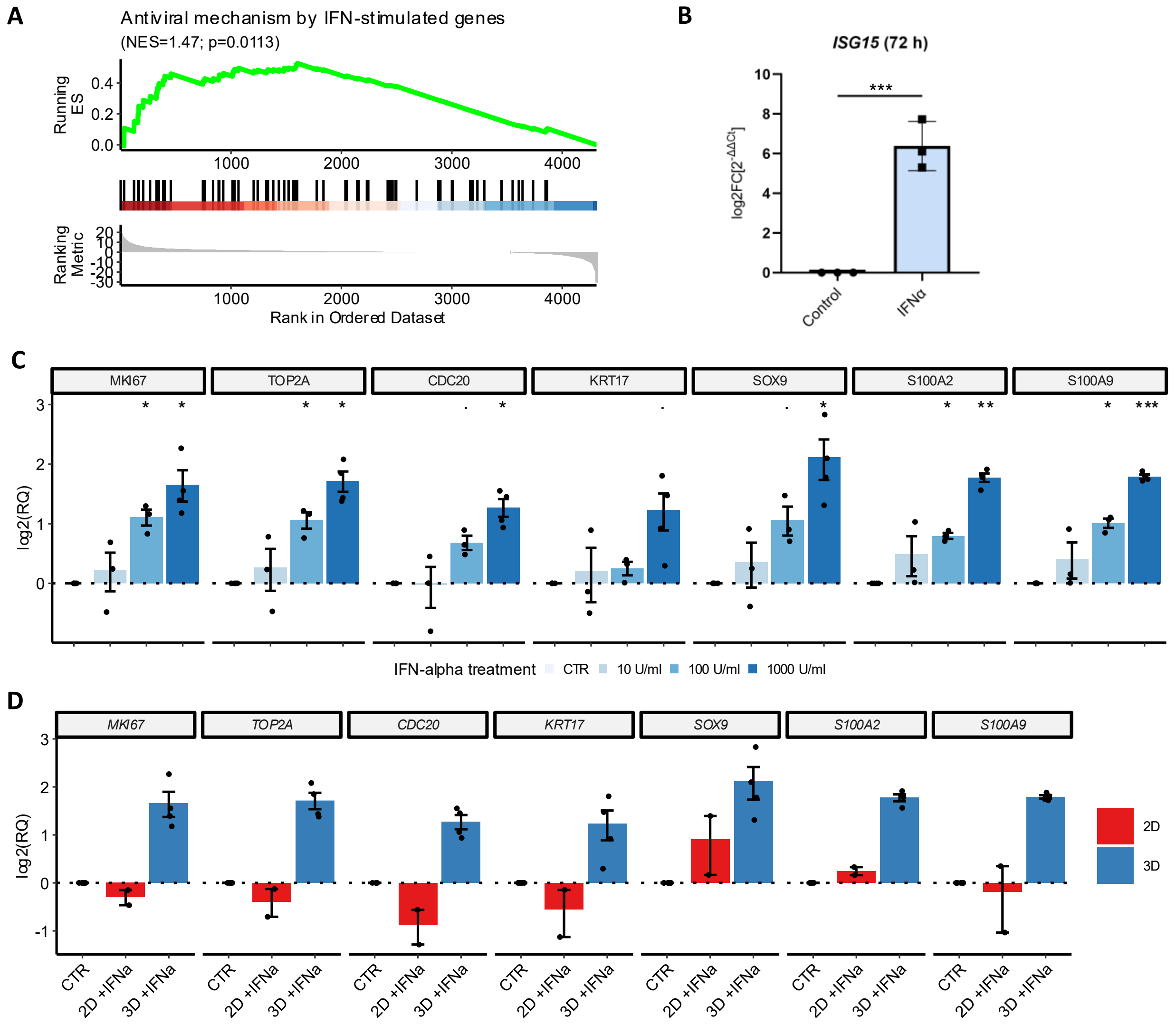

Figure S7. Type I interferon promotes BM activation in TP53-mutant LUAD. A - Gene set enrichment analysis (GSEA) of the "Antiviral mechanism by IFN-stimulated genes" pathway in LUAD_Basal vs LUAD_AT2 cells. **B** – Barplot showing qPCR results of *ISG15* expression in H322 spheroids treated with 1000 U/ml interferon alpha for 72 h. Asterisks represent p-value calculated with a two-sided Welch-test. **C** – Barplot showing mean log2(treatment vs. control relative quantity [RQ]) +/-SEM, from qPCR analysis of BM-associated genes in H322 spheroids treated with increasing concentrations of IFNα for 72 h (n=4 independent experiments); asterisks represent FDR adjusted p-values from one-sample t-tests comparing log2(RQ) to 0. **D** – Barplots showing expression changes of BM genes in H322 cells grown in 2D or 3D (spheroids) treated with 1000 U/ml interferon alpha for 72 h. BM – branching morphogenesis, LUAD – lung adenocarcinoma, IFN – interferon, SEM – standard error of mean, FDR – false discovery rate, AT2 – alveolar type 2 cell, **'.'**- p ≤ 0.1, * – p ≤ 0.05, ** – p ≤ 0.01, *** – p ≤ 0.001.
